# Specificity and Promiscuity at the Branch Point in Gentamicin Biosynthesis

**DOI:** 10.1016/j.chembiol.2014.03.005

**Published:** 2014-05-22

**Authors:** Junhong Guo, Fanglu Huang, Chuan Huang, Xiaobo Duan, Xinyun Jian, Finian Leeper, Zixin Deng, Peter F. Leadlay, Yuhui Sun

**Affiliations:** 1 Key Laboratory of Combinatorial Biosynthesis and Drug Discovery (Ministry of Education), and School of Pharmaceutical Sciences, Wuhan University, Wuhan, Wuchang 430071, People’s Republic of China; 2 Department of Biochemistry, University of Cambridge, Cambridge CB2 1GA, UK; 3 Department of Chemistry, University of Cambridge, Cambridge CB2 1EW, UK

## Abstract

Gentamicin C complex is a mixture of aminoglycoside antibiotics used to treat severe Gram-negative bacterial infections. We report here key features of the late-stage biosynthesis of gentamicins. We show that the intermediate gentamicin X2, a known substrate for C-methylation at C-6′ to form G418 catalyzed by the radical SAM-dependent enzyme GenK, may instead undergo oxidation at C-6′ to form an aldehyde, catalyzed by the flavin-linked dehydrogenase GenQ. Surprisingly, GenQ acts in both branches of the pathway, likewise oxidizing G418 to an analogous ketone. Amination of these intermediates, catalyzed mainly by aminotransferase GenB1, produces the known intermediates JI-20A and JI-20B, respectively. Other pyridoxal phosphate-dependent enzymes (GenB3 and GenB4) act in enigmatic dehydroxylation steps that convert JI-20A and JI-20B into the gentamicin C complex or (GenB2) catalyze the epimerization of gentamicin C2a into gentamicin C2.

## Highlights

• Dehydrogenase GenQ acts at the branch point in gentamicin biosynthesis
• Dehydrogenase GenQ has dual specificity and operates in both branches
• Aminotransferase GenB1 is the preferred (but not exclusive) partner of GenQ
• GenB3 and GenB4 are involved in 3′,4′-didehydroxylations, GenB2 is an epimerase

## Teaser

Gentamicin mixtures have been used for several decades to combat Gram-negative bacterial infections, but single components might offer a therapeutic advantage. Guo et al. identify five enzymes catalyzing key late steps in gentamicin biosynthesis, pointing the way to future production of single-component gentamicins.

## Introduction

**Figure 1:**
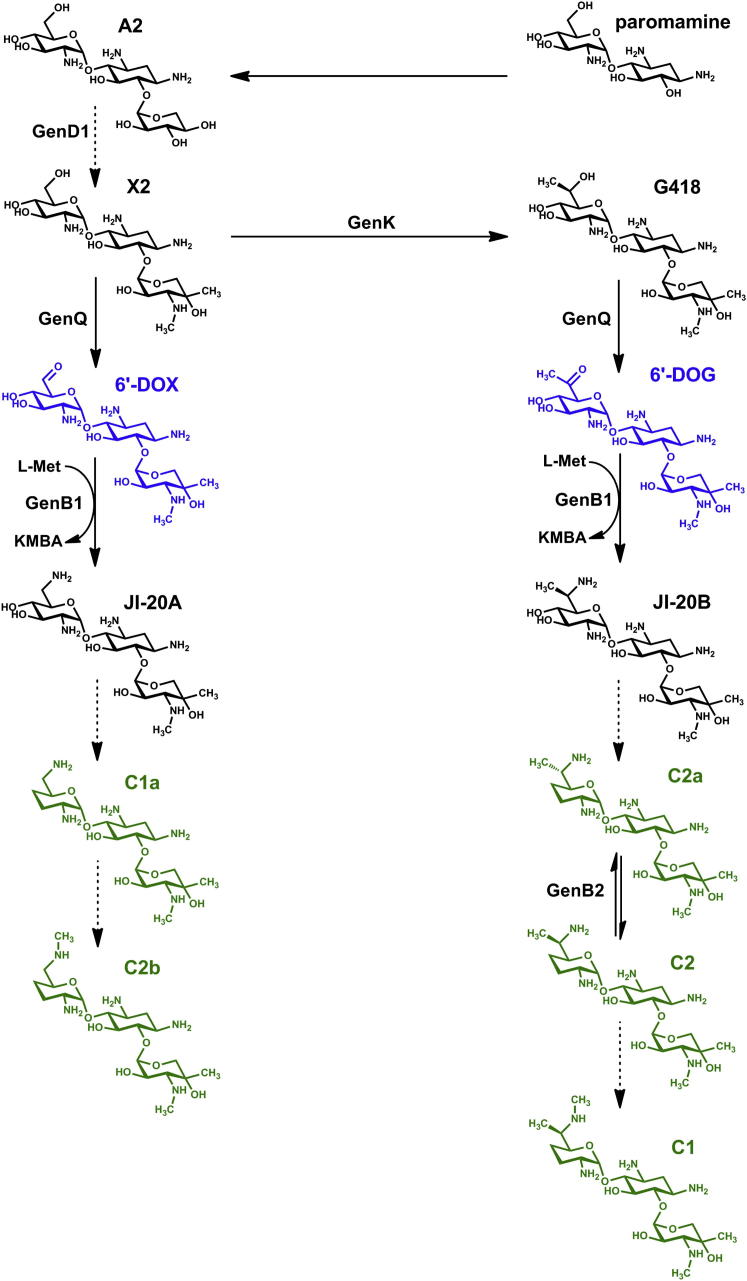
Proposed Biosynthetic Pathway from Paromamine to the Gentamicin C Complex The early steps in the pathway lead from the pseudodisaccharide paromamine to the branch point at gentamicin X2. The five main components of the clinically-used gentamicin C complex are shown in green. The proposed roles of the radical S-adenosylmethionine-dependent methyltransferases GenD1 and GenK are indicated. The role of GenK has been established by previous in vitro work. Intermediates 6′-DOX and 6′-DOG, newly-detected in this study, are shown in blue. The roles of dehydrogenase GenQ and aminotransferase GenB1 are revealed in this study, as well as the involvement of GenB3 and GenB4 in the steps from intermediates JI-20A and JI-20B to the gentamicin C complex, and of GenB2 in epimerization of gentamicin C2a to gentamicin C2. For further details see the text. KMBA, 2-keto-3-methylthiobutyric acid; 6′-DOG, 6′-dehydro-6′-oxo-G418; 6′-DOX, 6′-dehydro-6′-oxo-gentamicin X2.

Gentamicins are clinically valuable aminoglycoside antibiotics containing modified sugar units, including an unusual aminocyclitol ring (2-deoxystreptamine [2-DOS]; Houghton et al., 2010; Park et al., 2013). Gentamicins inhibit protein synthesis by interfering with initiation, codon fidelity, and translocation. They are isolated from the filamentous bacterium *Micromonospora echinospora* as gentamicin C complex, a mixture of five components (Figure 1). Gentamicin C complex is a mainstay of the treatment of life-threatening sepsis caused by Gram-negative bacterial infections. It also shows promise as a lead for treatment of inherited diseases associated with premature stop codons (Hainrichson et al., 2008; Linde and Kerem, 2008) and acts as a sensitizing agent for NCI-H460 lung cancer cells, increasing the efficacy of various anticancer agents (Cuccarese et al., 2013). Unfortunately, these vital drugs entail a serious risk of kidney damage and hearing loss (Bockenhauer et al., 2009). Most studies on the mechanisms of gentamicin toxicity have used the commercial mixture, despite evidence that an individual component of the gentamicin mixture may have lower toxicity (Sandoval et al., 2006; Kobayashi et al., 2008). As a prerequisite for engineering to favor the production of a particular gentamicin, we have undertaken a detailed investigation of the late steps in the pathway.

The outlines of the gentamicin pathway were established by isolating blocked mutants after random mutagenesis and monitoring the fate of compounds fed to such blocked strains (Testa and Tilley, 1975, 1976; Kase et al., 1982a, 1982b). It was shown that the pathway branches at gentamicin X2 (Figure 1) and the final steps involve a series of oxidations, transaminations, and C- and N-methylations, as well as the intriguing loss of two hydroxyl groups from one of the sugar rings. Many biosynthetic gene clusters that govern aminoglycoside biosynthesis have been sequenced (Ota et al., 2000; Unwin et al., 2004; Kharel et al., 2004a, 2004b, 2004c; Huang et al., 2005; Subba et al., 2005; Aboshanab, 2005; Hong et al., 2009). As a result, rapid progress has been made in identifying the common enzymatic steps that lead to the 2-DOS scaffold, and thence to the pseudodisaccharide paromamine (Figure 1; Llewellyn and Spencer, 2006; Thibodeaux et al., 2008; Kudo and Eguchi, 2009; Kudo et al., 2009).

The intermediate pseudotrisaccharide gentamicin A2 (Figure 1) has been produced by heterologous expression of a subset of the gentamicin biosynthetic genes in the host strain *Streptomyces venezuelae* (Park et al., 2008). This has enabled a sharper focus on those genes likely to govern the later steps of gentamicin biosynthesis. Specific late gene knockouts have been made in *genD1* and *genK*, both of which are predicted based on bioinformatic analysis (Figure S1A available online) to encode class B radical S-adenosylmethionine-dependent C-methyltransferases (Zhang et al., 2012). The replacement of *genD1*, also known as *gntE* (Unwin et al., 2004) or *gtmI* (Kharel et al., 2004c), by a thiostrepton resistance gene (*tsr*) led to accumulation of gentamicin A2 (Figure 1; Kim et al., 2008). These authors interpreted their result as meaning that GenD1 catalyzes N-methylation at the 3″-NH_2_ rather than C-methylation at C-4″ of the xylose ring of gentamicin A2; but this, and the exact order of events, remains to be established. Disruption of *genK*, also known as *gntK* (Unwin et al., 2004) or *gacD* (Kharel et al., 2004c), has been shown to result in preferential accumulation of gentamicin C1a (Karki et al., 2012; Hong and Yan, 2012; Li et al., 2013). GenK has also been demonstrated to catalyze C-methylation in vitro of C-6′ in gentamicin X2 to form G418 (Kim et al., 2013).

By analysis of the gentamicin-related metabolites accumulated in strains bearing single and multiple specific gene deletions, by following the bioconversion of specific intermediates fed to such mutants, and by assaying individual steps using purified recombinant enzymes, we demonstrate that control at the branch point is shared between GenK and the flavin-linked dehydrogenase GenQ. Importantly, GenQ operates in both branches of the pathway, and is essential for the oxidation of both X2 and G418 (Figure 1). We have also obtained fresh insights into the respective catalytic contributions of four pyridoxal phosphate-dependent enzymes GenB1, GenB2, GenB3, and GenB4.

## Results

### GenK Acts at the X2 Branch Point and Is Essential for the Production of G418 and Gentamicins C2a, C2, and C1

**Figure 2:**
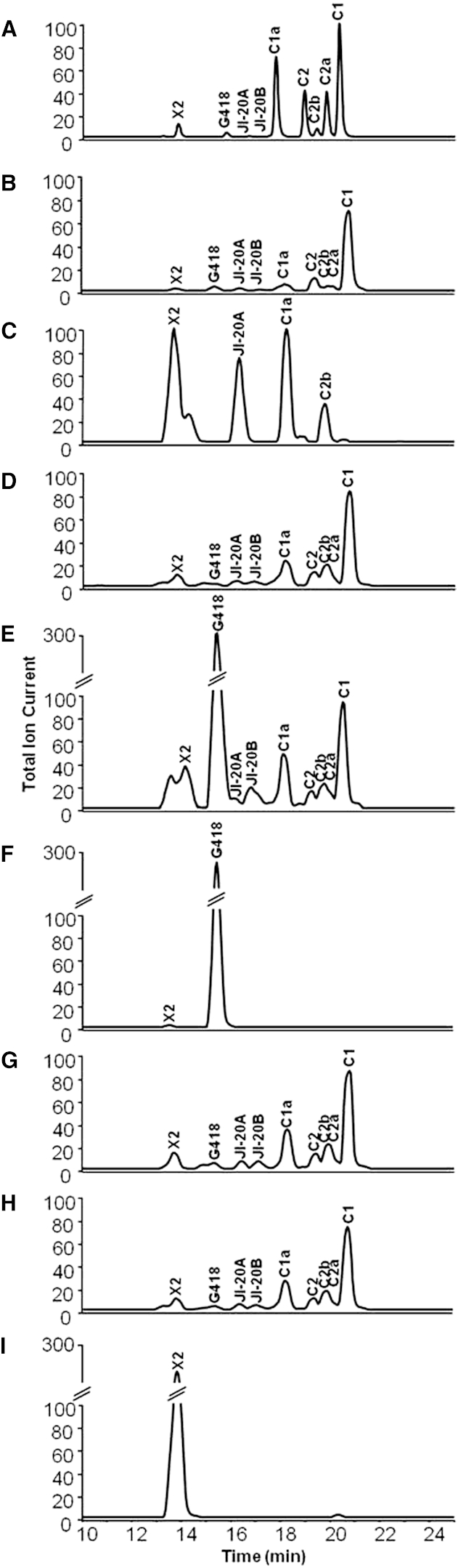
LC-ESI_HRMS Analysis of the Production of Gentamicin C Complex and Gentamicin-Related Intermediates by *Micromonospora echinospora* (A) gentamicin C standard mixture. (B) Wild-type. (C) ΔgenK mutant. (D) ΔgenK::*genK* mutant. (E) ΔgenK mutant fed with G418. (F) ΔgenQ mutant. (G) ΔgenQ::*genQ* mutant. (H) ΔgenQ::*neo11* mutant. (I) ΔgenQΔgenK mutant. See also Figures S1–S3. For details of the LC-ESI-HRMS analysis, see the Supplemental Information.

To confirm the previously established role of *genK* at the gentamicin X2 branch point, this gene was knocked out by targeted in-frame deletion (Figure S2(1)A). The mutant was confirmed by PCR and Southern hybridization (Figures S2(1)B and S2C). Liquid chromatography-electrospray ionization high-resolution mass spectrometry (LC-ESI-HRMS) analysis confirmed that in this mutant, production of G418, JI-20B, and gentamicins C1, C2, and C2a was abolished (Figure 2C and Figure S3B). In contrast, levels of gentamicin X2, JI-20A, and gentamicins C1a and C2b were all substantially increased compared to wild-type (Figure 2C and Figure S3B). Complementation of the ΔgenK mutant, carried out by using plasmid pWHU67 containing *genK* under the control of the P*ermE*^∗^ promoter (Figure S2(4)A), restored the production of gentamicin C complex and of various intermediates to wild-type levels (Figure 2D). Chemical complementation of the ΔgenK mutant by feeding G418 also restored production of JI-20B, gentamicin C2, C2a (the C-6′ epimer of C2), and C1 (Figure 2E).

### Dehydrogenase GenQ Is Essential in Both Branches of the Gentamicin Pathway that Diverges from X2

The gene *genQ* (also known as *gntX* (Unwin et al., 2004) or *gacJ* (Kharel et al., 2004c)) is predicted to encode a flavin-linked dehydrogenase with significant sequence identity to enzymes catalyzing similar steps in other aminoglycoside pathways (Figure S1B). GenQ was therefore an attractive candidate to act at the X2 branch point and initiate the biosynthesis of C1a and the minor component C2b (also known as sagamicin; Figure 1). To investigate the role of *genQ*, a 1494 bp internal DNA fragment was deleted in-frame (Figure S2(2)A). The mutant was confirmed by PCR and Southern hybridization (Figures S2(2)B and S2C). LC-ESI-HRMS analysis revealed that production was abolished in the ΔgenQ mutant not only of JI-20A and the downstream gentamicins C1a and C2b, but unexpectedly also of JI-20B and gentamicins C2, C2a, and C1 (Figure 2F and Figure S3B). Meanwhile, G418 was accumulated to levels 100-fold higher than wild-type (Figure S3B). These data strongly suggest that *genQ* is not only involved at the X2 branch point in the first step toward C1a and C2b, but is also essential in the other branch of the pathway for the oxidation of G418 that leads on to JI-20B and finally to C2, C2a, and C1. The LC-ESI-HRMS analysis did not detect 6′-dehydro-6′-oxo-G418 (6′-DOG) or 6′-dehydro-6′-oxo-gentamicin X2 (6′-DOX; Figure 1), the presumed products of GenQ-catalyzed oxidation.

The dual function of *genQ* was further supported by showing that the ΔgenQ mutant could be homologously complemented by introduction of pWHU163, in which *genQ* is under the control of the constitutive promoter P*ermE*^∗^ (Figure 2G and Figure S2(4)B). This mutant was also heterologously complemented by introduction of pWHU165 (Figure 2H and Figure S2(4)C) containing *neo11*, a gene from the neomycin biosynthetic pathway that is homologous to *genQ* (Huang et al., 2007). LC-ESI-HRMS analysis of fermentation extracts from the mutants ΔgenQ::*genQ* and ΔgenQ::*neo11* demonstrated that all components of the gentamicin C complex were restored to wild-type levels (Figures 2G and 2H). The ΔgenQΔgenK mutant was also generated by targeted in-frame deletions (Figure S2(3)), and LC-ESI-HRMS analysis showed that, as expected, only gentamicin X2 was accumulated, at levels over 100-fold higher than in the wild-type (Figure 2I and Figure S3B).

### GenB1 Is the Chief Aminotransferase Partner for GenQ while GenB3 and GenB4 Participate in Dehydroxylation

**Figure 3:**
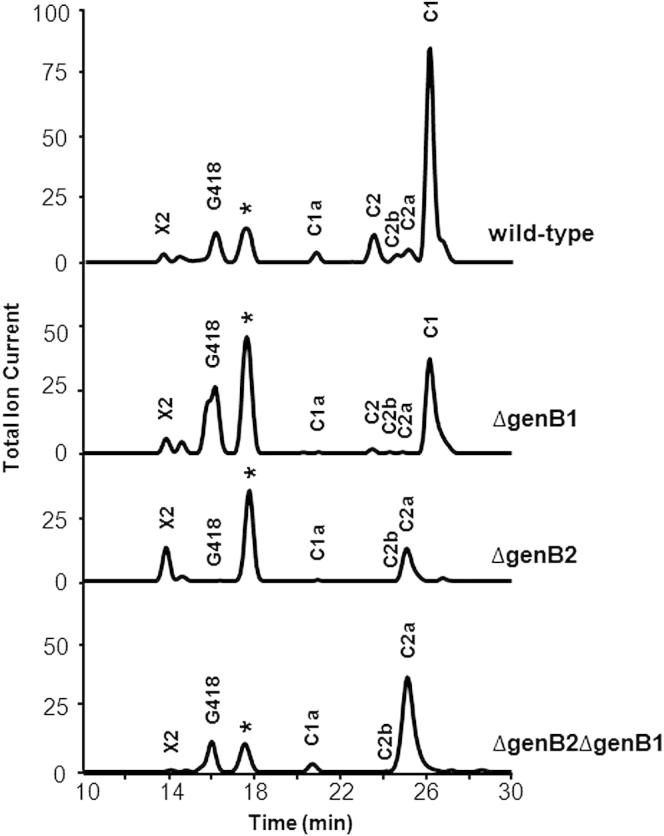
Production of Gentamicin C Components and Related Intermediates by *M. echinospora genB* Mutants Data are shown for three *genB* mutants that continue to produce gentamicin C components. Data for a further 12 *genB* mutants (Figure S4B) that were found to produce no gentamicin C complex are shown in Table S1. The starred peak is from an unknown metabolite with the same mass as gentamicin X2.

The gentamicin biosynthetic gene cluster in *M. echinospora* contains four genes that appear to encode class III pyridoxal phosphate-dependent transaminases (Figure S4A), any of which might catalyze the transamination steps that lead in parallel to JI-20A and JI-20B, respectively. The *genB1* gene was deleted in-frame, and the identity of the resulting mutant was confirmed by PCR and Southern blot analysis (Figure S4B(1)). Similarly, *genB2*, *genB3*, and *genB4* were each individually disrupted by in-frame deletion (Figures S4B(2) to ∼S4B(4)). Starting from these mutants, similar methods were used to generate all six double mutants (Figure S4B(5) to ∼S4B(10)), all four triple mutants (Figures S4B(11) to ∼S4B(14)), and the quadruple mutant ΔgenB4ΔgenB1ΔgenB3ΔgenB2 (Figure S4B(15)). Each of these mutant strains was fermented in liquid medium, as detailed in the Experimental Procedures section, and gentamicin C complex and related metabolites were extracted, purified by ion exchange and submitted to LC-ESI-HRMS analysis (Figure 3 and Table S1) for comparison with wild-type.

This analysis revealed that in the quadruple mutant only X2 and G418 were produced (the latter at levels about 18-fold higher than in wild-type), a result that argues against any contribution to transamination activity from enzymes other than these four. In fact of the 15 mutants examined (Table S1), only ΔgenB1, ΔgenB2, and the double mutant ΔgenB2ΔgenB1 produced any gentamicin C complex components (Figure 3). Deletion of *genB1* substantially lowered, but did not abolish, the levels of individual components of the gentamicin C complex. This argues strongly that at least one other aminotransferase can partially fulfill the role of GenB1. Nevertheless, these data are consistent with GenB1 making the chief contribution to amination of both 6′-DOX and 6′-DOG. In the ΔgenB2 mutant and in the ΔgenB2ΔgenB1 double mutant, levels of gentamicins C1a and C2b in the “left-hand” branch (Figure 1) were significantly reduced. In the “right-hand” branch, production of C2a was increased more than 7-fold, while C2 and C1 production was completely blocked. Initially, we identified the C2a peak (Figure 3) as C2b from the “left-hand” branch (these metabolites have similar retention times, the same mass and the same MS-MS fragmentation pattern). However, further analysis and spiking with authentic C2a, C2, and C1 confirmed that C2a is the product (Figure S5). This finding leads directly to a proposal for the role of GenB2 in the pathway (see the Discussion section). All *genB* mutants continued to produce the known late-stage biosynthetic intermediates: gentamicin X2 at the branch point, G418 and JI-20B on the “right-hand” branch, and JI-20A on the “left-hand” branch of the pathway (Figure 1) at approximately wild-type levels (Figure 3 and Table S1). Again, these results show that other aminotransferases can substitute for GenB1.

Deletion of either *genB3* or *genB4*, alone or in combination with other *genB* genes, abolished the production of all five gentamicin C compounds (Table S1). For the ΔgenB3 mutant, both JI-20A and JI-20B were present at higher (more than 10-fold) levels than in wild-type, consistent with the likely involvement of GenB3 in the enigmatic dehydroxylation steps of gentamicin biosynthesis, rather than in amination of X2 and G418. In the ΔgenB4 mutant, too, and also in the double mutant ΔgenB4ΔgenB3, the C complex was completely lacking whereas intermediates X2, G418, JI-20A, and JI-20B were still present at either wild-type or slightly elevated levels. This demonstrated that GenB4 also has an essential role in dehydroxylation, and that this role is distinct from that of GenB3. Other double mutants showed different levels of intermediates X2, G418, JI-20A, and JI-20B that reflect the differing contribution of each GenB enzyme to transamination. When the triple mutants were examined, in which only one of the four *genB* genes remains functional, ΔgenB2ΔgenB4ΔgenB3 was found to accumulate wild-type levels of JI-20A and high levels of G418 and JI-20B, confirming that the presence of GenB1 alone suffices for the reaction of both X2 and G418. The other triple mutants all showed very low levels of both JI-20A and JI-20B compared to wild-type, again indicating a minor role for GenB2, GenB3, or GenB4 in catalyzing either transamination.

### Recombinant GenQ Catalyzes 6′-Oxidation of G418 and Gentamicin X2

Recombinant GenQ was expressed in *Escherichia coli* to assay its ability to catalyze the 6′-dehydrogenation of both gentamicin X2 and G418. GenQ as published is shorter (∼40 amino acid residues) at the N terminus than close homologs in other aminoglycoside biosynthetic pathways (Figure S1B). We therefore amplified two longer versions of *genQ*: One, designated LM_*genQ*, starts from the rare start codon CTG (Missiakas et al., 1993) and provides an extra 19 N-terminal residues, whereas the second version, VM_*genQ*, provides an extra 38 N-terminal residues absent in the original GenQ protein (Figure S6A). N-His_6_-tagged VM_GenQ was expressed as a soluble protein in *E. coli* (Figure S6B). Purified VM_GenQ had a yellow color and major absorption peaks at 370 nm and 445 nm (Figure S6C), indicating the presence of a flavin prosthetic group. A solution of VM_GenQ was deproteinized and flavin adenine dinucleotide (FAD) (*m*/*z* 786 [M+H]^+^ and *m*/*z* 808 [M+Na]^+^) was detected in the supernatant with LC-ESI-MS analysis. LM_GenQ did not contain bound flavin, so was not studied further.

**Figure 4:**
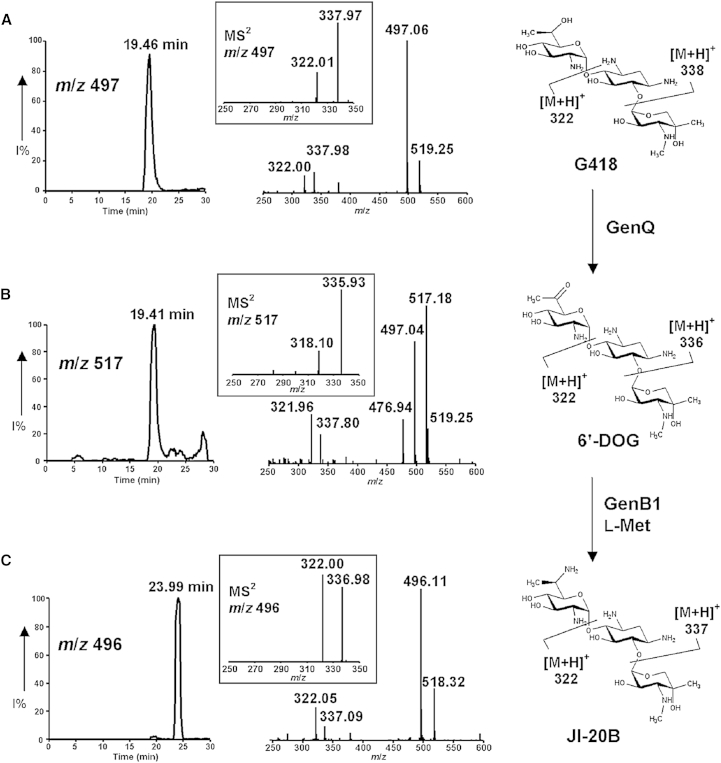
LC-MS Analysis of GenQ/GenB1-Catalyzed Dehydrogenation and Transamination of G418 Selective ion monitoring was carried out on (A) [M+H]^+^ (*m*/*z* 497) and [M+Na]^+^ (*m*/*z* 519) ions of G418; (B) the [M+Na]^+^ (*m*/*z* 517) ion of 6′-DOG, the product of GenQ-catalyzed 6′-dehydrogenation of G418; (C) [M+H]^+^ (*m*/*z* 496) and [M+Na]^+^ (*m*/*z* 518) ions of JI-20B, the product of GenB1 catalyzed 6′-amination of 6′-DOG using l-methionine as an amino donor. MS/MS analysis of [M+H]^+^ (*m*/*z* 497), [M+Na]^+^ (*m*/*z* 517), and [M+H]^+^ (*m*/*z* 496) ions is also shown. The peak at *m*/*z* 318.1 in Figure 4B arises by further loss of water from fragment *m*/*z* 335.93. See also Figures S6 and S7.

**Figure 5:**
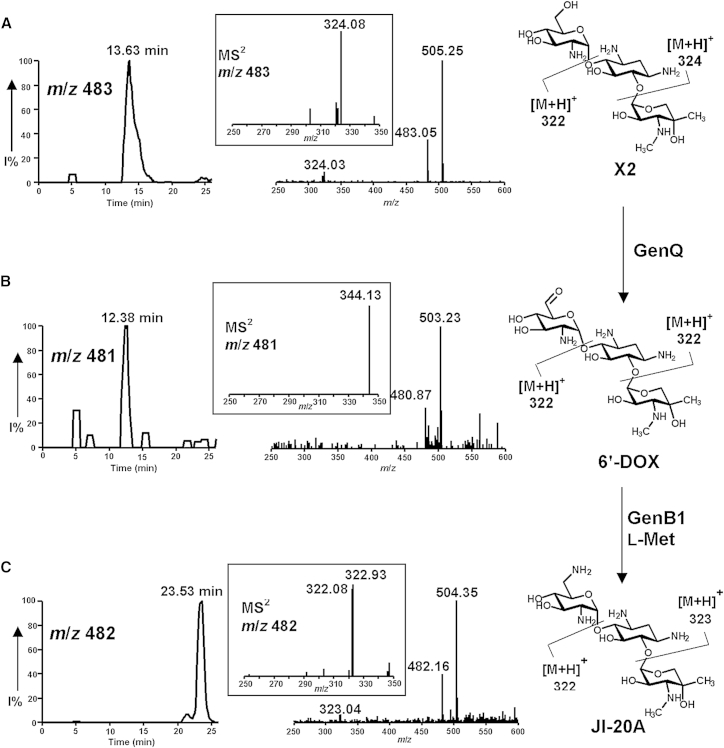
LC-MS Analysis of GenQ/GenB1-Catalyzed Dehydrogenation and Transamination of Gentamicin X2 Selective ion monitoring was carried out on (A) [M+H]^+^ (*m*/*z* 483) and [M+Na]^+^ (*m*/*z* 505) ions of gentamicin X2; (B) [M+H]^+^ (*m*/*z* 481) and [M+Na]^+^ (*m*/*z* 503) ions of 6′-DOX, the product of GenQ-catalyzed 6′-dehydrogenation of gentamicin X2; (C) [M+H]^+^ (*m*/*z* 482) and [M+Na]^+^ (*m*/*z* 504) ions of JI-20A, the product of GenB1-catalyzed 6′-amination of 6′-DOX using l-methionine as an amino donor. MS/MS analysis of [M+H]^+^ (*m*/*z* 483), [M+H]^+^ (*m*/*z* 481) and [M+H]^+^ (*m*/*z* 482) ions are also shown. See also Figures S6 and S7.

VM_GenQ (hereafter referred to as GenQ) was tested for dehydrogenase activity using G418 and gentamicin X2 as substrates, as described in the Experimental Procedures. LC-ESI-MS analysis of the reaction mixture containing GenQ and G418 revealed a new peak eluting at a slightly earlier retention time, with *m*/*z* = 495 [M+H]^+^ and *m*/*z* = 517 [M+Na]^+^; Figure 4B), two mass units less than G418 (Figure 4A), as expected for the conversion of G418 into 6′-DOG. GenQ also showed activity with X2 as the substrate (Figure 5A), producing a compound with the mass expected of 6′-DOX (*m*/*z* = 481 [M+H]^+^, *m*/*z* = 503 [M+Na]^+^; Figure 5B). Tandem mass spectrometry (MS-MS) analysis of the species with *m*/*z* 517 and *m*/*z* 481 gave fragmentation patterns fully consistent with dehydrogenation having occurred in the side chain of the 6′-methyl-glucosamine ring of G418 and in the glucosamine ring of X2, respectively.

### Multiple Aminotransferases Are Capable of C-6′-Transamination of Gentamicin Biosynthetic Intermediates In Vitro

The gene products of *genB1*, *genB2*, *genB3*, and *genB4* were all considered plausible candidate enzymes to partner GenQ in 6′-dehydrogenation-transamination, based on their significant sequence similarity to Neo18, a previously-characterized glutamate: 6′-dehydroparomamine aminotransferase (Huang et al., 2007; Figure S4A). Recombinant GenB1, GenB2, GenB3, and GenB4 were expressed in *E. coli* (Figure S6B). All four purified proteins were yellow and had UV-visible absorption spectra typical of aminotransferases containing bound pyridoxal-5′-phosphate cofactor (Figure S6D). GenB1, GenB2, GenB3, and GenB4 were each tested for their ability to catalyze the transamination of the aldehyde 6′-DOX to form JI-20A; and to catalyze transamination of the ketone 6′-DOG to form JI-20B. In these coupled assays, carried out as detailed in Experimental Procedures, l-ornithine, *N*-acetyl-l-ornithine, d-arginine, S-adenosyl-l-methionine, and all proteinogenic amino acids except l-proline were tested as potential amino donors. The reaction mixtures were analyzed with LC-ESI-MS (Figures 4, 5, and S7).

GenQ and GenB1 together catalyzed the conversion of G418 to JI-20B, with l-methionine as the most effective amino donor (80% conversion) (Figure 4 and S7Ai). Other amino donors tested except l-threonine and l were also accepted as substrates by GenB1, but the yield of JI-20B never exceeded 37% conversion. When GenB2 was used instead of GenB1, low level of conversion to JI-20B was observed when either l-Lys (7%), l-Met (9%) or l-Tyr (16%) was used as amino donor (Figure S7Aii). GenB3 also showed some activity (17% conversion), but only with l-Gln as the amino donor (Figure S7Aiii). GenB4, in contrast, did not show activity with any of the amino donors tested (Figure S7Aiv).

**Figure 6:**
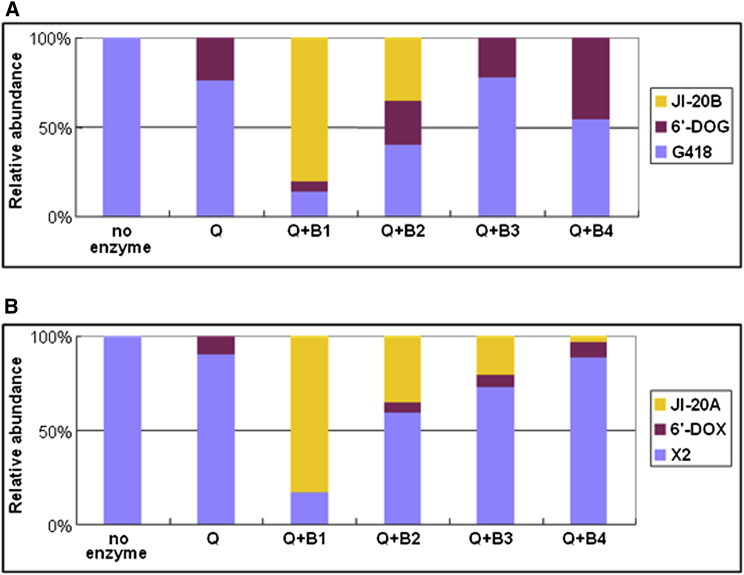
Coupled In Vitro Analysis of Aminotransferase Activities of Individual GenB Enzymes (A) Amination of 6′-DOG generated in situ from G418 by the reaction of GenQ. (B) Amination of 6′-DOX generated in situ from gentamicin X2 by the reaction of GenQ; Reaction mixtures were analyzed after incubation for 16 hr. The amino donor was L-methionine. See also Figures S6 and S7.

The combination of GenB1 and GenQ also catalyzed the conversion of gentamicin X2 into JI-20A, with l-Met and l-Orn as the only effective amino donors (54% and 70% conversion, respectively; Figure 5 and S7Av). GenB2 showed marginal activity (<5% conversion) on 6′-DOX, and only when l-Gln, l-Lys, l-Met, or l-Phe were used as the amino donors (Figure S7Avi). GenB3 accepted all amino donors tested with l-Orn giving ∼50% conversion; Figure S7Avii). Gentamicin X2 was a very poor substrate (<6% conversion) for the combination of GenQ and GenB4 (Figure S7Aviii). When the aminotransferase activities of GenB1, GenB2, GenB 3, and GenB4 on 6′-DOG and 6′-DOX in the presence of l-methionine were compared, GenB1 clearly showed the highest activity, on both substrates (Figure 6). Although it cannot be ruled out that another amino acid donor is preferred in vivo, the robust activity seen with l-methionine in vitro suggests that this is likely to be the physiological substrate.

### GenB2 Catalyzes In Vitro Epimerization of Gentamicin C2 and Gentamicin C2a

Given the evidence that the chief role of GenB2, GenB3, and GenB4 is in the unexplored dehydroxylation stage of gentamicin C biosynthesis, we investigated their possible involvement in the generation of gentamicin C2a, which like G418 and gentamicins C2 and C1 is methylated at the C-6′ position but which has the opposite configuration at this stereocenter (Figure 1). Purified recombinant GenB enzymes were assayed for their ability to catalyze the epimerization of gentamicin C2 into C2a, as described in the Experimental Procedures. GenB1, GenB3, and GenB4 showed no activity, as judged by LC-ESI-MS analysis of reaction mixtures. In contrast, GenB2 did catalyze the epimerization of C2, to form a mixture of C2 and C2a in a molar ratio of 1:2 (Figure S7B). Furthermore, when C2a was used as the substrate the reverse reaction was also detected and the final product mixture was found to contain the C2 and C2a epimers in the same molar ratio of 1:2 (Figure S7B), implying that equilibrium had been reached.

## Discussion

The results presented here significantly advance our understanding of the biosynthetic pathway to the gentamicin C complex, an established drug used to combat life-threatening infections. The task of assigning functions to individual gene products involved in the pathway is not trivial because the cluster contains multiple, mutually homologous genes for methyltransferases, oxidoreductases, and aminotransferases with potential for overlapping substrate specificities. There are numerous precedents from other aminoglycoside pathways for enzyme promiscuity or even dual function (Huang et al., 2005, 2007; Fan et al., 2008; Yokoyama et al., 2008; Park et al., 2011). From our analysis of the ΔgenQΔgenK mutant it is clear that GenK and GenQ uniquely control the fate of gentamicin X2 at the biosynthetic branch point. No other enzymes in *M. echinospora* were able to take over these roles under the fermentation conditions used. This is in contrast to (for example) the neomycin biosynthetic pathway, where the GenQ homolog Neo11 is proposed to function both as a 6′-paromamine dehydrogenase and then later in the pathway as the C-6″′-dehydrogenase acting on ring IV of neomycin (Llewellyn and Spencer, 2006; Huang et al., 2007; Kudo et al., 2009; Clausnitzer et al., 2011).

We have confirmed the important findings from several laboratories (Karki et al., 2012; Hong and Yan, 2012; Li et al., 2013) that the flux in the ΔgenK mutant is diverted exclusively down the “left-hand” branch (Figure 1) to C-6′-unmethylated metabolites JI-20A, gentamicin C1a, and gentamicin C2b; and we have shown that chemical complementation of this mutant with G418 fully restores the flux down the “right-hand” branch (Figure 1) to wild-type levels. In contrast to the results with the ΔgenK mutant, the ΔgenQ mutant was unexpectedly found to be blocked in both branches of the pathway, accumulating both X2 and G418. This observation rules out any other dehydrogenase from the gentamicin cluster (such as GenD2 or GenD3) being capable of specific C-6′-dehydrogenation of G418. This observation also showed that it would not be possible by deletion of *genQ* to enhance selective production of those gentamicin C complex components synthesized via the “right-hand” branch.

We next turned our attention to the potential roles of the four genes predicted to encode Class III pyridoxal phosphate-dependent aminotransferases: *genB1*, *genB2*, *genB3*, and *genB4*. We anticipated that there might be considerable substrate overlap and promiscuity shown by these enzymes, and we therefore constructed and analyzed the complete combinatorial set of 15 mutants in which one, two, three, or all four of these genes had been independently deleted in-frame. LC-ESI-HRMS analysis of these mutant strains revealed several important insights into the enzymology of gentamicin biosynthesis.

First, the quadruple mutant ΔgenB4ΔgenB1ΔgenB3ΔgenB2 was unable to produce any intermediate more advanced than X2 and G418, which implies that one or more of these genes encodes an aminotransferase that partners GenQ. Analysis of the triple mutants in which only one of these four was active revealed a major role for GenB1 in partnering the GenQ dehydrogenase in the amination of both X2 and G418, but also revealed a low-level promiscuity of the other GenB enzymes that allows them partially to replace GenB1 in the amination steps.

The analysis of mutants lacking either *genB3* or *genB4* showed that biosynthesis of all members of the gentamicin C complex was blocked, with accumulation of the known intermediates JI-20A and JI-20B. GenB3 and GenB4 appear therefore to participate, perhaps as pyridoxal-dependent dehydratases, in the multistep removal of the two unactivated hydroxyl groups from one of the sugar rings. Deletion of *genB2* markedly lowered production of gentamicins C1a and C2b in the “left-hand” branch, perhaps through build-up of inhibitory metabolites; and blocked the production of C2 and C1, two of the three components of the gentamicin C complex that are produced in the “right-hand” branch of the pathway. The level of C2a in contrast rose over 7-fold, clearly implicating this stereoisomer as the first-formed product of didehydroxylation of JI-20B and identifying GenB2 as the catalyst for its epimerization to C2 (Figure 1).

We expressed and affinity-purified GenQ from recombinant *E. coli* as a soluble protein containing FAD. By using G418 as substrate for this enzyme, we have obtained LC-ESI-MS and MS-MS evidence for the production of the putative ketone intermediate 6′-DOG. Analogously, when GenQ was incubated with X2 as substrate the observed MS-MS fragmentation spectrum of the product was consistent with that expected for the predicted aldehyde intermediate 6′-DOX. We also expressed and purified GenB1, GenB2, GenB3, and GenB4 from recombinant *E. coli* as soluble enzymes containing pyridoxal phosphate, and this allowed us to reconstitute amination from X2 to JI-20A and from G418 to JI-20B respectively, by coupling the GenQ-catalyzed reaction to subsequent transamination catalyzed by one of these four enzymes. GenB1 proved far superior to the other three GenB enzymes in catalyzing these in vitro conversions, with l-methionine consistently the best amino donor among the many amino acids screened. These results are in good agreement with the data from our fermentation of GenB-deficient mutants (Table S1). From these results we conclude that GenB1 is the major, though not the exclusive, partner for GenQ in the dehydrogenation-transamination of both X2 and G418.

GenB3 and GenB4 are likely to cooperate with other enzymes in the 3′,4′-didehydroxylation of both JI-20A and JI-20B. Recent in vitro evidence using a substrate analog (Shao et al., 2013) implicates the phosphotransferase GenP in initiating this process, through phosphorylation at the C-3′ hydroxy group. Sisomicin, an antibiotic naturally produced by *M. inyoensis* (Reimann et al., 1974), is a 4′,5′-dehydro-derivative of gentamicin C1a. It has also been described as a minor component of the gentamicin fermentation of *M. echinospora* (Bérdy et al., 1977). When added to resting cells of mutant *M. echinospora*, *M. sagamiensis* (Kase et al., 1982b), or *M. rhodorangea* (Lee et al., 1977) blocked early in the gentamicin pathway, sisomicin is reportedly converted into gentamicin C1a and C2b. Analogously, verdamicin C2a (4′,5′-dehydro-gentamicin C2a) is an antibiotic naturally produced by *M. grisea* (Weinstein et al., 1975), which reportedly can be bioconverted by an early stage blocked mutant of *M. sagamiensis* (Kase et al., 1982b) into gentamicins C2a, C2, and C1. It remains to be determined how closely sisomicin and verdamicin are related to genuine intermediates in the normal 3′,4′-didehydroxylation pathway that leads to gentamicins.

A further open question has been the origin of gentamicin C2a, the minor C-6′ epimer of C2. A blocked mutant of *M. sagamiensis* (KY 11564) has been described (Kase et al., 1982a), which accumulates gentamicin C2a but neither C2 nor C1. Another mutant that appears to be blocked earlier in the pathway bioconverts verdamicin C2a into gentamicin C2a then into C2 and C1 (Kase et al., 1982b). From the results obtained here from fermentation of the ΔgenB2 mutant, it is clear that this gene is required for formation of C2 but not for C2a. We investigated a possible role for GenB1, GenB2, GenB3, or GenB4 in interconverting gentamicins C2 and C2a. Remarkably, purified GenB2 (but not the others) proved competent in vitro to catalyze the 6′-epimerization of gentamicin C2a into gentamicin C2 (and vice versa). This evidence strongly supports the original proposal (Kase et al., 1982b) that C2a is an obligatory precursor to C2, which in turn implies that two inversions of the configuration at the C-6′ position occur during the transformation of G418 into gentamicin C2.

## Significance

**Aminoglycosides, mainly produced by actinobacteria, constitute a widespread and versatile group of bioactive natural products. Examples of clinically used aminoglycosides include gentamicin to treat neonatal sepsis; kanamycin and amikacin to treat MRSA; and paromomycin to treat visceral leishmaniasis. Aminoglycosides are also under active investigation for their anti-HIV properties and in attempts to rescue certain genetic conditions by allowing readthrough of premature stop codons. Gentamicin in particular is of continuing interest despite its known toxicity because of its remarkable potency in combating systemic Gram-negative infections. The perspective of a safer and less expensive gentamicin through administration of a single component, or using a single component for semisynthesis of a novel derivative, is therefore very attractive. The work we report here has used complementary in vivo and in vitro approaches to identify the key enzymes that control the branch point in the gentamicin biosynthetic pathway. It has also revealed the involvement of the pyridoxal phosphate-dependent enzymes GenB2, GenB3, and GenB4 in the subsequent steps. By more closely defining the molecular enzymology of the pathway in *Micromonospora echinospora*, this work has brought closer the goal of assembling a defined set of pathway enzymes that might in future deliver single gentamicin C components in a scaleable and sustainable synthetic biology process.**

## Experimental Procedures

### Bacterial Strains, Chemicals, and Culture Conditions

*E. coli* DH10B and NovaBlue strains were used as cloning hosts, and *E. coli* BL21(DE3) for protein expression. *E. coli* ET12567/pUZ8002 was used for intergeneric conjugation between *E. coli* and *Streptomyces* (MacNeil et al., 1992). *Streptomyces fradiae* NCIMB 8233 (Waksman and Lechevalier, 1949) was the source of template DNA for cloning the *neo11* gene. *M. echinospora* ATCC15835 (Weinstein et al., 1963) was used for creation of in-frame deletion mutants and as the source of *gen* genes. Restriction endonucleases, Phusion High-Fidelity Master Mix with GC-buffer, and T4 DNA ligase were from NEB England Biolabs. *Pfu* DNA polymerase was from Fermentas. Oligonucleotide primers were synthesized by either GenScript or Invitrogen. DNA sequencing of PCR products was performed by GenScript or by the Department of Biochemistry DNA Sequencing Facility, University of Cambridge. DIG DNA labeling and detection kits were from Roche. G418 used for feeding studies was obtained from GIBCO. For in vitro enzyme assays, G418 and amino acids were from Sigma-Aldrich, gentamicin X2 was from Toku-E, and *S*-adenosyl methionine was from Carbosynth.

*M. echinospora* ATCC15835 wild-type and mutants were grown in ATCC Medium 172 (glucose 10 g, soluble starch 20 g, yeast extract 5 g, N-Z amine type A 5 g, CaCO_3_ 1 g, 1 l distilled water) for chromosomal DNA isolation and preparation of mycelium. *E. coli* strains were maintained in 2 × TY or LB media at 37°C with the appropriate antibiotic selection at a final concentration of 25 μg/ml apramycin, 100 μg/ml, carbenicillin, 25 μg/ml chloramphenicol, 25 μg/ml or 50 μg/ml kanamycin, 15 μg/ml tetracycline, or 25 μg/ml thiostrepton.

For production of gentamicin C complex and intermediates, *M*. *echinospora* ATCC15835 and its mutants were cultured in two stages. A seed culture was maintained in liquid ATCC 172 medium at 28°C with shaking at 220 rpm for 2 days before being inoculated into fresh liquid ATCC 172 Medium. The production fermentation was incubated at 28°C with shaking for 8 days. For feeding experiments, filter-sterilized compounds (50 μg/ml) were added to the medium just before addition of the seed culture.

### Construction of Gene Disruption Plasmids

For in-frame deletion, DNA fragments flanking each target gene were amplified from the genomic DNA of *M. echinospora* ATCC15835 (see List of Primers Used in the Supplemental Information). The PCR products were each cloned into pUC18, then cut out and cloned together into the *Streptomyces*-*E. coli* shuttle vector pYH7 (Sun et al., 2009) to obtain the following gene disruption plasmids: pYH286 (for ΔgenQ), pWHU1 (for ΔgenK), pWHU4 (for ΔgenB1), pWHU2 (for ΔgenB2), pWHU5 (for ΔgenB3), and pWHU3 (for ΔgenB4; Table S2). For generation of the ΔgenB4ΔgenB3 double mutant, DNA fragments flanking the *genB3* gene in the *genB4* mutant were amplified using primer pairs gen4-genB3-L1/L2 and genB4-genB3-R1/R2 (see List of Primers Used in the Supplemental Information) and inserted into vector pYH7 to yield plasmid pWHU43 (Tables S2 and S3). All plasmids were verified by sequencing.

### Targeted In-Frame Gene Deletion

To create individual in-frame deletion mutants of *genQ*, *genK*, *genB1*, *genB2*, *genB3* and *genB4*, the corresponding plasmid pYH286, pWHU1, pWHU4, pWHU2, pWHU5, and pWHU3 was introduced into the wild-type strain by conjugation from *E. coli* ET12567/pUZ8002 (MacNeil et al., 1992). Apramycin-sensitive (Apr^S^) colonies were counterselected from the initial Apr^R^ exconjugants after nonselective growth. The Apr^S^ exconjugants were confirmed by PCR amplification with appropriate checking primers and by Southern hybridization (Figures S2 and S4B). ΔgenQΔgenK was obtained by disruption of the *genK* gene in the ΔgenQ mutant using plasmid pWHU1 (Figure S2 and Tables S2 and S3). Similarly, the double-, triple-, and quadruple-in-frame deletion mutants of *genB* genes in all combinations were created by disruption of target gene(s) in appropriate mutants (Figure S4B and Table S3).

### Complementation of the ΔgenQ and ΔgenK Mutants

Complementation plasmids were prepared by cloning *genQ*, *genK* and *neo11*, respectively, into pWHU77 (a plasmid derived from pIB139 (Wilkinson et al., 2002; Del Vecchio et al., 2003) with the apramycin resistance gene replaced by a thiostrepton resistance gene) under the control of the P*ermE^∗^* promoter (Figure S2(4)D). The *genQ* and *genK* were amplified from the genomic DNA of *M. echinospora* ATCC15835 using primer pairs genQ-EP1/genQ-EP2 and genK-EP1/genK-EP2 (see List of Primers Used in the Supplemental Information), respectively. Primers neo11-EP1 and neo11-EP2 were used to amplify *neo11* from *S. fradiae* NCIMB8233. The PCR products were inserted into pWHU77 between the *Nde*I and EcoRI sites to give pWHU163 (*genQ*), pWHU165 (*neo11*), and pWHU67 (*genK*; Table S2). After sequence confirmation, these plasmids were introduced individually into ΔgenQ (pWHU163 and pWHU165) and ΔgenK (pWHU67) by conjugation. Complemented exconjugants were verified based on thiostrepton resistance and confirmed by PCR (Figures S2(4)A to ∼S2(4)C).

### Extraction and LC-ESI-HRMS Analysis of Gentamicin C Complex and Intermediates

Cultures of wild-type and mutant strains of *M. echinospora* ATCC15835 were adjusted to pH 2.0 with HCl and the acidified broth was agitated overnight on a laboratory rocker. The clarified supernatant after centrifugation was filtered through Whatman filter paper, applied to a column of DOWEX 50 WX8-200 ion-exchange resin (2 g), preconditioned with 50 ml acetonitrile followed by 50 ml MilliQ water, and equilibrated with 50 ml of 10 mM HCl. The column was washed with 30 ml of 10 mM HCl and eluted with 10 ml of 1 M NH_4_OH. The eluate was freeze-dried, redissolved in 1 ml MilliQ water, and filtered through 0.22 μm microporous membrane before subjection to LC-ESI-HRMS analysis.

### Cloning of genQ, genB1, genB2, genB3, and genB4 Genes for Expression in *E. coli*

Genes *genQ*, *genB1*, *genB2*, *genB3*, and *genB4* were amplified from the genomic DNA of *M. echinospora* ATCC15835 by PCR (see List of Primers Used in Supplemental Information). PCR was carried out using *Pfu* DNA polymerase with 25 cycles of denaturation at 94°C for 1 min, annealing at 60°C for 1 min, and extension at 72°C for 2 min plus final extension at 72°C for 10 min. The PCR products were digested with appropriate restriction enzymes, purified by gel extraction (Fermentas), and inserted into plasmid pET28a(+). The resulting constructs (Table S2) were verified by DNA sequencing.

### Overexpression and Purification of Recombinant Proteins

*E. coli* BL21(DE3) cells containing the recombinant plasmids were cultured in LB broth containing kanamycin (50 μg/ml) at 37°C to absorption at 600 nm of 0.5 to ∼0.8 and expression induced by isopropylthiogalactoside (0.1 mM) at 16°C with shaking overnight. Cells were harvested and resuspended in binding buffer (0.5 M NaCl, 20 mM Tris-HCl [pH 7.9]). The recombinant protein was released by sonication for 4 min using a 2 s on/6 s off cycle. Clarified cell lysate was passed through a column of Co^2+^-charged His-Bind resin (GE Healthcare). After washing the column with washing buffer (0.5 M NaCl, 5-60 mM imidazole, 20 mM Tris-HCl [pH 7.9]). N-His_6_-tagged recombinant proteins were eluted with elution buffer (0.5 M NaCl, 250 mM imidazole, 20 mM Tris-HCl [pH 7.9]). Imidazole in the eluted protein solutions was removed by buffer exchange using Amicon Ultracentrifugal filters (Millipore). The purified proteins were stored at −20°C in 250 mM NaCl, 10 mM Tris-HCl, pH 7.9, and 50% glycerol. The identities of the purified proteins were confirmed by SDS-PAGE, UV-Vis absorbance analysis and LC-ESI-MS (Thermo Finnigan). Protein concentrations were determined using Bradford protein dye reagent (Sigma).

### Enzyme Assays

Assay mixtures for GenQ (50 μl) contained substrate (200 μM) and purified GenQ (16 μM) in Tris-HCl buffer (50 mM, pH 8.0). Coupled assays with GenQ and GenB (B = B1, B2, B3, or B4) contained substrate (200 μM), amino donor (2 mM), GenQ (16 μM), and GenB (20 μM) in 50 μl Tris-HCl buffer (50 mM, pH 8.0). The isomerase activity assays contained substrate (C2 or C2a, 200 μM) and each one of the four GenB enzymes (20 μM), respectively, in 50 μl Tris-HCl buffer (50 mM, pH 8.0). These high concentrations of each enzyme were chosen based on previous work with the GenQ homolog Neo11 (Huang et al., 2007) where the enzyme was found to be unstable during incubations. Incubations were at 30°C overnight and quenched by addition of chloroform (100 μl) followed by vortexing and centrifugation to remove protein. The supernatants were analyzed by LC-ESI-MS.

### LC-ESI-MS Analyses

LC-ESI-HRMS analysis of extracts of wild-type and mutant strains of *M. echinospora* ATCC15835 was performed on a Thermo Electron LTQ-Orbitrap XL fitted with a Phenomenex Luna C18 column at a flow rate of 0.4 ml/min using a mobile phase of (A) 0.2% trifluoroacetic acid (TFA) in H_2_O (adjusted to pH 2.0 with NH_4_OH) and (B) 100% CH_3_CN; the gradient for separation of both gentamicin C complex and related intermediates: 0–18 min 2% B to 14% B, 18–19 min 14% B to 40% B, 19–24 min 40% B, 24–25 min 40% B to 2% B, 25–30 min 2% B. The gradient for separation of gentamicin C complex: 0–14 min 2% B to 6% B, 14–16 min 6% B to 8% B, 16–25 min 8% B-15% B, 25–26 min 15% B to 40% B, 26–34 min 40% B, 34–35 min 40% B to 2% B, 35–45 min 2% B. LC-ESI-MS analysis of assay mixtures and proteins was carried out on a Thermo Finnigan LCQ connected to an Agilent HP 1100 HPLC system. Deproteinated assay mixtures were separated on a Prodigy C18 column (Phenomenex) at a 0.4 ml/min flow rate using a mobile phase of (A) 0.3% TFA in H_2_O and (B) 0.1% TFA in CH_3_CN; 98% A for 10 min, gradient to 90% B over 15 min, then 90% B for 5 min followed by a gradient from 90% B to 2% B. MS/MS analyses were carried out in the positive ionization mode with 35% relative collision energy. Aminoglycosides were quantified by integration of areas under peaks. For proteins, a Nucleosil C4 column (250 × 2 mm, 5 μm, Macherery-Nagel) was used at a flow rate of 0.2 ml/min with the following conditions: mobile phase (A) H_2_O 0.1% TFA and (B) CH_3_CN 0.1% TFA; 35% B to 45% B over 5 min, increase the gradient of B to 75% over 20 min, 95% B for 2 min, 95% B for 7 min, followed by a gradient from 95% B to 35%B over 3 min.

## Author Contributions

F.H., P.F.L., and Y.S. conceived the experiments; J.G., C.H., X.D., and X.J. constructed and analyzed mutants; F.H. carried out in vitro analyses; J.G., F.H., C.H., F.J.L., Z.D., P.F.L., and Y.S. analyzed the results; and P.F.L. and Y.S. wrote the paper.

## Supplemental Information

Document S1. Supplemental Experimental Procedures, Figures S1–S7, and Tables S1–S3

Document S2. Article plus Supplemental Information

## References

[bib1] Aboshanab K.M.A. (2005). Genetic studies on the biosynthesis of the major aminoglycoside antibiotics: isolation, analysis and comparison of the biosynthetic gene clusters for 12 aminoglycoside antibiotics. PhD thesis.

[bib2] Bérdy J., Pauncz J.K., Vajna Z.M., Horváth G., Gyimesi J., Koczka I. (1977). Metabolites of gentamicin-producing Micromonospora species I. Isolation and identification of metabolites. J. Antibiot..

[bib3] Bockenhauer D., Hug M.J., Kleta R. (2009). Cystic fibrosis, aminoglycoside treatment and acute renal failure: the not so gentle micin. Pediatr. Nephrol..

[bib4] Clausnitzer D., Piepersberg W., Wehmeier U.F. (2011). The oxidoreductases LivQ and NeoQ are responsible for the different 6′-modifications in the aminoglycosides lividomycin and neomycin. J. Appl. Microbiol..

[bib5] Cuccarese M.F., Singh A., Amiji M., O’Doherty G.A. (2013). A novel use of gentamicin in the ROS-mediated sensitization of NCI-H460 lung cancer cells to various anticancer agents. ACS Chem. Biol..

[bib6] Del Vecchio F., Petkovic H., Kendrew S.G., Low L., Wilkinson B., Lill R., Cortés J., Rudd B.A.M., Staunton J., Leadlay P.F. (2003). Active-site residue, domain and module swaps in modular polyketide synthases. J. Ind. Microbiol. Biotechnol..

[bib7] Fan Q.Z., Huang F.L., Leadlay P.F., Spencer J.B. (2008). The neomycin biosynthetic gene cluster of *Streptomyces fradiae* NCIMB 8233: genetic and biochemical evidence for the roles of two glycosyltransferases and a deacetylase. Org. Biomol. Chem..

[bib8] Hainrichson M., Nudelman I., Baasov T. (2008). Designer aminoglycosides: the race to develop improved antibiotics and compounds for the treatment of human genetic diseases. Org. Biomol. Chem..

[bib9] Hong W.R., Yan L.B. (2012). Identification of *gntK*, a gene required for the methylation of purpurosamine C-6′ in gentamicin biosynthesis. J. Gen. Appl. Microbiol..

[bib10] Hong W.R., Ge M., Zeng Z.H., Zhu L., Luo M.Y., Shao L., Chen D.J. (2009). Molecular cloning and sequence analysis of the sisomicin biosynthetic gene cluster from *Micromonospora inyoensis*. Biotechnol. Lett..

[bib11] Houghton J.L., Green K.D., Chen W., Garneau-Tsodikova S. (2010). The future of aminoglycosides: the end or renaissance?. ChemBioChem.

[bib12] Huang F.L., Haydock S.F., Mironenko T., Spiteller D., Li Y., Spencer J.B. (2005). The neomycin biosynthetic gene cluster of *Streptomyces fradiae* NCIMB 8233: characterisation of an aminotransferase involved in the formation of 2-deoxystreptamine. Org. Biomol. Chem..

[bib13] Huang F.L., Spiteller D., Koorbanally N.A., Li Y., Llewellyn N.M., Spencer J.B. (2007). Elaboration of neosamine rings in the biosynthesis of neomycin and butirosin. ChemBioChem.

[bib14] Karki S., Kim J.Y., Park S.H., Kwon H.J. (2012). Gene inactivation study on *gntK*, a putative C-methyltransferase gene in gentamicin biosynthesis from *Micromonospora echinospora*. J. Kor. Soc. Appl. Biol. Chem..

[bib15] Kase H., Odakura Y., Nakayama K. (1982). Sagamicin and the related aminoglycosides: fermentation and biosynthesis. I. Biosynthetic studies with the blocked mutants of *Micromonospora sagamiensis*. J. Antibiot..

[bib16] Kase H., Shimura G., Iida T., Nakayama K. (1982). Biotransformation of sisomicin and verdamicin by *Micromonospora sagamiensis*. Agric. Biol. Chem..

[bib17] Kharel M.K., Basnet D.B., Lee H.C., Liou K., Woo J.S., Kim B.G., Sohng J.K. (2004). Isolation and characterization of the tobramycin biosynthetic gene cluster from *Streptomyces tenebrarius*. FEMS Microbiol. Lett..

[bib18] Kharel M.K., Basnet D.B., Lee H.C., Liou K., Moon Y.H., Kim J.J., Woo J.S., Sohng J.K. (2004). Molecular cloning and characterization of a 2-deoxystreptamine biosynthetic gene cluster in gentamicin-producing *Micromonospora echinospora* ATCC15835. Mol. Cells.

[bib19] Kharel M.K., Subba B., Basnet D.B., Woo J.S., Lee H.C., Liou K., Sohng J.K. (2004). A gene cluster for biosynthesis of kanamycin from *Streptomyces kanamyceticus*: comparison with gentamicin biosynthetic gene cluster. Arch. Biochem. Biophys..

[bib20] Kim J.Y., Suh J.W., Kang S.H., Phan T.H., Park S.H., Kwon H.J. (2008). Gene inactivation study of *gntE* reveals its role in the first step of pseudotrisaccharide modifications in gentamicin biosynthesis. Biochem. Biophys. Res. Commun..

[bib21] Kim H.J., McCarty R.M., Ogasawara Y., Liu Y.N., Mansoorabadi S.O., LeVieux J., Liu H.W. (2013). GenK-catalyzed C-6′ methylation in the biosynthesis of gentamicin: isolation and characterization of a cobalamin-dependent radical SAM enzyme. J. Am. Chem. Soc..

[bib22] Kobayashi M., Sone M., Umemura M., Nabeshima T., Nakashima T., Hellström S. (2008). Comparisons of cochleotoxicity among three gentamicin compounds following intratympanic application. Acta Otolaryngol..

[bib23] Kudo F., Eguchi T. (2009). Biosynthetic genes for aminoglycoside antibiotics. J. Antibiot..

[bib24] Kudo F., Sucipto H., Eguchi T. (2009). Enzymatic activity of a glycosyltransferase KanM2 encoded in the kanamycin biosynthetic gene cluster. J. Antibiot..

[bib25] Lee B.K., Bailey J.V., Condon R.G., Marquez J.A., Wagman G.H., Weinstein M.J. (1977). Biotransformation of sisomicin to gentamicin C2b. Antimicrob. Agents Chemother..

[bib26] Li D., Li H., Ni X., Zhang H., Xia H.Z. (2013). Construction of a gentamicin C1a-overproducing strain of *Micromonospora purpurea* by inactivation of the *gacD* gene. Microbiol. Res..

[bib27] Linde L., Kerem B. (2008). Introducing sense into nonsense in treatments of human genetic diseases. Trends Genet..

[bib28] Llewellyn N.M., Spencer J.B. (2006). Biosynthesis of 2-deoxystreptamine-containing aminoglycoside antibiotics. Nat. Prod. Rep..

[bib29] MacNeil D.J., Occi J.L., Gewain K.M., MacNeil T., Gibbons P.H., Ruby C.L., Danis S.J. (1992). Complex organization of the *Streptomyces avermitilis* genes encoding the avermectin polyketide synthase. Gene.

[bib30] Missiakas D., Georgopoulos C., Raina S. (1993). The *Escherichia coli* heat shock gene *htpY*: mutational analysis, cloning, sequencing, and transcriptional regulation. J. Bacteriol..

[bib31] Ota Y., Tamegai H., Kudo F., Kuriki H., Koike-Takeshita A., Eguchi T., Kakinuma K. (2000). Butirosin-biosynthetic gene cluster from *Bacillus circulans*. J. Antibiot..

[bib32] Park J.W., Hong J.S., Parajuli N., Jung W.S., Park S.R., Lim S.K., Sohng J.K., Yoon Y.J. (2008). Genetic dissection of the biosynthetic route to gentamicin A2 by heterologous expression of its minimal gene set. Proc. Natl. Acad. Sci. USA.

[bib33] Park J.W., Park S.R., Nepal K.K., Han A.R., Ban Y.H., Yoo Y.J., Kim E.J., Kim E.M., Kim D., Sohng J.K., Yoon Y.J. (2011). Discovery of parallel pathways of kanamycin biosynthesis allows antibiotic manipulation. Nat. Chem. Biol..

[bib34] Park S.R., Park J.W., Ban Y.H., Sohng J.K., Yoon Y.J. (2013). 2-Deoxystreptamine-containing aminoglycoside antibiotics: recent advances in the characterization and manipulation of their biosynthetic pathways. Nat. Prod. Rep..

[bib35] Reimann H., Cooper D.J., Mallams A.K., Jaret R.S., Yehaskel A., Kugelman M., Vernay H.F., Schumacher D. (1974). The structure of sisomicin, a novel unsaturated aminocyclitol antibiotic from *Micromonospora inyoensis*. J. Org. Chem..

[bib36] Sandoval R.M., Reilly J.P., Running W., Campos S.B., Santos J.R., Phillips C.L., Molitoris B.A. (2006). A non-nephrotoxic gentamicin congener that retains antimicrobial efficacy. J. Am. Soc. Nephrol..

[bib37] Shao L., Chen J.S., Wang C.X., Li J.A., Tang Y.M., Chen D.J., Liu W. (2013). Characterization of a key aminoglycoside phosphotransferase in gentamicin biosynthesis. Bioorg. Med. Chem. Lett..

[bib38] Subba B., Kharel M.K., Lee H.C., Liou K., Kim B.G., Sohng J.K. (2005). The ribostamycin biosynthetic gene cluster in *Streptomyces ribosidificus*: comparison with butirosin biosynthesis. Mol. Cells.

[bib39] Sun Y.H., He X.Y., Liang J.D., Zhou X.F., Deng Z.X. (2009). Analysis of functions in plasmid pHZ1358 influencing its genetic and structural stability in *Streptomyces lividans* 1326. Appl. Microbiol. Biotechnol..

[bib40] Testa R.T., Tilley B.C. (1975). Biotransformation, a new approach to aminoglycoside biosynthesis. I. Sisomicin. J. Antibiot..

[bib41] Testa R.T., Tilley B.C. (1976). Biotransformation, a new approach to aminoglycoside biosynthesis: II. Gentamicin. J. Antibiot..

[bib42] Thibodeaux C.J., Melançon C.E., Liu H.W. (2008). Natural-product sugar biosynthesis and enzymatic glycodiversification. Angew. Chem. Int. Ed. Engl..

[bib43] Unwin J., Standage S., Alexander D., Hosted T., Horan A.C., Wellington E.M. (2004). Gene cluster in *Micromonospora echinospora* ATCC15835 for the biosynthesis of the gentamicin C complex. J. Antibiot..

[bib44] Waksman S.A., Lechevalier H.A. (1949). Neomycin, a new antibiotic active against Streptomycin-resistant bacteria, including tuberculosis organisms. Science.

[bib45] Weinstein M.J., Luedemann G.M., Oden E.M., Wagman G.H. (1963). Gentamicin, a new broad-spectrum antibiotic complex. Antimicrob Agents Chemother (Bethesda).

[bib46] Weinstein M.J., Wagman G.H., Marquez J.A., Testa R.T., Waitz J.A. (1975). Verdamicin, a new broad spectrum aminoglycoside antibiotic. Antimicrob. Agents Chemother..

[bib47] Wilkinson C.J., Hughes-Thomas Z.A., Martin C.J., Böhm I., Mironenko T., Deacon M., Wheatcroft M., Wirtz G., Staunton J., Leadlay P.F. (2002). Increasing the efficiency of heterologous promoters in actinomycetes. J. Mol. Microbiol. Biotechnol..

[bib48] Yokoyama K., Yamamoto Y., Kudo F., Eguchi T. (2008). Involvement of two distinct N-acetylglucosaminyltransferases and a dual-function deacetylase in neomycin biosynthesis. ChemBioChem.

[bib49] Zhang Q., van der Donk W.A., Liu W. (2012). Radical-mediated enzymatic methylation: a tale of two SAMS. Acc. Chem. Res..

