# Supplementary material for: Specificity and Promiscuity at the Branch Point in Gentamicin Biosynthesis
Source: Chem Biol. 2014 May 22;21(5):608–18. doi: 10.1016/j.chembiol.2014.03.005 (PMC4039129; doi:10.1016/j.chembiol.2014.03.005)
Supplement: Document S1. Supplemental Experimental Procedures, Figures S1–S7, and Tables S1–S3 [file mmc1.pdf]

Chemistry & Biology, Volume *21*

Supplemental Information

Specificity and Promiscuity at the Branch Point in Gentamicin Biosynthesis

Junhong Guo, Fanglu Huang, Chuan Huang, Xiaobo Duan, Xinyun Jian, Finian Leeper, Zixin Deng, Peter F. Leadlay, and Yuhui Sun

SUPPLEMENTAL INFORMATION

<u>Inventory of Supplemental Information</u>

Figure S1 Sequence alignment of GenK and GenQ with their homologs in other aminoglycoside biosynthetic pathways, related to Figure 2

Figure S2 Schematic representation and confirmation of in-frame deletion of *genK* and *genQ*, and complementation of ΔgenK and ΔgenQ related to Figure 2

Figure S3 LC-HRMS analysis of production of gentamicin C complex and intermediates by the wild type, ΔgenK, ΔgenQ, and ΔgenQΔgenK mutants of *M. echinospora* ATCC15835, related to Figure 2

Figure S4 (A) Sequence alignment of GenB1, GenB2, GenB3 and GenB4 with their homolog Neo18 from neomycin biosynthetic pathway; (B) Schematic representation and confirmation of in-frame deletion of *genB1, genB2, genB3 and genB4,* related to Figure 3

Figure S5 Confirmation of C2a production in ΔgenB2 and ΔgenB2ΔgenB1 mutants, related to Figure 3

Figure S6 Cloning and characterization of GenQ, GenB1, GenB2, GenB3 and GenB4. (A) Cloning of *genQ* with alternative start codon; (B) SDS-PAGE gel of VM_GenQ and GenB; (C) UV-Vis. Spectra of VM_GenQ and FAD; (D) UV-Vis spectra of GenB, related to Figures 4, 5 and 6

Figure S7 LC-MS analysis of activity assays of GenB1, GenB2, GenB3 and GenB4, related to Figures 4 and 5

List of oligonucleotide primers used in this study, related to Experimental Procedures

Table S1 LC-HRMS analysis of gentamicin C complex and intermediates production by the wild type and *genB* mutants of *M. echinospora* ATCC15835, related to Figure 3

Table S2 Plasmids used in this study, related to Experimental Procedures

Table S3 In-frame deletion mutants and complemented strains used in this study, related to Experimental Procedures

Figure S1, related to Figure 2. Sequence alignment of GenK (A) with homologous radical SAM-dependent methyltransferases and of GenQ (B) with homologous oxidoreductases from other aminoglycoside biosynthetic pathways

Black letters in gray boxes represent amino acid residues identical in all species, while white letters in gray boxes represent similar amino acid residues. The red box in **A** indicates the consensus CX_3CX_2C binding motif for the FeS cluster.

A

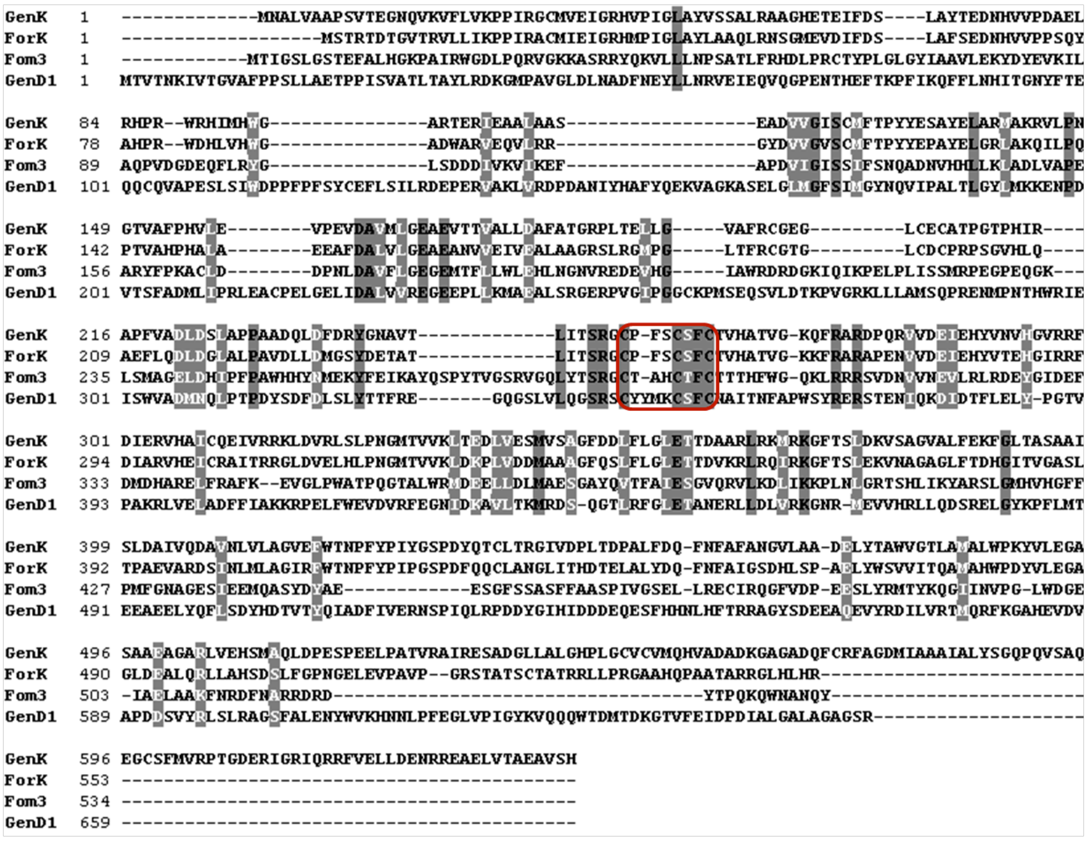

ForK (accession no. CAF31547.1): from *Micromonospora olivasterospora*;

Fom3 (accession no. ACG70826.1): from *Streptomyces fradiae*;

GenD1 (accession no. CAF31434.1): from *Micromonospora echinospora*.

B

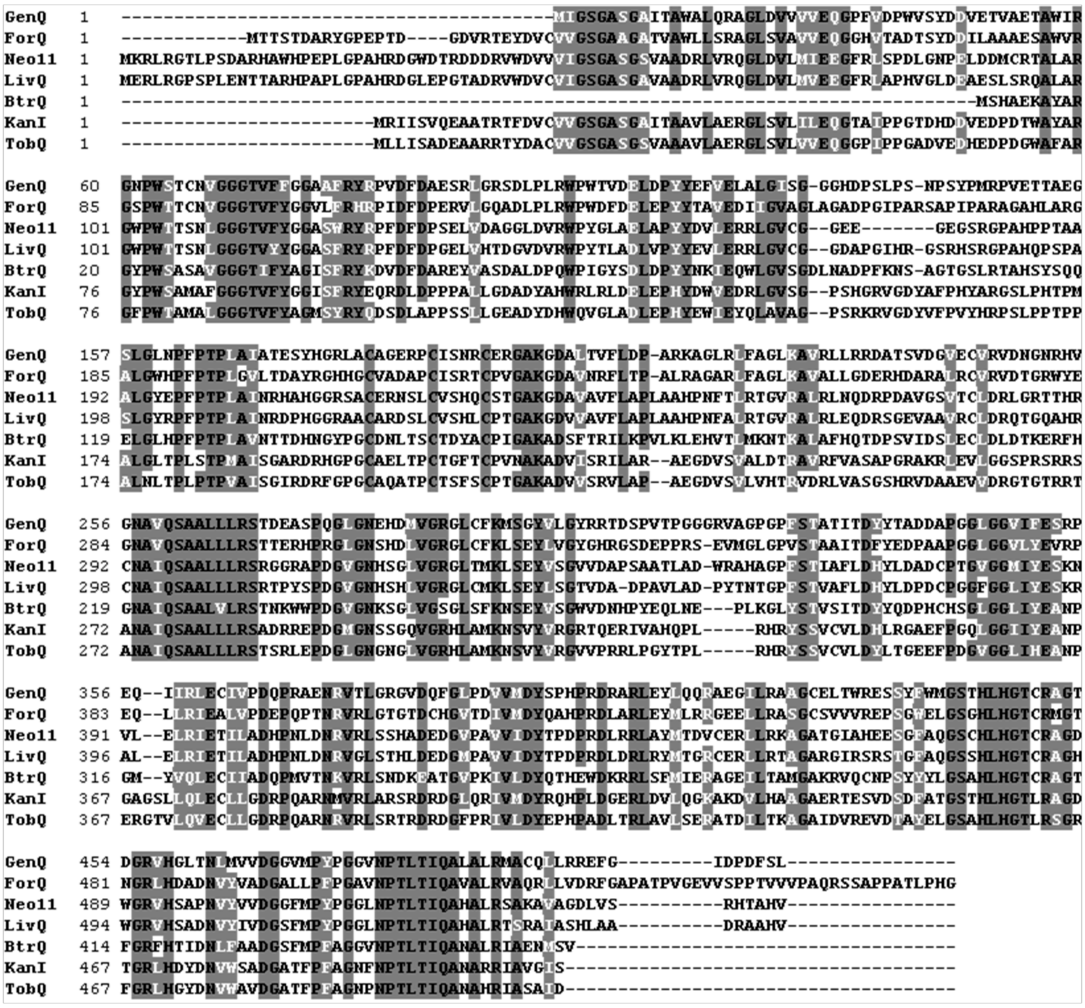

Neo11 (accession no. CAH58694.1): from *S. fradiae*;

BtrQ (accession no. BAE07079.1): from *Bacillus circulans*;

LivQ (accession no. CAG38701.1): from *S. lividus*;

KanI (accession no. CAF60534.1): from *S. kanamyceticus*;

TobQ (accession no. CAH18553.1): from *Streptoalloteichus tenebrarius*;

ForQ (accession no. CAF31536.1): from *M. olivasterospora*.

Figure S2, related to Figure 2. In-frame deletion of *genK* and *genQ* in *M. echinospora* ATCC15835*,* and complementation of ΔgenK and ΔgenQ
(A) Schematic representation of the in-frame deletions; (B) Confirmation by Southern blot; (C) Confirmation by PCR. The arrows indicate the expected size of the PCR fragments in the wild type and mutants. The dash arrows show the location of PCR primers for check mutants.

(1) ΔgenK

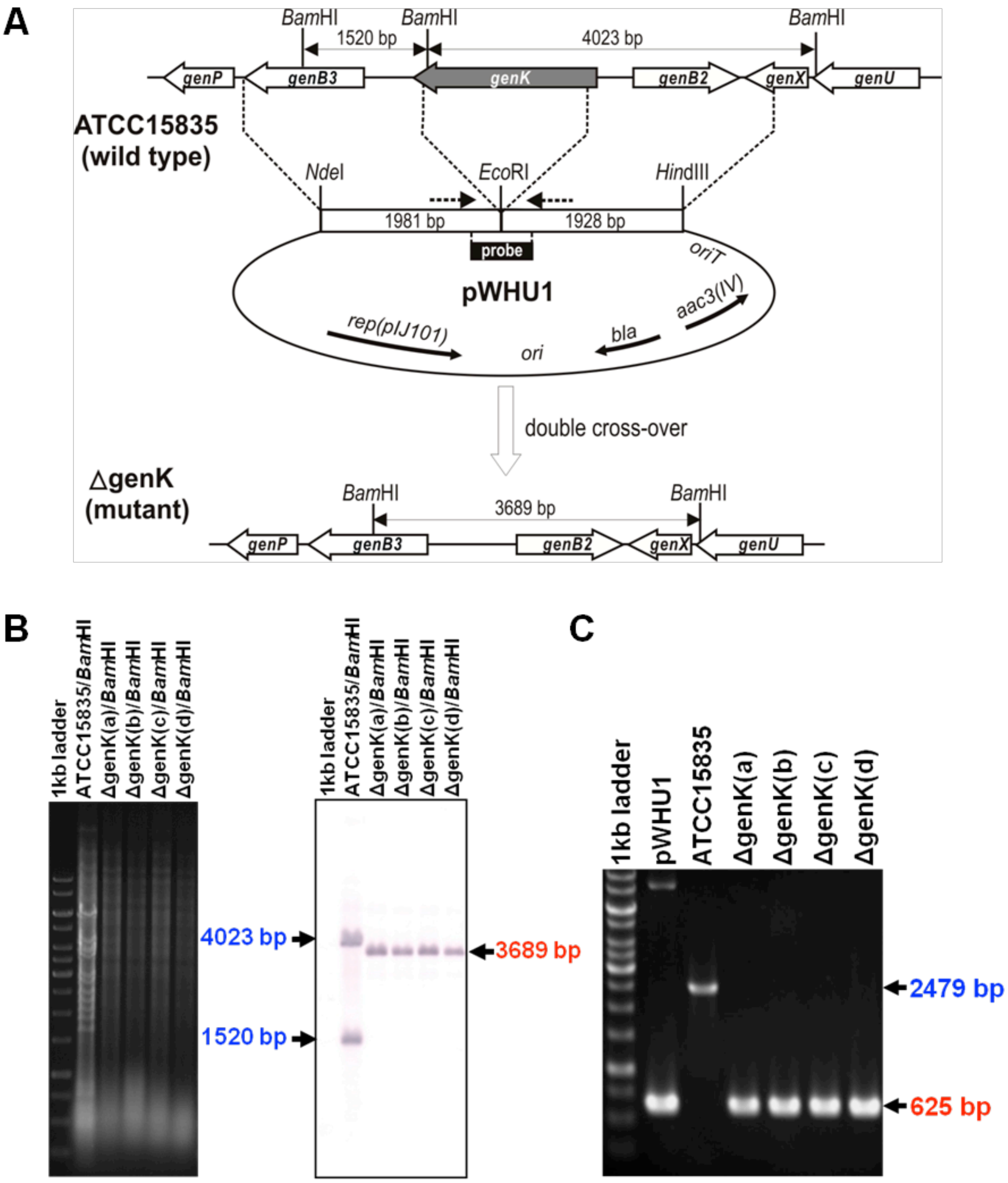

(2) ΔgenQ

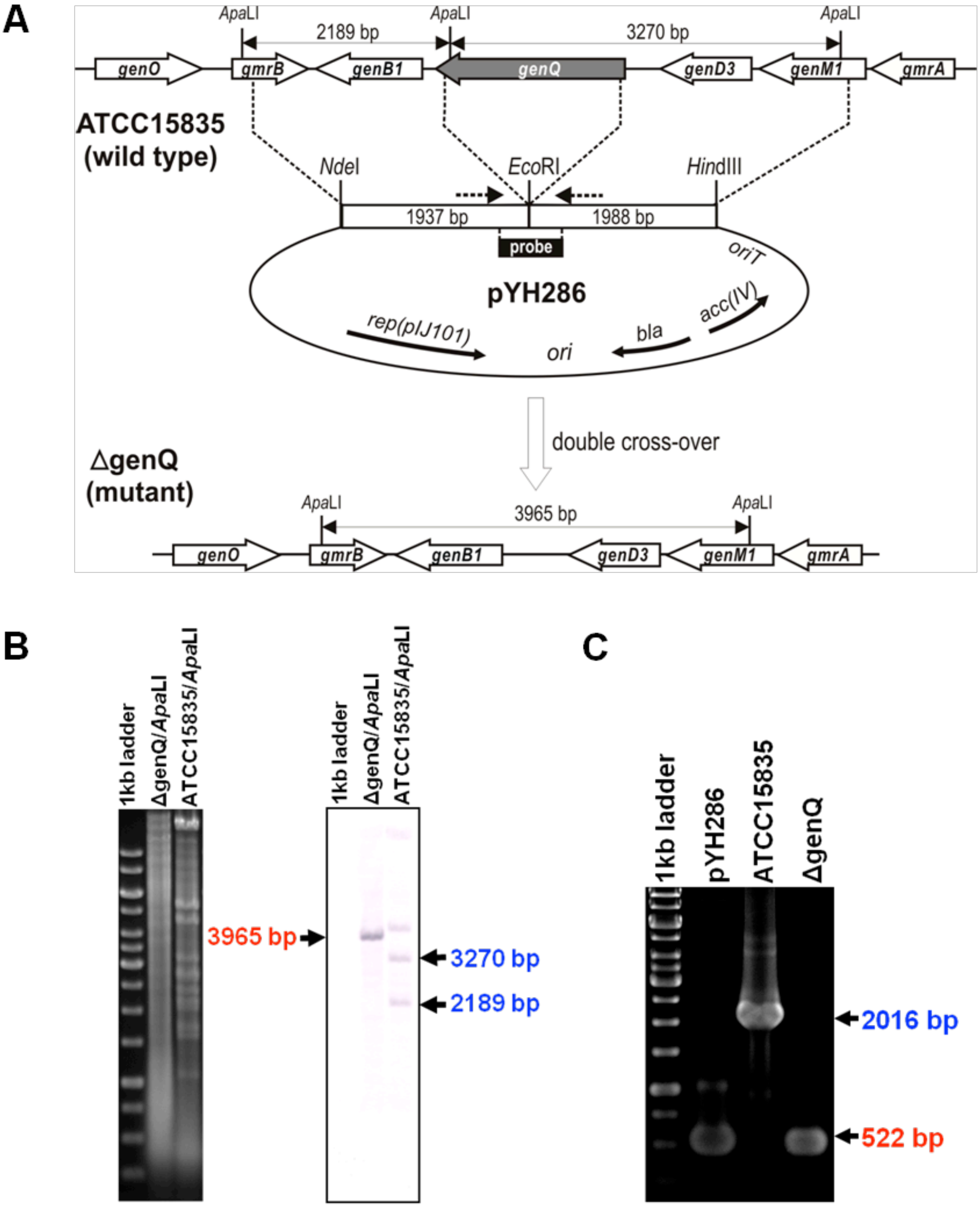
A
ApaLI
ApaLI
ApaLI
2189 bp
3270 bp
genO
gmrB
genB1
genQ
genD3
genM1
gmrA
ATCC15835
(wild type)
NdeI
EcoRI
HindIII
1937 bp
1988 bp
probe
oriT
pYH286
acc(IV)
rep(pIJ101)
bla
ori
double cross-over
ΔgenQ
(mutant)
ApaLI
ApaLI
3965 bp
genO
gmrB
genB1
genD3
genM1
gmrA
B
1kb ladder
ΔgenQ/ApaLI
ATCC15835/ApaLI
3965 bp
1kb ladder
ΔgenQ/ApaLI
ATCC15835/ApaLI
3270 bp
2189 bp
C
1kb ladder
pYH286
ATCC15835
ΔgenQ
2016 bp
522 bp

(3) ΔgenQΔgenK

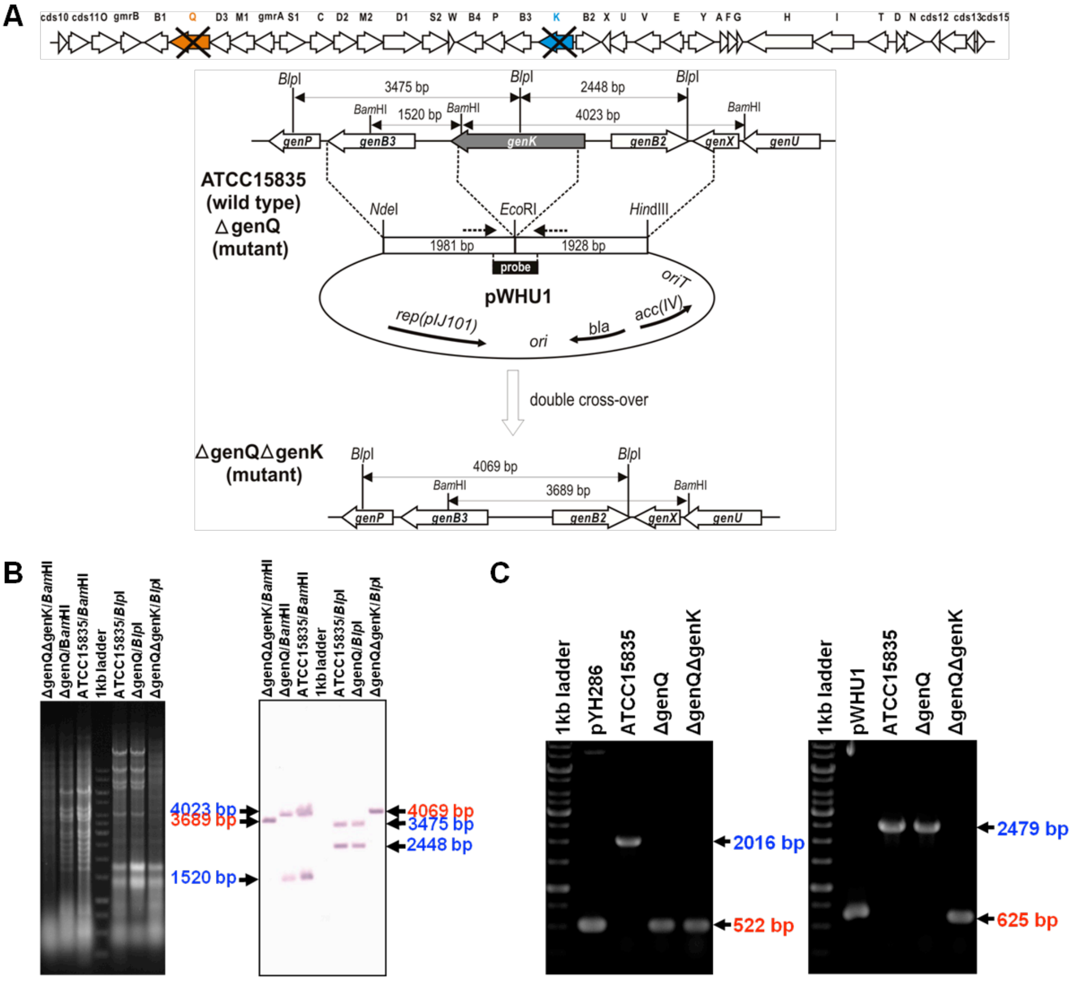

(4) Complementation of ΔgenK and ΔgenQ

Confirmation of complementation strains (A) ΔgenK::*genK*, (B) ΔgenQ::*genQ* and (C) ΔgenQ::*neo11* by PCR. The arrows indicate the expected size of the PCR fragments in the wild type and mutants. (D) Map of genetic complementation vector. The dash arrows indicate the location of PCR primers for check complementation strains.

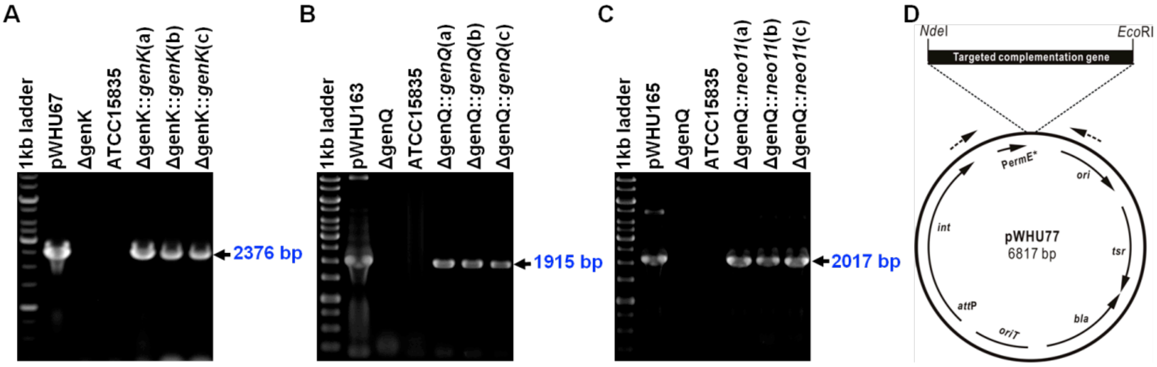

Figure S3, related to Figure 2. LC-HRMS analysis of production of gentamicin C complex and intermediates by the wild type, ΔgenK, ΔgenQ, and ΔgenQΔgenK mutants of *M. echinospora* ATCC15835

(A) Schematic representation of the in-frame deletion of *genQ* and *genK*. Deleted genes are shown in dotted arrows. (B) Production of gentamicin C complex and intermediates analyzed by LC-ESI-HRMS and bioassay using *Bacillus pumilus* as indicator strain. -: no production, +: production, ++: increased by about 10-fold compared with wild type, +++: increased by about 100-fold compared with wild type.

A

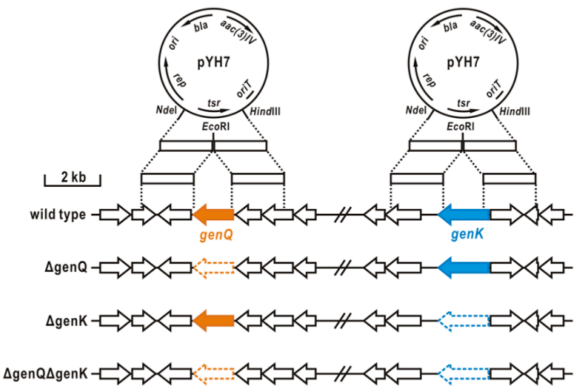

B

Strain	Production									Bioassy
	C1a	C2	C2a	C2b	C1	X2	JI-20A	G418	JI-20B	
wild type	+	+	+	+	+	+	+	+	+	
ΔgenQ	−	−	−	−	−	+	−	+++	−	
ΔgenK	++	−	−	++	−	++	++	−	−	
ΔgenQΔgenK	−	−	−	−	−	+++	−	−	−	

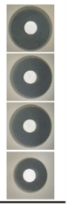

Figure S4, related to Figure 3. (A) Sequence alignment of GenB1, GenB2, GenB3 and GenB4 with their homolog Neo18 from the neomycin biosynthetic pathway. The red box in (A) indicates the putative catalytic lysine; (B) Schematic representation and confirmation by Southern blot of in-frame deletion of *genB1, genB2, genB3 and genB4.* (1) ~ (4): Single mutants; (5) ~ (10): Double mutants; (11) ~ (14): Triple mutants; (15): Quadruple mutant.

A

```
Neo18     ----------------------------------------------MTKNS---SLLAEF 11
GenB1     ----------------------------------------------MTIDIGAGKLLAQE 14
GenB2     ------------------------------------------------------MIIANA 6
GenB3     MAVADHRSSEPSWRAGRTARRRSRWHSWARVKSAREGSQDMDSANLTNRGLVERARRVTA 60
GenB4     ---------------------------------------------MNYRELIERARRTTA 15
                                                                   .

Neo18     PTCPRDEKDRPRVFTAASGAWLTDESGFRWIDFDNARGSILLGHGDPVVAEAVARAAT-- 69
GenB1     PTCPRDADGRPRVFVEGSGAYLTDPDGRRWIDFDNARGSVVLGHGDEEVAEAIARAAR-- 72
GenB2     DGCTPYEVARGVTIVRGEGAYVYDAEGRGLIDLSNSFGSVMLGHQDPVVTEAVLKTVR-- 64
GenB3     AENYDIGTRFSAMIQSGEGAWLTDVEGNRYVDLTASSGTIILGHRNQAVTEAITRQIRDF 120
GenB4     AEEYDISGRYPSVIAHAEGAWMTDLSGNRYVDLTGADAAVILGYRHPAVNEAITRQIRDY 75
                       :  ..**:: * .*   :*:  : .:::**: .  * **: :

Neo18     GADGTATGWSRRVDAVLERLHALCGGEVVGLFRSGTAAVRAAVLAVREATGRPLLLSAGY 129
GenB1     GRSGVGTAWSPVLDSLLGQLQEVCGGDVVGLYRTGTAALRSVTCAVRDARDRSIVLSSGY 132
GenB2     SGVPAAASLDLQNHLAEQIAGDLPGDQRVAFFKTGTAATRAAASAARQVTGKRLIASCGY 124
GenB3     GTAFASTLSVPRVELAERLCERYECAEKVVFHKTGSEGTAMAARLARAATGRELILSCGY 180
GenB4     GTTFASTLSVPRVELAERMCERYECAEKVVFHKTGTEGTAMAVRLARAATGRELVLSSGY 135
          .   ..:      .            : * :.::*: .   ..  .* . .: :: *.**

Neo18     HGYDPMWYPSEAPLEPNADGVVDFFFDLGLLRELLRA-PERVAAVVVSPDHMHLSPGWYR 188
GenB1     HGYDPMWH-CDEPFTPNQHGIVEFLFDLDVLAEWLSR-PEQVAAVVISPDHMHLGERWYT 190
GenB2     HGYDLMWE-FTPPGQPNSEDVLHCYHLPELIDQVLDKHAHELAAVIIAPDYIHVSPEYIA 183
GenB3     HGWHEWQLAGETFGYQQTTGVVGFGYNEKALAKMLEAFGNEVAGVLISPELLYFDVEFYQ 240
GenB4     HGWHEWQMAGEEFGYQQSTGVVGFGYNEKALAKMLEAFGEQVAGVIVSPEVLYFDLDHYR 195
          **:.            :  .::   .    : : *    ..:*.*:::*: ::..

Neo18     ELRRLCSAAGVVLVADEVKVGLRYAPGLSTAE-LLAPDVWVVAKGMANGHAVSAVGGSRR 247
GenB1     EFTRLTKEADVPVIADEVKVGLRYRAGLSTP--LLDPAVWIVAKCLANGSPVAAVGGDAH 248
GenB2     DLFERCERVGVVTIADEVKHGYRLRQGASVTEASVVADMYTYAKGISNGWPLSCVAGDER 243
GenB3     RMYALCARYDVPFMMDEVYTGFRAGP-KGVHGLGVPADVVVVSKGLANGHSLAAVMGRRD 299
GenB4     RMSALCARYDVPFMLDEVYTGFRAGP-KGVHGLGVPADVVVLGKGLANGHSLAAVMGRRD 254
           :       .*  : ***  * *     ..    : . :   .* ::** .::.* *

Neo18     LLKP--LKEVSFTSFFEPTILAAADAALARVATGEPQRAVREAGDRFLRHARKALDDASL 305
GenB1     LLAA--LEDVSFTSYFEPTAMAAATTTLRRMATGEPQQAIRAAGDRFIAHTRAAFANAGV 306
GenB2     FLKP--LAEFVSTLTFEAPSFAAASATLDRLAELDVQAQLAIDGARFVSEAAKMISTRDL 301
GenB3     IIDAYDVSGIQGTYTREVPPMAAAMAVLDVLDTPGVYEHAEAMGRRLADGMREILTGEGI 359
GenB4     IIDAYDVSGIQGTYTREVPPMAAALAVFEVLDTPGVYEHAEAMGRRLADGMREILTGEGI 314
          :: .  :  .  *   * . :*** :.:  :            * *:       :   .:

Neo18     PVEIAGDGTFFQFVPATEELEEALYGAANAEGLLFYAGDNQGVSAAFDEAVLGEAERRFA 365
GenB1     PIDLAGNGNLFQFVCADDEVADAFHAAAAAEGLLFFEGDNQTPSAAFTDEVVEDACGRID 366
GenB2     PIEMAGTGAAFQFVCA-EEVEEVLLPHALAEGLILEPSDQQYPSACFRGEVVDDALERLD 360
GenB3     PNWVGGPALMFDTVLPNDDLGWEIYKTAHDFGVYFEDSGTQLVTTAFDEAAVDHALTAFR 419
GenB4     PNWVGGPALMFDVVLPNDDLGWEIYKTAHDFGVYFEDSGTQLVTAAFDEAAVDHALTAFR 374
          *  :.* .  *: * . :::   :   *   *: :  .. *  ::.*   .: .*   :

Neo18     RVCERLAPY---AGGEPVGDAARYRVAWNVMDGLRQAPRDREETTGLLARLLDD------ 416
GenB1     RVSAALTGR---FTDRELTEESWYASAWGAMDGLADRPRTREETTAIVERLWED------ 417
GenB2     RALTTMAAARPDLVGREVTQLDRVNAAFCQMDGLPGRPDGWSLDQCVEYVTAQL------ 414
GenB3     KATRQVIADRPDIAPTSGGELTEERKLDFAEEAFGGLLRDDERTNALIDETIEKVVNRDR 479
GenB4     KATRQVVADRPDIAPTSGGELTEERKLDFAEEAFGGLLRDDERTNALIDETIEKVVNRDR 434
          :.   :             :           :.:       .    :     :

Neo18     -----------
GenB1     -----------
GenB2     -----------
GenB3     SIKPVLIPAQN 490
GenB4     SIKPVLFPAQN 445
```

B

(1) ΔgenB1

The arrows indicate the expected size of the fragments from the wild type and mutant chromosomal DNA, respectively, hybridized after digestion with *Age*I with a 581 bp probe (PCR-amplified from pWHU4 using oligonucleotide primers genB1-CK1 and CK2).

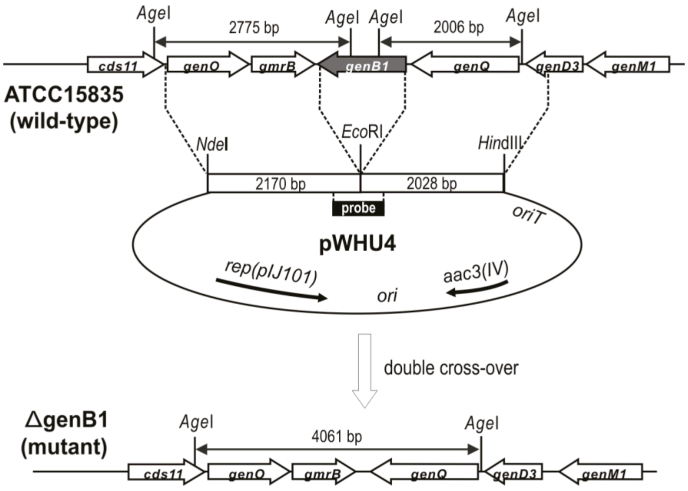

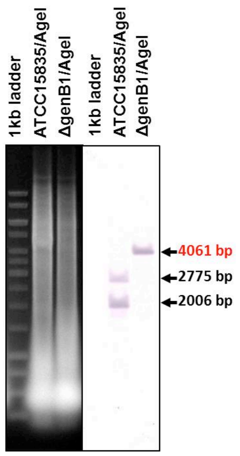

(2) ΔgenB2

The arrows indicate the expected size of the fragments from the wild type and mutant chromosomal DNA, respectively, hybridized after digestion with *Nsp*I with a 587 bp probe (PCR-amplified from pWHU2 using oligonucleotide primers genB2-CK1 and CK2).

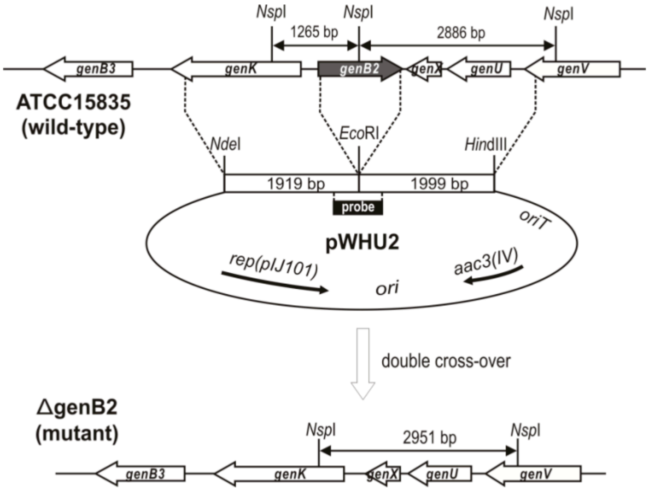

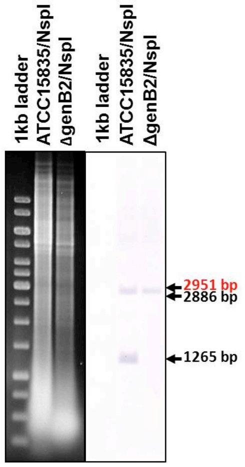

(3) ΔgenB3

The arrows indicate the expected size of the fragments from the wild type and mutant chromosomal DNA, respectively, hybridized after digestion with *Nsp*I with a 658 bp probe (PCR-amplified from pWHU5 using oligonucleotide primers genB3-CK1 and CK2).

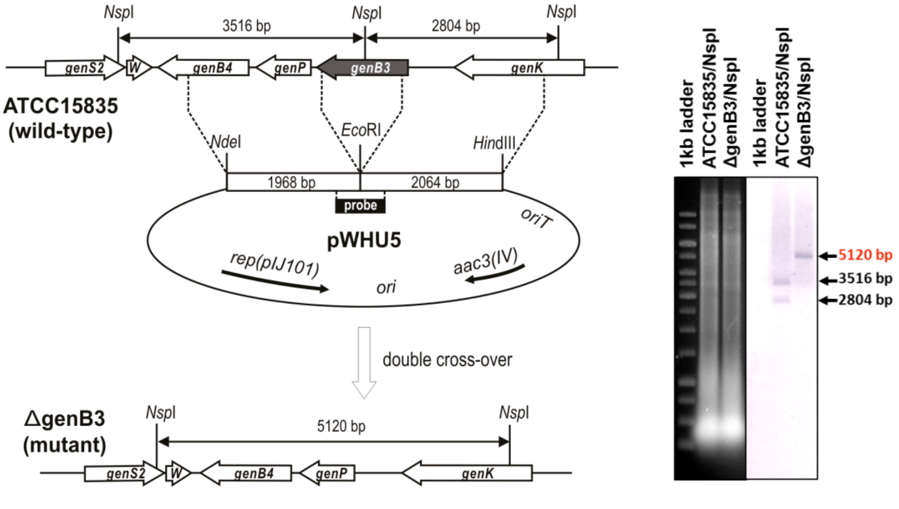

(4) ΔgenB4

The arrows indicate the expected size of the fragments from the wild type and mutant chromosomal DNA, respectively, hybridized after digestion with *Sac*II with a 595 bp probe (PCR-amplified from pWHU3 using oligonucleotide primers genB4-CK1 and CK2).

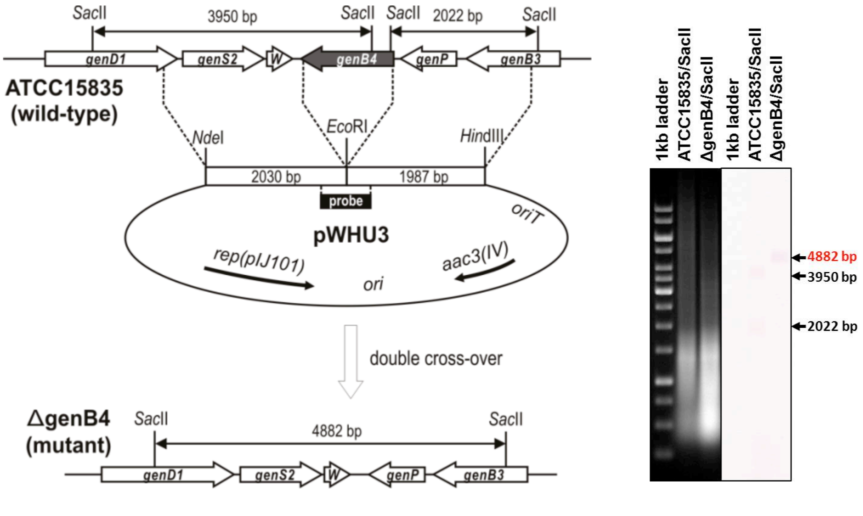

(5) ΔgenB2ΔgenB1

The arrows indicate the expected size of the fragments from the wild type and mutants chromosomal DNA, respectively, hybridized after digestion with *Age*I with a 581 bp probe (PCR-amplified from pWHU4 using oligonucleotide primers genB1-CK1 and CK2).

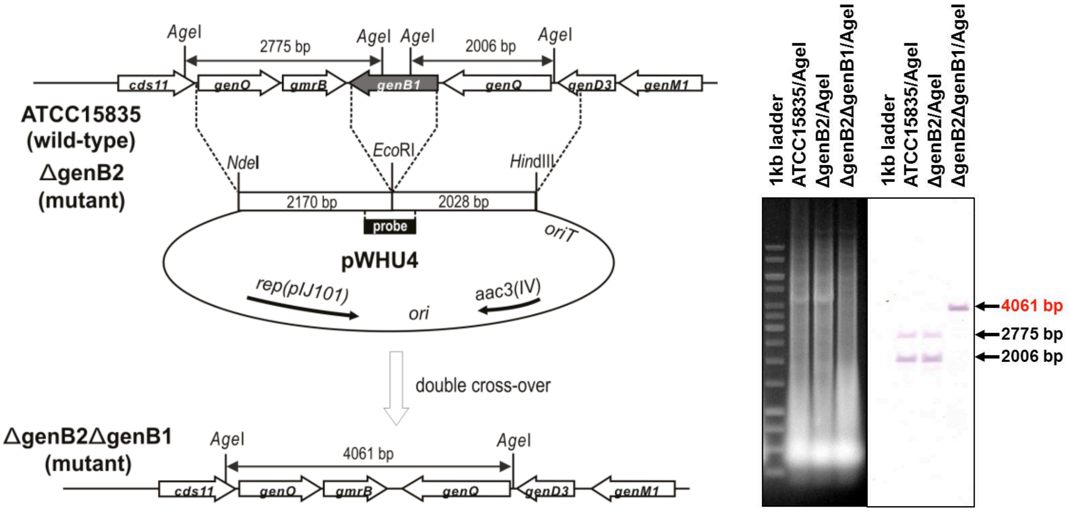

(6) ΔgenB1ΔgenB3

The arrows indicate the expected size of the fragments from the wild type and mutants chromosomal DNA, respectively, hybridized after digestion with *Psh*AI to a 658 bp probe (PCR-amplified from pWHU5 using oligonucleotide primers genB3-CK1 and CK2).

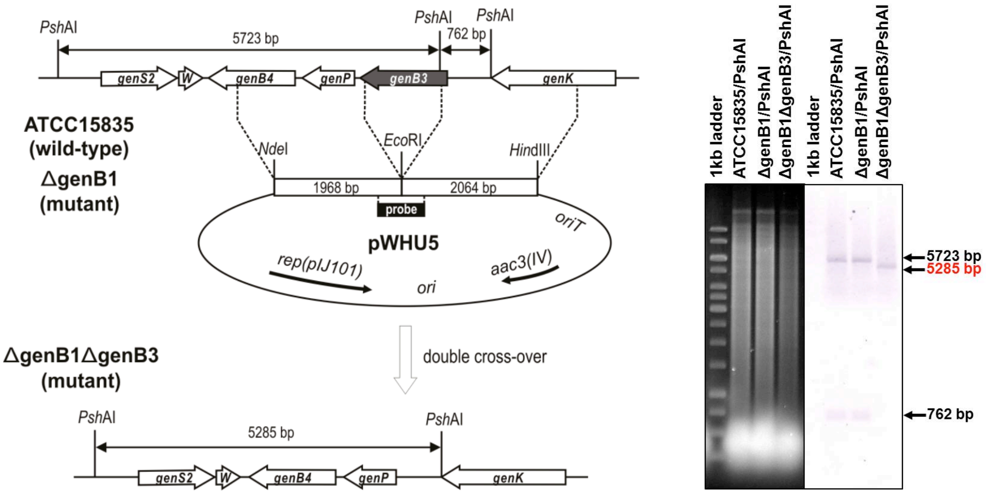

(7) ΔgenB4ΔgenB3

The arrows indicate the expected size of the fragments from the wild type and mutants chromosomal DNA, respectively, hybridized after digestion with *Sac*II with a 741 bp probe (PCR-amplified from pWHU43 using oligonucleotide primers genB4-genB3-CK1 and CK2).

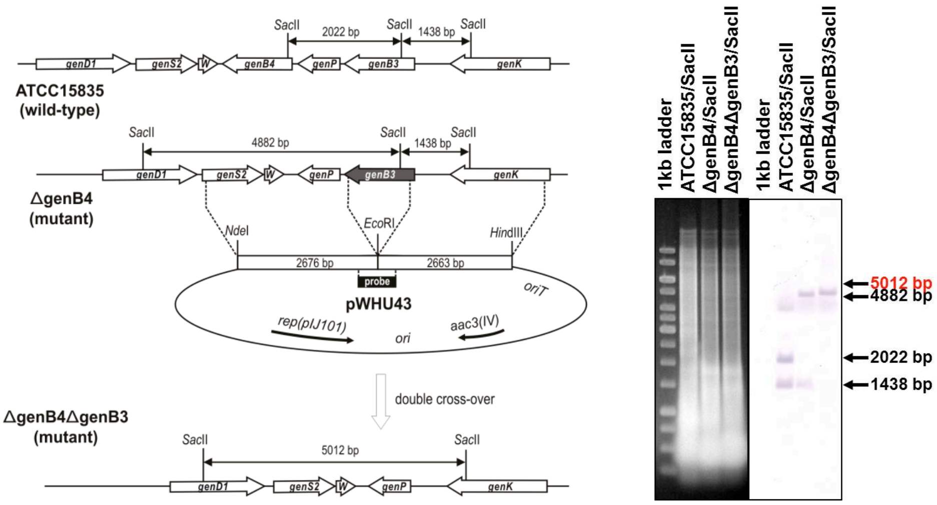

(8) ΔgenB2ΔgenB3

The arrows indicate the expected size of the fragments from the wild type and mutants chromosomal DNA, respectively, hybridized after digestion with *Psh*AI with a 658 bp probe (PCR-amplified from pWHU5 using oligonucleotide primers genB3-CK1 and CK2).

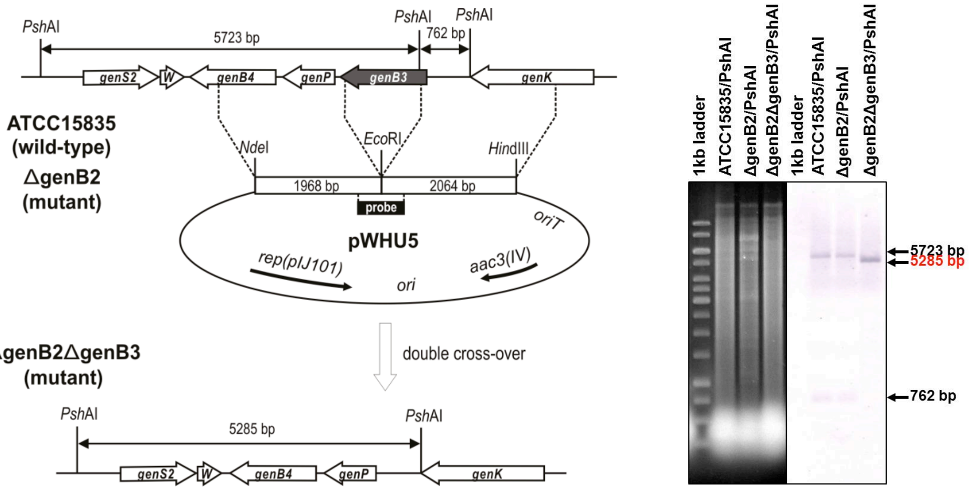

(9) ΔgenB2ΔgenB4

The arrows indicate the expected size of the fragments from the wild type and mutants chromosomal DNA, respectively, hybridized after digestion with *Sac*II with a 595 bp probe (PCR-amplified from pWHU3 using oligonucleotide primers genB4-CK1 and CK2).

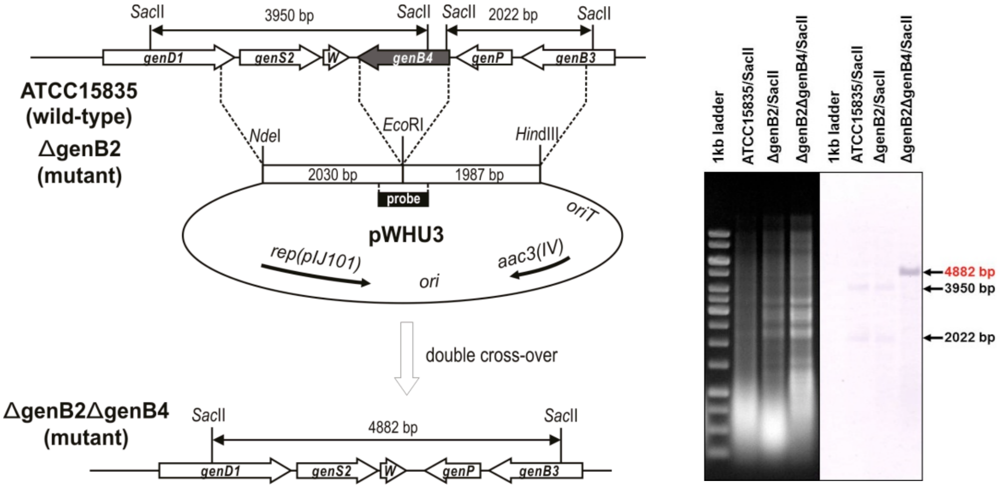

(10) ΔgenB4ΔgenB1

The arrows indicate the expected size of the fragments from the wild type and mutants chromosomal DNA, respectively, hybridized after digestion with *Age*I with a 581 bp probe (PCR-amplified from pWHU4 using oligonucleotide primers genB1-CK1 and CK2) respectively,.

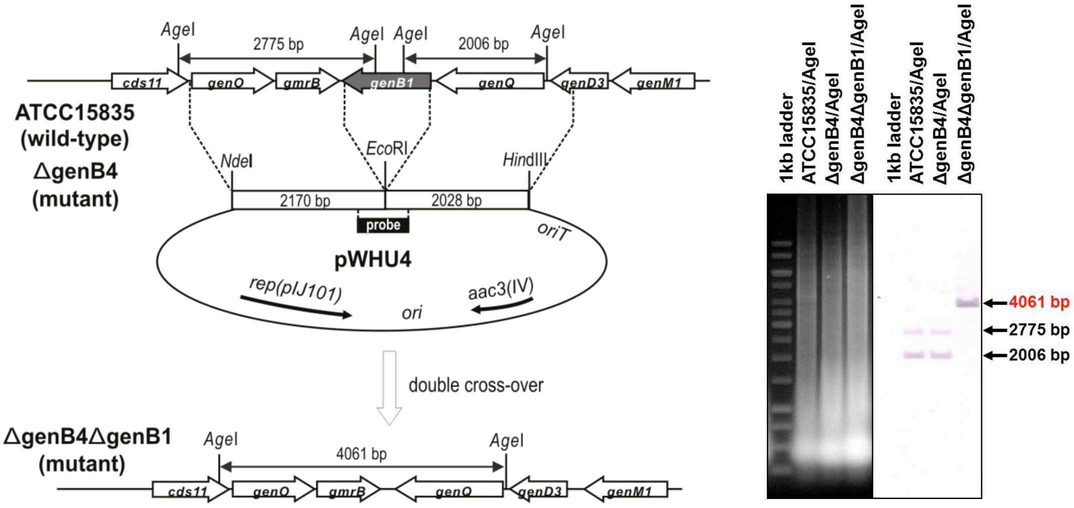

(11) ΔgenB4ΔgenB1ΔgenB2

The arrows indicate the expected size of the fragments from the wild type and mutant schromosomal DNA, respectively, hybridized after digestion with *Pvu*I with a 587 bp probe (PCR-amplified from pWHU2 using oligonucleotide primers genB2-CK1 and CK2).

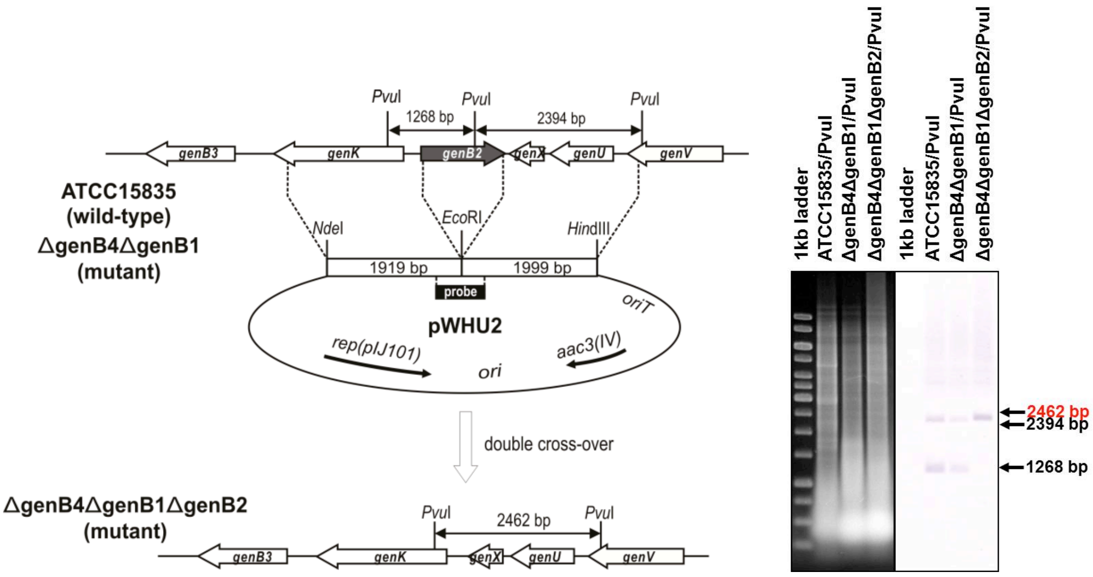

(12) ΔgenB4ΔgenB1ΔgenB3

The arrows indicate the expected size of the fragments from the wild type and mutants chromosomal DNA, respectively, hybridized after digestion with *Sac*II with a 741 bp probe (PCR-amplified from pWHU43 using oligonucleotide primers genB4-genB3-CK1 and CK2).

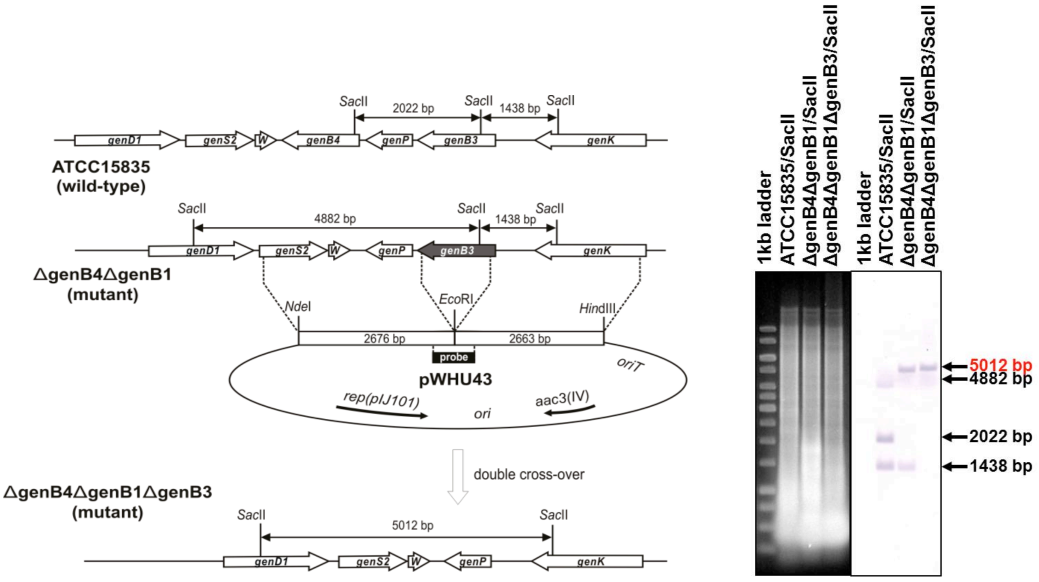

(13) ΔgenB2ΔgenB3ΔgenB1

The arrows indicate the expected size of the fragments from the wild type and mutants chromosomal DNA, respectively, hybridized after digestion with *Age*I with a 581 bp probe (PCR-amplified from pWHU4 using oligonucleotide primers genB1-CK1 and CK2).

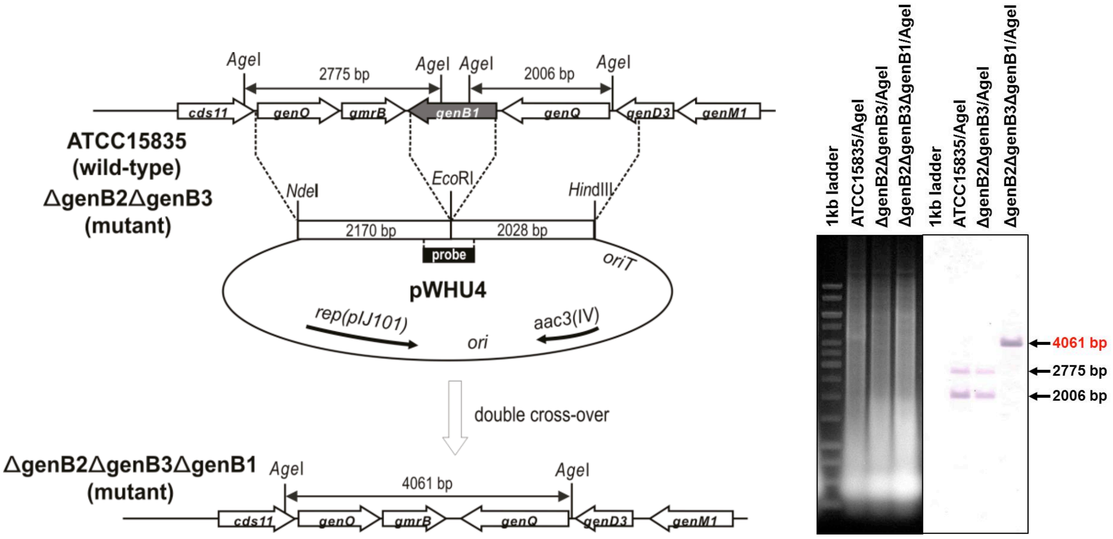

(14) ΔgenB2ΔgenB4ΔgenB3

The arrows indicate the expected size of the fragments from the wild type and mutants chromosomal DNA, respectively, hybridized after digestion with *Sac*II with a 741 bp probe (PCR-amplified from pWHU43 using oligonucleotide primers genB4-genB3-CK1 and CK2).

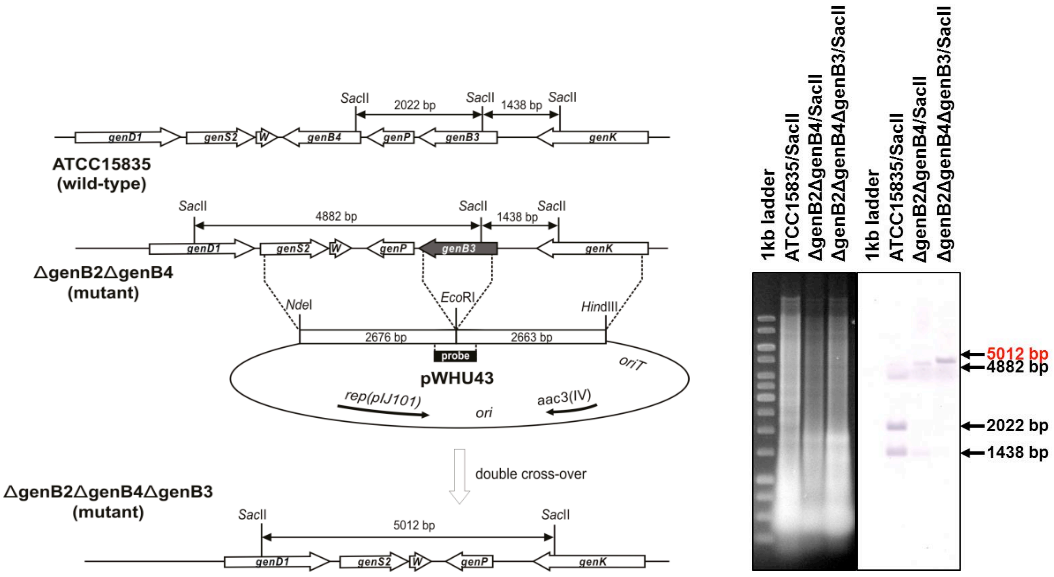

(15) ΔgenB4ΔgenB1ΔgenB3ΔgenB2

The arrows indicate the expected size of the fragments from the wild type and mutants chromosomal DNA, respectively, hybridized after digestion with *Pvu*I with a 587 bp probe (PCR-amplified from pWHU2 using oligonucleotide primers genB2-CK1 and CK2).

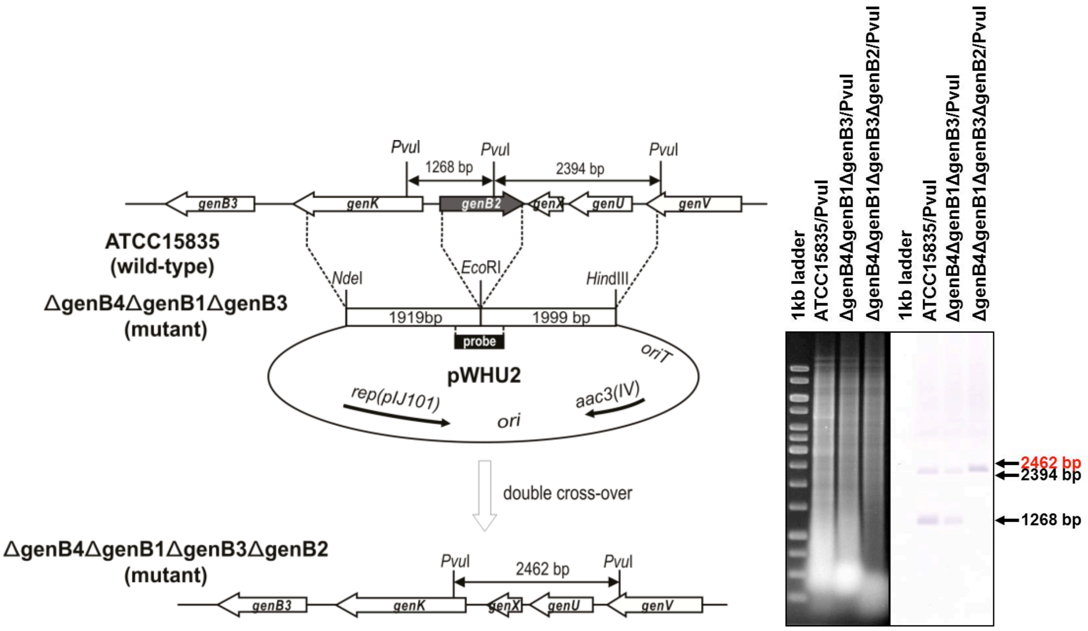

Figure S5, related to Figure 3. Confirmation of C2a production in ΔgenB2 and ΔgenB2ΔgenB1 mutants

(A) Comparison of product mixture from strain ΔgenB2 with gentamicin C2 standard; (B) Comparison of product mixture from strain ΔgenB2ΔgenB1 with gentamicin C2 standard; (C) Comparison of product mixture from strain ΔgenB2 with gentamicin C2a standard; (B) Comparison of product mixture from strain ΔgenB2ΔgenB1 with gentamicin C2a standard; (E) Comparison of product mixture from strain ΔgenB2 with gentamicin C1 standard; (F) Comparison of product mixture from strain ΔgenB2ΔgenB1 with gentamicin C1 standard.

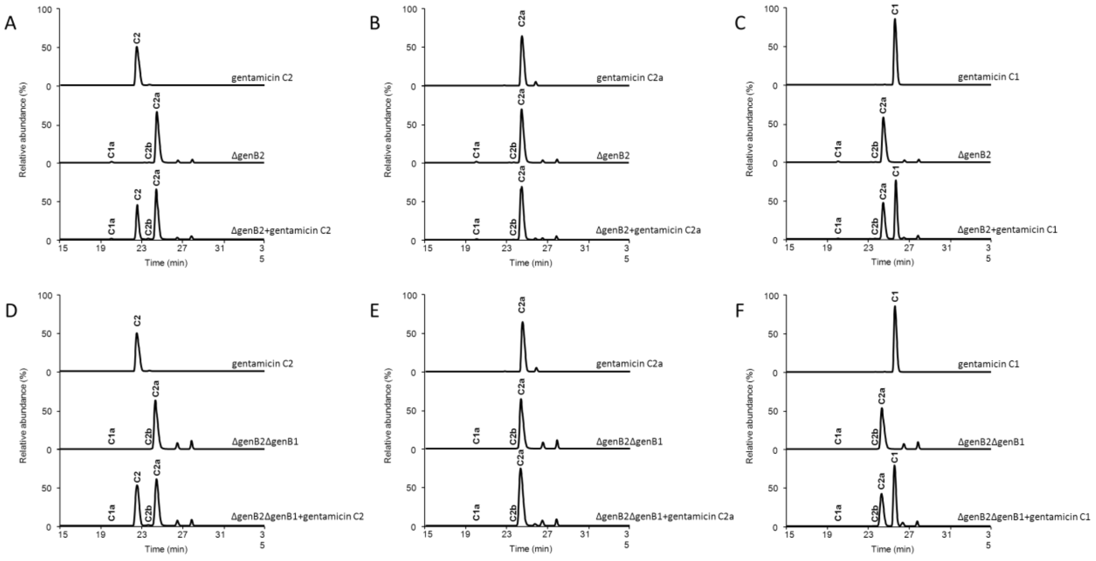

Figure S6, related to Figures 4, 5 and 6. Cloning and characterization of GenQ, GenB1, GenB2, GenB3 and GenB4

(A) Cloning *genQ* with alternative start codons. Positions of the original start codon of *genQ* and the new start codons in LM_*genQ* and VM_*genQ* are indicated in red boxes; (B) SDS-PAGE gel of VM_GenQ, GenB1, GenB2, genB3 and GenB4; (C) UV-Vis spectra of VM_GenQ and FAD standard; (D) UV-Vis spectra of GenB proteins.

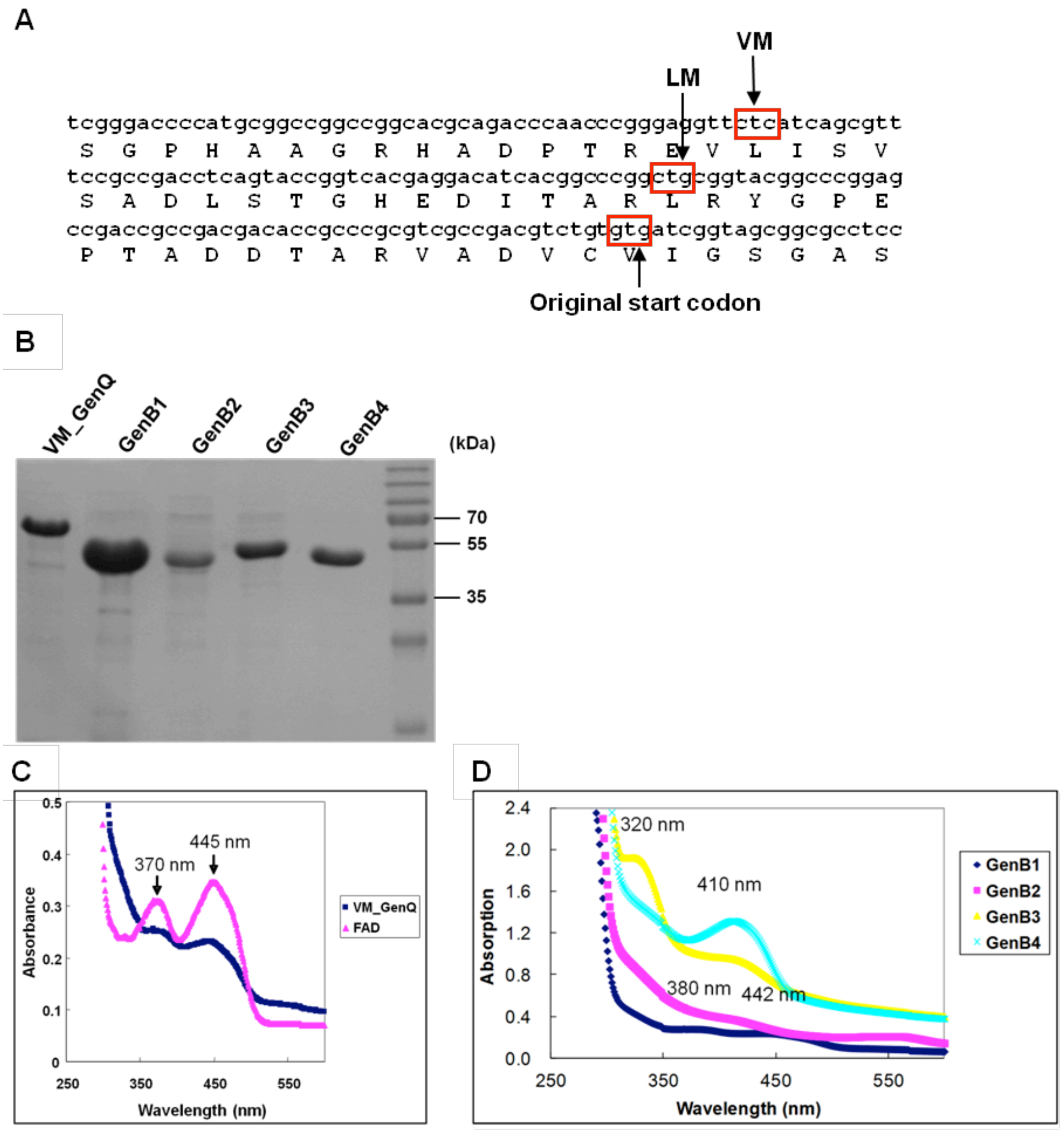

Figure S7, related to Figures 4 and 5. LC-MS analysis of activity assays of GenB1, GenB2, GenB3 and GenB4 (GenB)

(A) Test of different amino donors on the conversion of G418 to JI-20B (i - iv) and of gentamicin X2 to JI-20A(v - viii) catalysed by VM_GenQ coupled with GenB1, GenB2, GenB3, and GenB4 by LC-MS analysis. The percentages of G418, gentamicin X2, 6'-DOG, 6'-DOX, JI-20B and JI-20A present after 16 h of incubation, based on the abundance of corresponding ions detected, are shown as stacked plots. (B) LC-MS analysis of isomerase activity of GenB enzymes on gentamicin C2 and C2a. Selective ion monitoring $[M+H]^+$ (*m*/*z* 464) and $[M+Na]^+$ (*m*/*z* 486) ions of C2 and C2a.

A

i

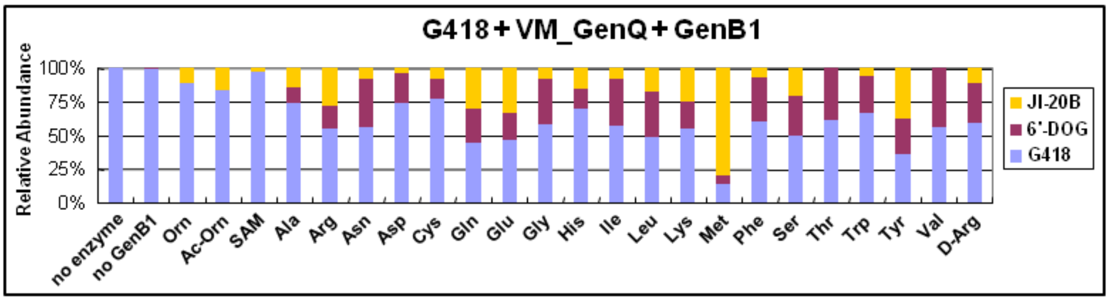

ii

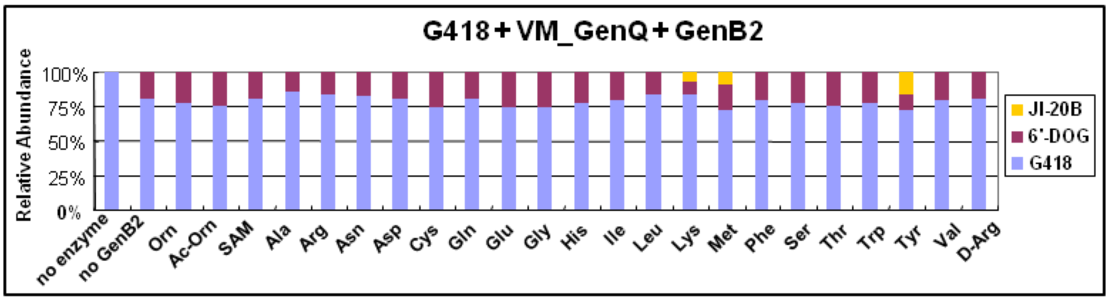

iii

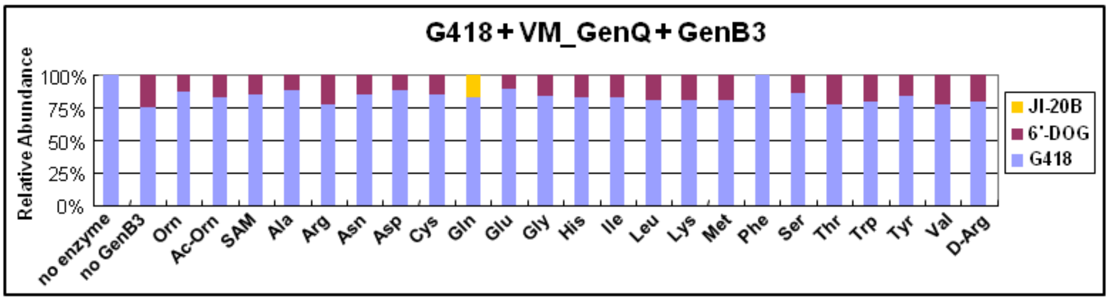

iv

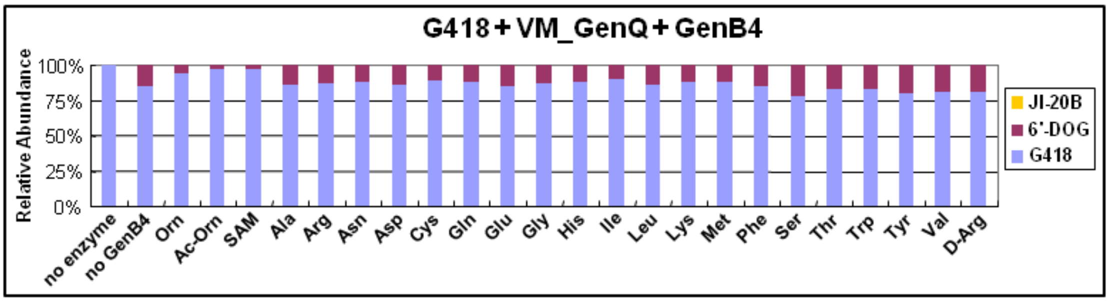

v

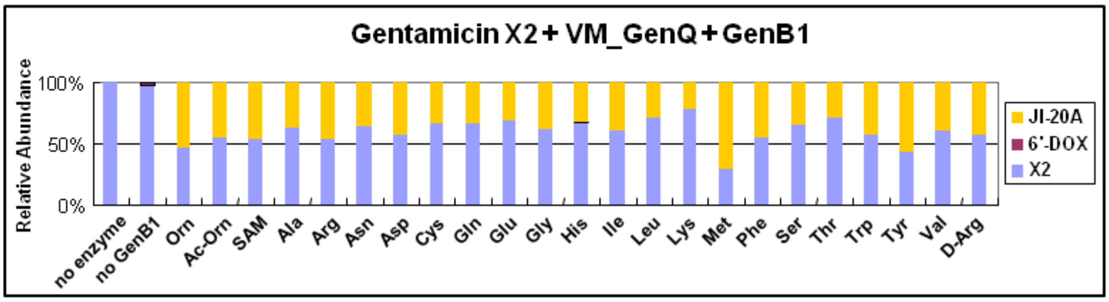

vi

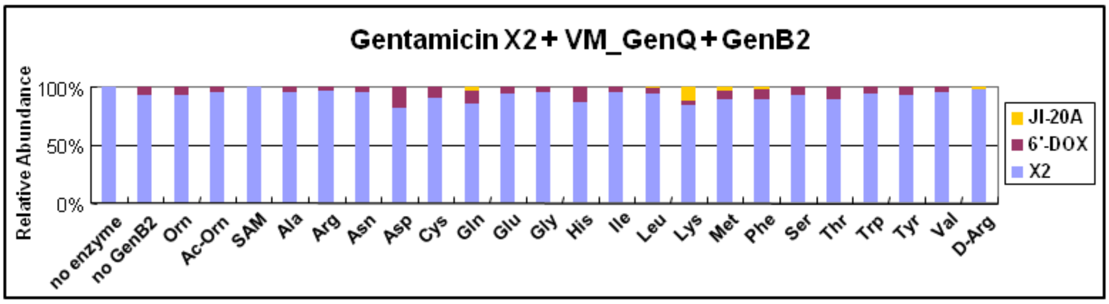

vii

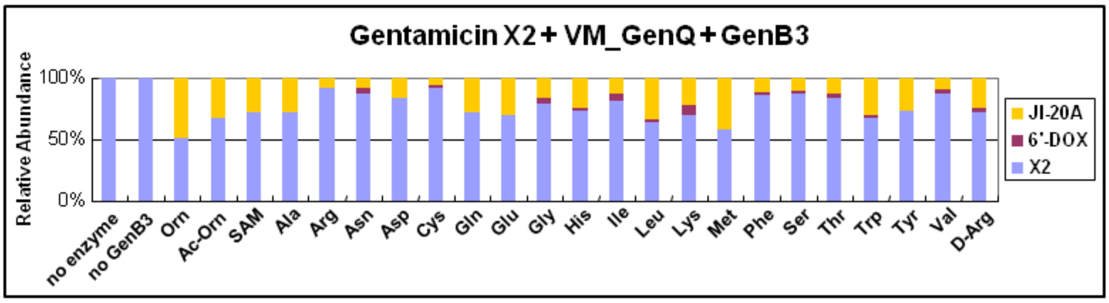

viii

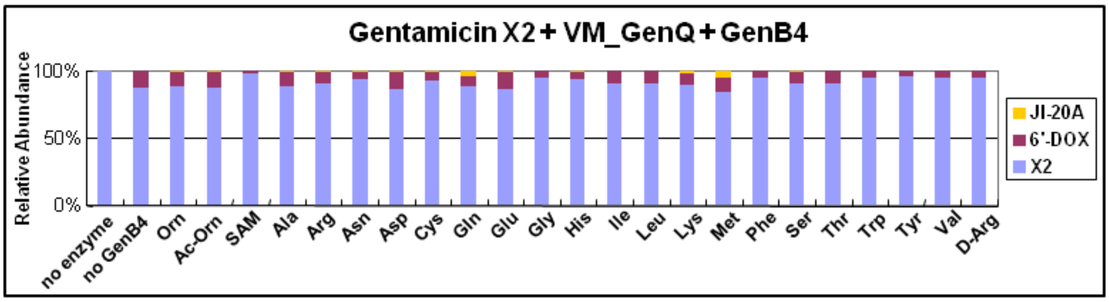

B

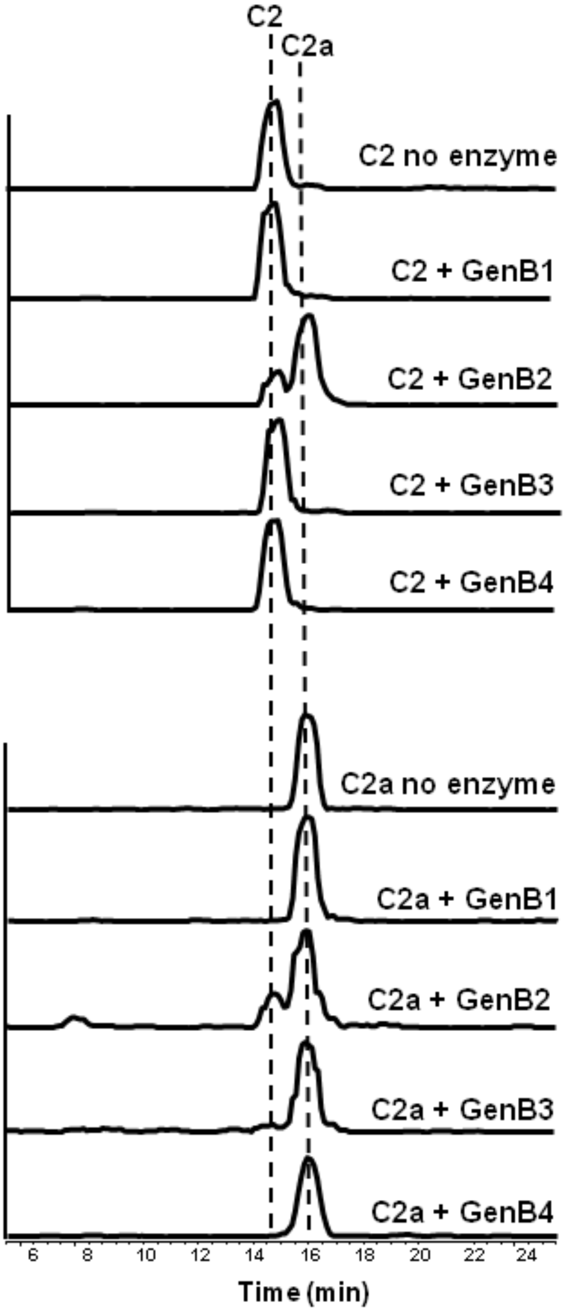
C2
C2a
C2 no enzyme
C2 + GenB1
C2 + GenB2
C2 + GenB3
C2 + GenB4
C2a no enzyme
C2a + GenB1
C2a + GenB2
C2a + GenB3
C2a + GenB4
6
8
10
12
14
16
18
20
22
24
Time (min)

List of oligonucleotide primers used in this study, related to Experimental Procedures

Primer	Oligonucleotide sequences (5' to 3')	Restriction site
genQ-L1	CTG**CATATG**CTCACCGCCCGGC	*Nde*I
genQ-L2	CCC**GAATTC**TCCCTGTGACCTGTCG	*Eco*RI
genQ-R1	ACC**GAATTC**GCCGCTACCGATCACAC	*Eco*RI
genQ-R2	GGC**AAGCTT**GATCGGAACCATCCGG	*Hin*dIII
genQ-CP1	CCTCCTCGTCACCGTGG	-
genQ-CP2	CAGGTGCTCAGCGTCCG	-
genQ-EP1	GTC**CATATG**ATCGGTAGCGGCGCC	*Nde*I
genQ-EP2	CGA**GAATTC**ACAGGGAGAAATCGG	*Eco*RI
neo11-EP1	CGC**CATATG**AAGCGCCTTCGAGGC	*Nde*I
neo11-EP2	CCG**GAATTC**TCAGACGTGCGCGGTGTG	*Eco*RI
genK-L1	GCG**CATATG**AGAACCGGCTTGATGC	*Nde*I
genK-L2	GCG**GAATTC**CACTGACCCTCGTCCC	*Eco*RI
genK-R1	GAG**GAATTC**CTTCACTTGATTACC	*Eco*RI
genK-R2	CAC**AAGCTT**CACCGGCGAGACG	*Hin*dIII
genK-CP1	CGGGCGAACCTTCGGGATA	-
genK-CP2	CCGTCAGCGTTGGCAATAA	-
genK-EP1	CGC**CATATG**AACGCGCTGGTGGCA	*Nde*I
genK-EP2	CCG**GAATTC**TCAGTGGGAAACCGCCTC	*Eco*RI
genB1-L1	GCA**CATATG**CTGGAGAATCCGTCTG	*Nde*I
genB1-L2	GTC**GAATTC**CTCTGGGAGGACTGAG	*EcoR*I
genB1-R1	ACC**GAATTC**GATGGTCATCGTGTCG	*EcoR*I
genB1-R2	GAA**AAGCTT**GACCGTCAACCTGGCG	*Hin*dIII
genB1-CK1	GAGTCGTCGTACTTCTGGATGGG	-
genB1-CK2	GCTGACCTGGGGTTGCTGC	-
genB2-L1	GCG**CATATG**TCACCGGCGAAGCGGC	*Nde*I
genB2-L2	CGT**GAATTC**GTCAGCGTTGGCAATA	*EcoR*I
genB2-R1	GTG**GAATTC**GTGACCGCTCAGCTCT	*EcoR*I
genB2-R2	GTC**AAGCTT**GTTGCAGGCGGTCCAG	*Hin*dIII
genB2-CK1	GCCGACTTCGACCTCTTCC	-
genB2-CK2	ATCCGCTGACCCCGTGCC	-

genB3-L1	CGT**CATATG**GCAACACCACGTCG	*Nde*I
genB3-L2	GAC**GAATTC**ATCGAGAAGGTGGTC	*EcoR*I
genB3-R1	CGA**GAATTC**GGTCCCGATGTCGTAG	*EcoR*I
genB3-R2	CTT**AAGCTT**TGGCGAGGGGCTCTGC	*Hin*dIII
genB3-CK1	CGCGTTACGGAAAGTAAAATCAC	-
genB3-CK2	CATCGAGGGCCACCACC	-
genB4-L1	CGC**CATATG**CAGCGGTTCAAGGGCG	*Nde*I
genB4-L2	GTC**GAATTC**CCGGCACAGAACTGAC	*EcoR*I
genB4-R1	GAT**GAATTC**ACGGTAGTTCATGTGC	*EcoR*I
genB4-R2	CGG**AAGCTT**TGTGTGAGCGGTACGG	*Hin*dIII
genB4-CK1	TGACTTCTGCCTCGACAACG	-
genB4-CK2	AAGCTCTACCTGGAGACCTTCC	-
genB4-genB3-L1	GGC**CATATG**CGGTGGCGACCC	*Nde*I
genB4-genB3-L2	CGC**GGTACC**AAGCCGGTTCTCA	*Kpn*I
genB4-genB3-R1	CAA**GGTACC**AGAATCCATGTCC	*Kpn*I
genB4-genB3-R2	AAT**AAGCTT**GGGAATTAGTCCAG	*Hin*dIII
genB4-genB3-CK1	CAGCGTTCCTCCGGTTTC	-
genB4-genB3-CK2	GGTAAGTGATCCGCAGTTGG	-
pGenQ-LM_up	ATCACGGCC**CATATG**CGGTACGGCCCGGAG	*Nde*I
pGenQ-VM_up	CCCAACCCGG**CATATG**CTCATCAGCGTTTC	*Nde*I
pGenQ-dn	TTGCCGGCACC**GAATTC**GATGGTCATCGTG	*Eco*RI
pGenB1-up	AATCGAGCGAC**CATATG**ACCATCGACATCG	*Nde*I
pGenB1-dn	AGGACCTGGTGG**GGATCC**ACAAGGAGTGAA	*Bam*HI
pGenB2-up	CGGAGGTTTCG**CATATG**ATTATTGCCAACG	*Nde*I
pGenB2-dn	CGGCCCCTGCC**GGATCC**GTCAGAGCTGAGC	*Bam*HI
pGenB3-up	TTG GGGATAG**CATATG**GCAGTCGCCGACCA	*Nde*I
pGenB3-dn	ATCTTCACCGGCCCTG**GAATTC**CTACGCC A	*Eco*RI
pGenB4-up	CGTGGTGGG**CATATG**AACTACCGTGAGTTG	*Nde*I
pGenB4-dn	CGGCCCTCGAACCG**GAATTC**CTCAAGGTCA	*Eco*RI

Primer pairs for amplification of left- or right-flanking fragments of a target gene, for PCR/sequencing confirmation, and for cloning target genes for over-expression are marked with suffixes –L1/–L2, -R1/-R2, CK1/CK2, or –up/-dn, respectively.

Table S1, related to FIgure 3. LC-HRMS analysis of *genB* knock-out mutants

Strain	Gentamicin C complex production (×10^5)					Intermediates production (×10^5)			
	C1a	C2b	C2	C2a	C1	X2	JI-20A	G418	JI-20B
wild type	8.8	2.8	11.9	1.1	44.6	8.5	1.5	8.2	4.3
ΔgenB1	0.4	2.9	1.2	1.7	14.6	6.8	0.5	15.9	2.5
ΔgenB2	0.8	0.1	ND	7.6	ND	7.1	0.7	0.7	1.0
ΔgenB3	ND	ND	ND	ND	ND	7.2	19.1	10.8	75.4
ΔgenB4	ND	ND	ND	ND	ND	11.0	2.7	14.1	8.7
ΔgenB2ΔgenB3	ND	ND	ND	ND	ND	8.6	2.7	8.8	20.5
ΔgenB2ΔgenB4	ND	ND	ND	ND	ND	7.6	2.0	1.2	3.7
ΔgenB4ΔgenB1	ND	ND	ND	ND	ND	5.2	1.4	18.6	6.7
ΔgenB2ΔgenB1	0.3	0.1	ND	15.3	ND	4.0	0.5	10.7	2.9
ΔgenB1ΔgenB3	ND	ND	ND	ND	ND	1.5	0.1	43.0	1.4
ΔgenB4ΔgenB3	ND	ND	ND	ND	ND	9.7	1.1	17.0	6.3
ΔgenB2ΔgenB3ΔgenB1	ND	ND	ND	ND	ND	3.4	0.1	12.8	0.1
ΔgenB4ΔgenB1ΔgenB2	ND	ND	ND	ND	ND	1.9	0.1	40.3	3.4
ΔgenB4ΔgenB1ΔgenB3	ND	ND	ND	ND	ND	3.3	0.1	20.8	0.8
ΔgenB2ΔgenB4ΔgenB3	ND	ND	ND	ND	ND	7.4	2.5	20.8	35.2
ΔgenB4ΔgenB1ΔgenB3ΔgenB2	ND	ND	ND	ND	ND	2.3	ND	142.0	ND

Table S2, related to Experimental Procedures. Plasmids used in this study

Plasmid	Description	Reference
pUC18	Sub-cloning vector	Sambrook and Maniatis
pET28a(+)	Vector for protein expression	Invitrogen
pIB139	Site-specific integrative vector with P*ermE** promoter	Wilkinson et al., 2002; Del Vecchio et al., 2003
pYH7	*E. coil* - *Streptomyces* shuttle vector	Sun et al., 2008
pWHU77	pIB139 derivative with Tsr^R	This study
pYH286	*genQ* in-frame deletion construct	This study
pWHU1	*genK* in-frame deletion construct	This study
pWHU67	ΔgenK complementation construct containing *genK* under control of P*ermE** promoter	This study
pWHU163	ΔgenQ complementation construct containing *genQ* under control of P*ermE** promoter	This study
pWHU165	ΔgenQ complementation construct containing *neo11* under the control of the P*ermE** promoter	This study
pWHU4	*genB1* in-frame deletion construct	This study
pWHU2	*genB2* in-frame deletion construct	This study
pWHU5	*genB3* in-frame deletion construct	This study
pWHU3	*genB4* in-frame deletion construct	This study
pWHU43	*genB3* and *genB4* double in-frame deletion construct	This study
pET28/LM_genQ	pET28a(+) vector with VM_*genQ* gene insert	This study
pET28/VM_genQ	pET28a(+) vector with LM_*genQ* gene insert	This study
pET28/genB1	pET28a(+) vector with *genB1* gene insert	This study
pET28/genB2	pET28a(+) vector with *genB2* gene insert	This study
pET28/genB3	pET28a(+) vector with *genB3* gene insert	This study
pET28/genB4	pET28a(+) vector with *genB4* gene insert	This study

Table S3, related to Experimental Procedures. In-frame deletion mutants and complemented strains used in this study

**Gene knockout mutant**	**Parent strain**	**Plasmid used to achieve deletion**
ΔgenK	wild type	pWHU1
ΔgenQ	wild type	pYH286
ΔgenQΔgenK	ΔgenQ	pWHU1
ΔgenB1	wild type	pWHU4
ΔgenB2	wild type	pWHU2
ΔgenB3	wild type	pWHU5
ΔgenB4	wild type	pWHU3
ΔgenB2ΔgenB1	ΔgenB2	pWHU4
ΔgenB1ΔgenB3	ΔgenB1	pWHU5
ΔgenB4ΔgenB3	ΔgenB4	pWHU43
ΔgenB2ΔgenB3	ΔgenB2	pWHU5
ΔgenB2ΔgenB4	ΔgenB2	pWHU3
ΔgenB4ΔgenB1	ΔgenB4	pWHU4
ΔgenB4ΔgenB1ΔgenB2	ΔgenB4ΔgenB1	pWHU2
ΔgenB4ΔgenB1ΔgenB3	ΔgenB4ΔgenB1	pWHU43
ΔgenB2ΔgenB3ΔgenB1	ΔgenB2ΔgenB3	pWHU4
ΔgenB2ΔgenB4ΔgenB3	ΔgenB2ΔgenB4	pWHU43
ΔgenB4ΔgenB1ΔgenB3ΔgenB2	ΔgenB4ΔgenB1ΔgenB3	pWHU2
**Complemented mutant**	**Parent strain**	**Plasmid for complementation**
ΔgenK::*genK*	ΔgenK	*genK* gene in pWHU77
ΔgenQ::*genQ*	ΔgenQ	*genQ* gene in pWHU77
ΔgenQ::*neo11*	ΔgenQ	*neo11* gene in pWHU77
