# Supplementary material for: Specificity and Promiscuity at the Branch Point in Gentamicin Biosynthesis
Source: Chem Biol. 2014 May 22;21(5):608–18. doi: 10.1016/j.chembiol.2014.03.005 (PMC4039129; doi:10.1016/j.chembiol.2014.03.005)
Supplement: Document S2. Article plus Supplemental Information [file mmc2.pdf]

Specificity and Promiscuity at the Branch Point in Gentamicin Biosynthesis

Junhong Guo,[1,4] Fanglu Huang,[2,4] Chuan Huang,[1] Xiaobo Duan,[1] Xinyun Jian,[1] Finian Leeper,[3] Zixin Deng,[1] Peter F. Leadlay,[2,*] and Yuhui Sun[1,*]
[1]Key Laboratory of Combinatorial Biosynthesis and Drug Discovery (Ministry of Education), and School of Pharmaceutical Sciences, Wuhan University, Wuhan, Wuchang 430071, People's Republic of China
[2]Department of Biochemistry, University of Cambridge, Cambridge CB2 1GA, UK
[3]Department of Chemistry, University of Cambridge, Cambridge CB2 1EW, UK
[4]Co-first authors
*Correspondence: pfl10@cam.ac.uk (P.F.L.), yhsun@whu.edu.cn (Y.S.)
http://dx.doi.org/10.1016/j.chembiol.2014.03.005
This is an open access article under the CC BY license (http://creativecommons.org/licenses/by/3.0/).

SUMMARY

Gentamicin C complex is a mixture of aminoglycoside antibiotics used to treat severe Gram-negative bacterial infections. We report here key features of the late-stage biosynthesis of gentamicins. We show that the intermediate gentamicin X2, a known substrate for C-methylation at C-6′ to form G418 catalyzed by the radical SAM-dependent enzyme GenK, may instead undergo oxidation at C-6′ to form an aldehyde, catalyzed by the flavin-linked dehydrogenase GenQ. Surprisingly, GenQ acts in both branches of the pathway, likewise oxidizing G418 to an analogous ketone. Amination of these intermediates, catalyzed mainly by aminotransferase GenB1, produces the known intermediates JI-20A and JI-20B, respectively. Other pyridoxal phosphate-dependent enzymes (GenB3 and GenB4) act in enigmatic dehydroxylation steps that convert JI-20A and JI-20B into the gentamicin C complex or (GenB2) catalyze the epimerization of gentamicin C2a into gentamicin C2.

INTRODUCTION

Gentamicins are clinically valuable aminoglycoside antibiotics containing modified sugar units, including an unusual aminocyclitol ring (2-deoxystreptamine [2-DOS]; Houghton et al., 2010; Park et al., 2013). Gentamicins inhibit protein synthesis by interfering with initiation, codon fidelity, and translocation. They are isolated from the filamentous bacterium *Micromonospora echinospora* as gentamicin C complex, a mixture of five components (Figure 1). Gentamicin C complex is a mainstay of the treatment of life-threatening sepsis caused by Gram-negative bacterial infections. It also shows promise as a lead for treatment of inherited diseases associated with premature stop codons (Hainrichson et al., 2008; Linde and Kerem, 2008) and acts as a sensitizing agent for NCI-H460 lung cancer cells, increasing the efficacy of various anticancer agents (Cuccarese et al., 2013). Unfortunately, these vital drugs entail a serious risk of kidney damage and hearing loss (Bockenhauer et al., 2009). Most studies on the mechanisms of gentamicin toxicity have used the commercial mixture, despite evidence that an individual component of the gentamicin mixture may have lower toxicity (Sandoval et al., 2006; Kobayashi et al., 2008). As a prerequisite for engineering to favor the production of a particular gentamicin, we have undertaken a detailed investigation of the late steps in the pathway.

The outlines of the gentamicin pathway were established by isolating blocked mutants after random mutagenesis and monitoring the fate of compounds fed to such blocked strains (Testa and Tilley, 1975, 1976; Kase et al., 1982a, 1982b). It was shown that the pathway branches at gentamicin X2 (Figure 1) and the final steps involve a series of oxidations, transaminations, and C- and N-methylations, as well as the intriguing loss of two hydroxyl groups from one of the sugar rings. Many biosynthetic gene clusters that govern aminoglycoside biosynthesis have been sequenced (Ota et al., 2000; Unwin et al., 2004; Kharel et al., 2004a, 2004b, 2004c; Huang et al., 2005; Subba et al., 2005; Aboshanab, 2005; Hong et al., 2009). As a result, rapid progress has been made in identifying the common enzymatic steps that lead to the 2-DOS scaffold, and thence to the pseudodisaccharide paromamine (Figure 1; Llewellyn and Spencer, 2006; Thibodeaux et al., 2008; Kudo and Eguchi, 2009; Kudo et al., 2009).

The intermediate pseudotrisaccharide gentamicin A2 (Figure 1) has been produced by heterologous expression of a subset of the gentamicin biosynthetic genes in the host strain *Streptomyces venezuelae* (Park et al., 2008). This has enabled a sharper focus on those genes likely to govern the later steps of gentamicin biosynthesis. Specific late gene knockouts have been made in *genD1* and *genK*, both of which are predicted based on bioinformatic analysis (Figure S1A available online) to encode class B radical S-adenosylmethionine-dependent C-methyltransferases (Zhang et al., 2012). The replacement of *genD1*, also known as *gntE* (Unwin et al., 2004) or *gtmI* (Kharel et al., 2004c), by a thiostrepton resistance gene (*tsr*) led to accumulation of gentamicin A2 (Figure 1; Kim et al., 2008). These authors interpreted their result as meaning that GenD1 catalyzes N-methylation at the 3′′-NH_2 rather than C-methylation at C-4′′ of the xylose ring of gentamicin A2; but this, and the exact order of events, remains to be established. Disruption of *genK*, also known as *gntK* (Unwin et al., 2004) or *gacD* (Kharel et al., 2004c), has been shown to result in preferential accumulation of gentamicin C1a (Karki

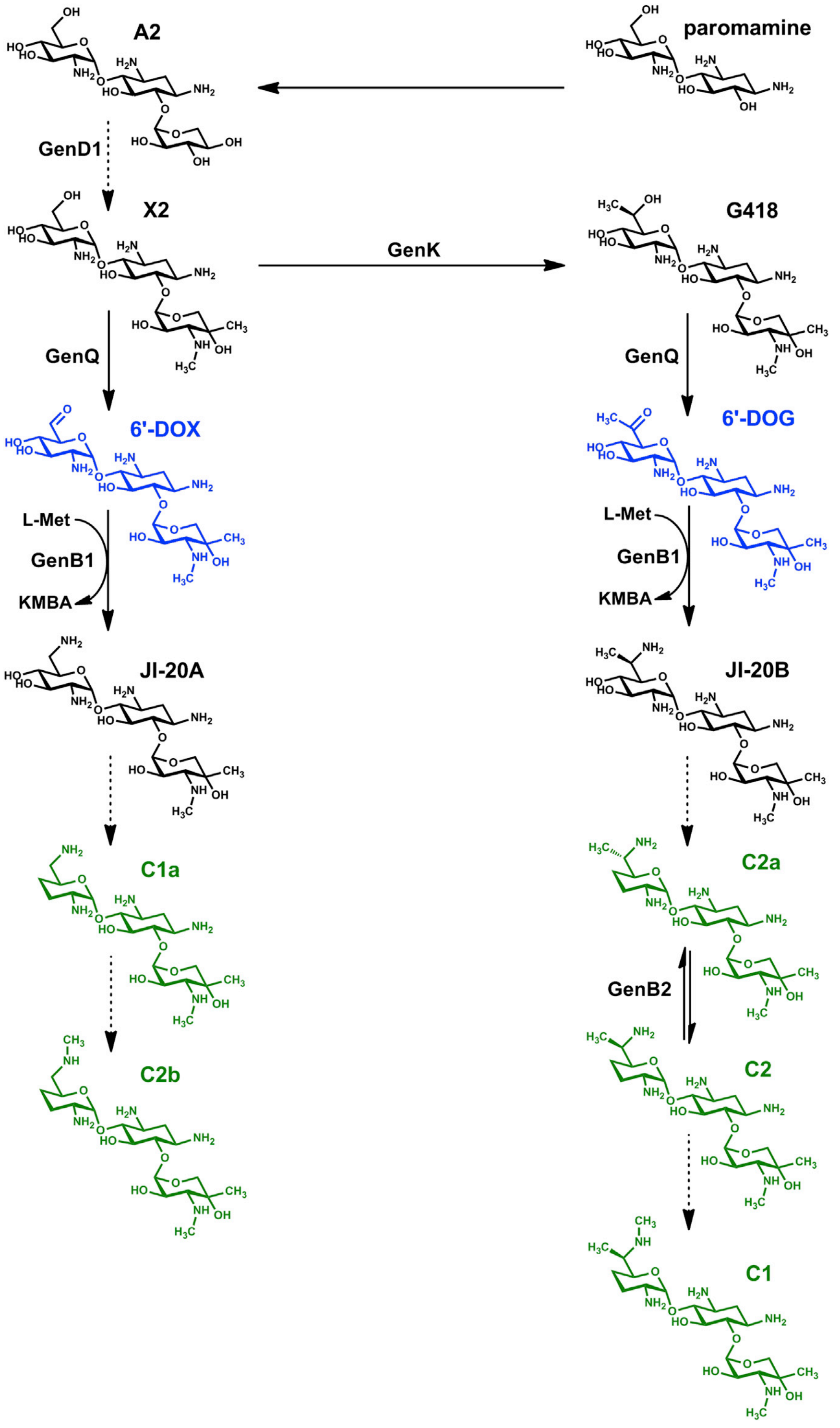

Figure 1. Proposed Biosynthetic Pathway from Paromamine to the Gentamicin C Complex

The early steps in the pathway lead from the pseudodisaccharide paromamine to the branch point at gentamicin X2. The five main components of the clinically-used gentamicin C complex are shown in green. The proposed roles of the radical S-adenosylmethionine-dependent methyltransferases GenD1 and GenK are indicated. The role of GenK has been established by previous in vitro work. Intermediates 6′-DOX and 6′-DOG, newly-detected in this study, are shown in blue. The roles of dehydrogenase GenQ and aminotransferase GenB1 are revealed in this study, as well as the involvement of GenB3 and GenB4 in the steps from intermediates JI-20A and JI-20B to the gentamicin C complex, and of GenB2 in epimerization of gentamicin C2a to gentamicin C2. For further details see the text. KMBA, 2-keto-3-methylthiobutyric acid; 6′-DOG, 6′-dehydro-6′-oxo-G418; 6′-DOX, 6′-dehydro-6′-oxo-gentamicin X2.

et al., 2012; Hong and Yan, 2012; Li et al., 2013). GenK has also been demonstrated to catalyze C-methylation in vitro of C-6′ in gentamicin X2 to form G418 (Kim et al., 2013).

By analysis of the gentamicin-related metabolites accumulated in strains bearing single and multiple specific gene deletions, by following the bioconversion of specific intermediates fed to such mutants, and by assaying individual steps using purified recombinant enzymes, we demonstrate that control at the branch point is shared between GenK and the flavin-linked dehydrogenase GenQ. Importantly, GenQ operates in both branches of the pathway, and is essential for the oxidation of both X2 and G418 (Figure 1). We have also obtained fresh insights into the respective catalytic contributions of four pyridoxal phosphate-dependent enzymes GenB1, GenB2, GenB3, and GenB4.

RESULTS

GenK Acts at the X2 Branch Point and Is Essential for the Production of G418 and Gentamicins C2a, C2, and C1

To confirm the previously established role of *genK* at the gentamicin X2 branch point, this gene was knocked out by targeted in-frame deletion (Figure S2(1)A). The mutant was confirmed by PCR and Southern hybridization (Figures S2(1)B and S2C). Liquid chromatography-electrospray ionization high-resolution mass spectrometry (LC-ESI-HRMS) analysis confirmed that in this mutant, production of G418, JI-20B, and gentamicins C1, C2, and C2a was abolished (Figure 2C and Figure S3B). In contrast, levels of gentamicin X2, JI-20A, and gentamicins C1a and C2b were all substantially increased compared to wild-type (Figure 2C and Figure S3B). Complementation of the ΔgenK mutant, carried out by using plasmid pWHU67 containing *genK* under the control

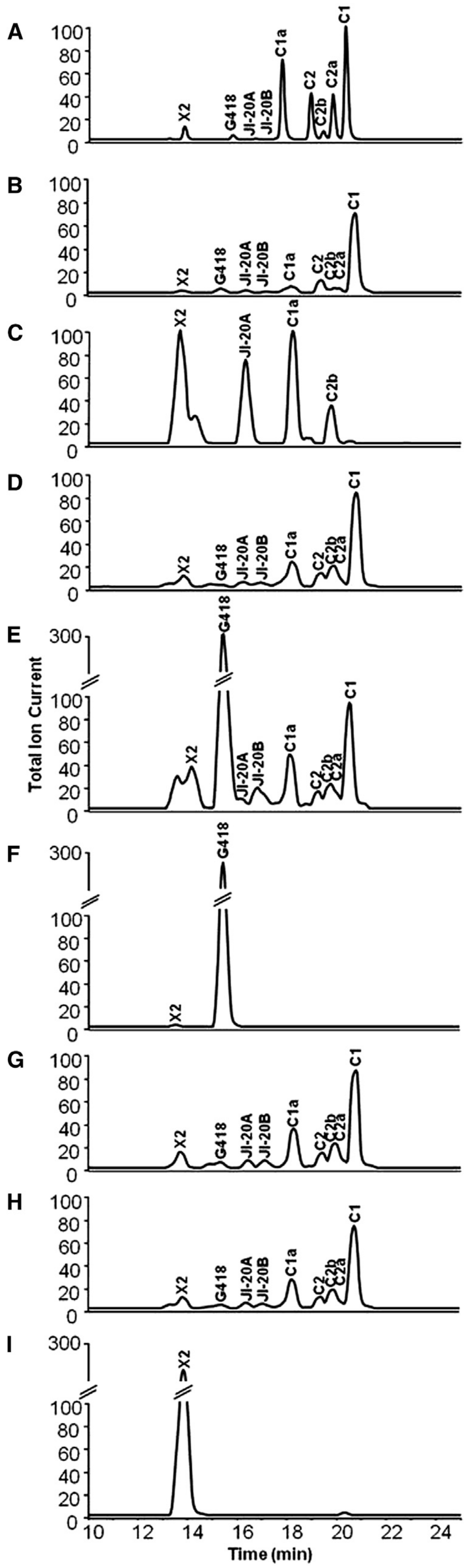

Figure 2. LC-ESI_HRMS Analysis of the Production of Gentamicin C Complex and Gentamicin-Related Intermediates by *Micromonospora echinospora*

(A) gentamicin C standard mixture.
(B) Wild-type.
(C) ΔgenK mutant.
(D) ΔgenK::*genK* mutant.
(E) ΔgenK mutant fed with G418.
(F) ΔgenQ mutant.
(G) ΔgenQ::*genQ* mutant.
(H) ΔgenQ::*neo11* mutant.
(I) ΔgenQΔgenK mutant.
See also Figures S1–S3. For details of the LC-ESI-HRMS analysis, see the Supplemental Information.

of the P*ermE** promoter (Figure S2(4)A), restored the production of gentamicin C complex and of various intermediates to wild-type levels (Figure 2D). Chemical complementation of the ΔgenK mutant by feeding G418 also restored production of JI-20B, gentamicin C2, C2a (the C-6′ epimer of C2), and C1 (Figure 2E).

Dehydrogenase GenQ Is Essential in Both Branches of the Gentamicin Pathway that Diverges from X2

The gene *genQ* (also known as *gntX* (Unwin et al., 2004) or *gacJ* (Kharel et al., 2004c)) is predicted to encode a flavin-linked dehydrogenase with significant sequence identity to enzymes catalyzing similar steps in other aminoglycoside pathways (Figure S1B). GenQ was therefore an attractive candidate to act at the X2 branch point and initiate the biosynthesis of C1a and the minor component C2b (also known as sagamicin; Figure 1). To investigate the role of *genQ*, a 1494 bp internal DNA fragment was deleted in-frame (Figure S2(2)A). The mutant was confirmed by PCR and Southern hybridization (Figures S2(2)B and S2C). LC-ESI-HRMS analysis revealed that production was abolished in the ΔgenQ mutant not only of JI-20A and the downstream gentamicins C1a and C2b, but unexpectedly also of JI-20B and gentamicins C2, C2a, and C1 (Figure 2F and Figure S3B). Meanwhile, G418 was accumulated to levels 100-fold higher than wild-type (Figure S3B). These data strongly suggest that *genQ* is not only involved at the X2 branch point in the first step toward C1a and C2b, but is also essential in the other branch of the pathway for the oxidation of G418 that leads on to JI-20B and finally to C2, C2a, and C1. The LC-ESI-HRMS analysis did not detect 6′-dehydro-6′-oxo-G418 (6′-DOG) or 6′-dehydro-6′-oxo-gentamicin X2 (6′-DOX; Figure 1), the presumed products of GenQ-catalyzed oxidation.

The dual function of *genQ* was further supported by showing that the ΔgenQ mutant could be homologously complemented by introduction of pWHU163, in which *genQ* is under the control of the constitutive promoter P*ermE** (Figure 2G and Figure S2(4)B). This mutant was also heterologously complemented by introduction of pWHU165 (Figure 2H and Figure S2(4)C) containing *neo11*, a gene from the neomycin biosynthetic pathway that is homologous to *genQ* (Huang et al., 2007). LC-ESI-HRMS analysis of fermentation extracts from the mutants ΔgenQ::*genQ* and ΔgenQ::*neo11* demonstrated that all components of the gentamicin C complex were restored to wild-type levels (Figures 2G and 2H). The ΔgenQΔgenK mutant was also generated by targeted in-frame deletions (Figure S2(3)), and LC-ESI-HRMS analysis showed that, as expected, only

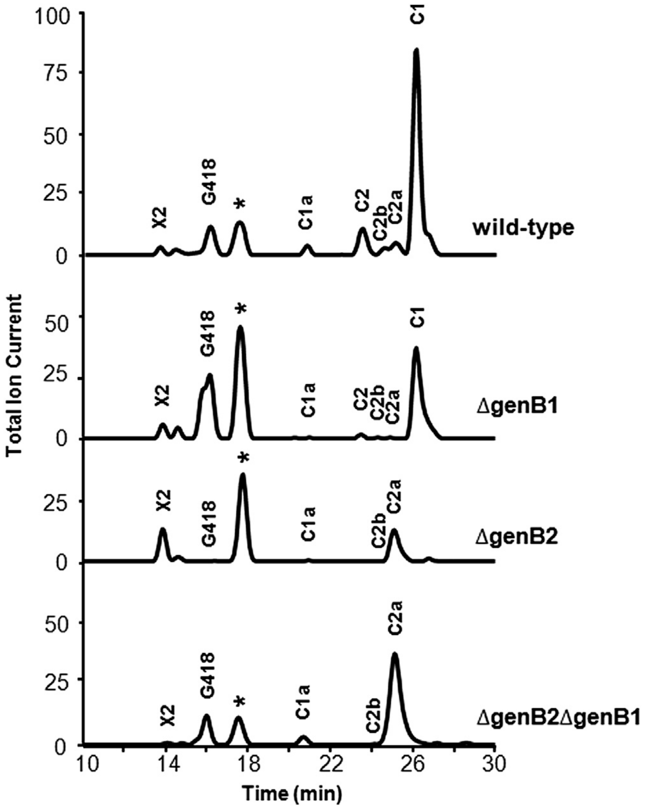

Figure 3. Production of Gentamicin C Components and Related Intermediates by *M. echinospora genB* Mutants

Data are shown for three *genB* mutants that continue to produce gentamicin C components. Data for a further 12 *genB* mutants (Figure S4B) that were found to produce no gentamicin C complex are shown in Table S1. The starred peak is from an unknown metabolite with the same mass as gentamicin X2.

gentamicin X2 was accumulated, at levels over 100-fold higher than in the wild-type (Figure 2I and Figure S3B).

GenB1 Is the Chief Aminotransferase Partner for GenQ while GenB3 and GenB4 Participate in Dehydroxylation

The gentamicin biosynthetic gene cluster in *M. echinospora* contains four genes that appear to encode class III pyridoxal phosphate-dependent transaminases (Figure S4A), any of which might catalyze the transamination steps that lead in parallel to JI-20A and JI-20B, respectively. The *genB1* gene was deleted in-frame, and the identity of the resulting mutant was confirmed by PCR and Southern blot analysis (Figure S4B(1)). Similarly, *genB2*, *genB3*, and *genB4* were each individually disrupted by in-frame deletion (Figures S4B(2) to ~S4B(4)). Starting from these mutants, similar methods were used to generate all six double mutants (Figure S4B(5) to ~S4B(10)), all four triple mutants (Figures S4B(11) to ~S4B(14)), and the quadruple mutant ΔgenB4ΔgenB1ΔgenB3ΔgenB2 (Figure S4B(15)). Each of these mutant strains was fermented in liquid medium, as detailed in the Experimental Procedures section, and gentamicin C complex and related metabolites were extracted, purified by ion exchange and submitted to LC-ESI-HRMS analysis (Figure 3 and Table S1) for comparison with wild-type.

This analysis revealed that in the quadruple mutant only X2 and G418 were produced (the latter at levels about 18-fold higher than in wild-type), a result that argues against any contribution to transamination activity from enzymes other than these four. In fact of the 15 mutants examined (Table S1), only ΔgenB1, ΔgenB2, and the double mutant ΔgenB2ΔgenB1 produced any gentamicin C complex components (Figure 3). Deletion of *genB1* substantially lowered, but did not abolish, the levels of individual components of the gentamicin C complex. This argues strongly that at least one other aminotransferase can partially fulfill the role of GenB1. Nevertheless, these data are consistent with GenB1 making the chief contribution to amination of both 6′-DOX and 6′-DOG. In the ΔgenB2 mutant and in the ΔgenB2ΔgenB1 double mutant, levels of gentamicins C1a and C2b in the "left-hand" branch (Figure 1) were significantly reduced. In the "right-hand" branch, production of C2a was increased more than 7-fold, while C2 and C1 production was completely blocked. Initially, we identified the C2a peak (Figure 3) as C2b from the "left-hand" branch (these metabolites have similar retention times, the same mass and the same MS-MS fragmentation pattern). However, further analysis and spiking with authentic C2a, C2, and C1 confirmed that C2a is the product (Figure S5). This finding leads directly to a proposal for the role of GenB2 in the pathway (see the Discussion section). All *genB* mutants continued to produce the known late-stage biosynthetic intermediates: gentamicin X2 at the branch point, G418 and JI-20B on the "right-hand" branch, and JI-20A on the "left-hand" branch of the pathway (Figure 1) at approximately wild-type levels (Figure 3 and Table S1). Again, these results show that other aminotransferases can substitute for GenB1.

Deletion of either *genB3* or *genB4*, alone or in combination with other *genB* genes, abolished the production of all five gentamicin C compounds (Table S1). For the ΔgenB3 mutant, both JI-20A and JI-20B were present at higher (more than 10-fold) levels than in wild-type, consistent with the likely involvement of GenB3 in the enigmatic dehydroxylation steps of gentamicin biosynthesis, rather than in amination of X2 and G418. In the ΔgenB4 mutant, too, and also in the double mutant ΔgenB4ΔgenB3, the C complex was completely lacking whereas intermediates X2, G418, JI-20A, and JI-20B were still present at either wild-type or slightly elevated levels. This demonstrated that GenB4 also has an essential role in dehydroxylation, and that this role is distinct from that of GenB3. Other double mutants showed different levels of intermediates X2, G418, JI-20A, and JI-20B that reflect the differing contribution of each GenB enzyme to transamination. When the triple mutants were examined, in which only one of the four *genB* genes remains functional, ΔgenB2ΔgenB4ΔgenB3 was found to accumulate wild-type levels of JI-20A and high levels of G418 and JI-20B, confirming that the presence of GenB1 alone suffices for the reaction of both X2 and G418. The other triple mutants all showed very low levels of both JI-20A and JI-20B compared to wild-type, again indicating a minor role for GenB2, GenB3, or GenB4 in catalyzing either transamination.

Recombinant GenQ Catalyzes 6′-Oxidation of G418 and Gentamicin X2

Recombinant GenQ was expressed in *Escherichia coli* to assay its ability to catalyze the 6′-dehydrogenation of both gentamicin X2 and G418. GenQ as published is shorter (~40 amino acid

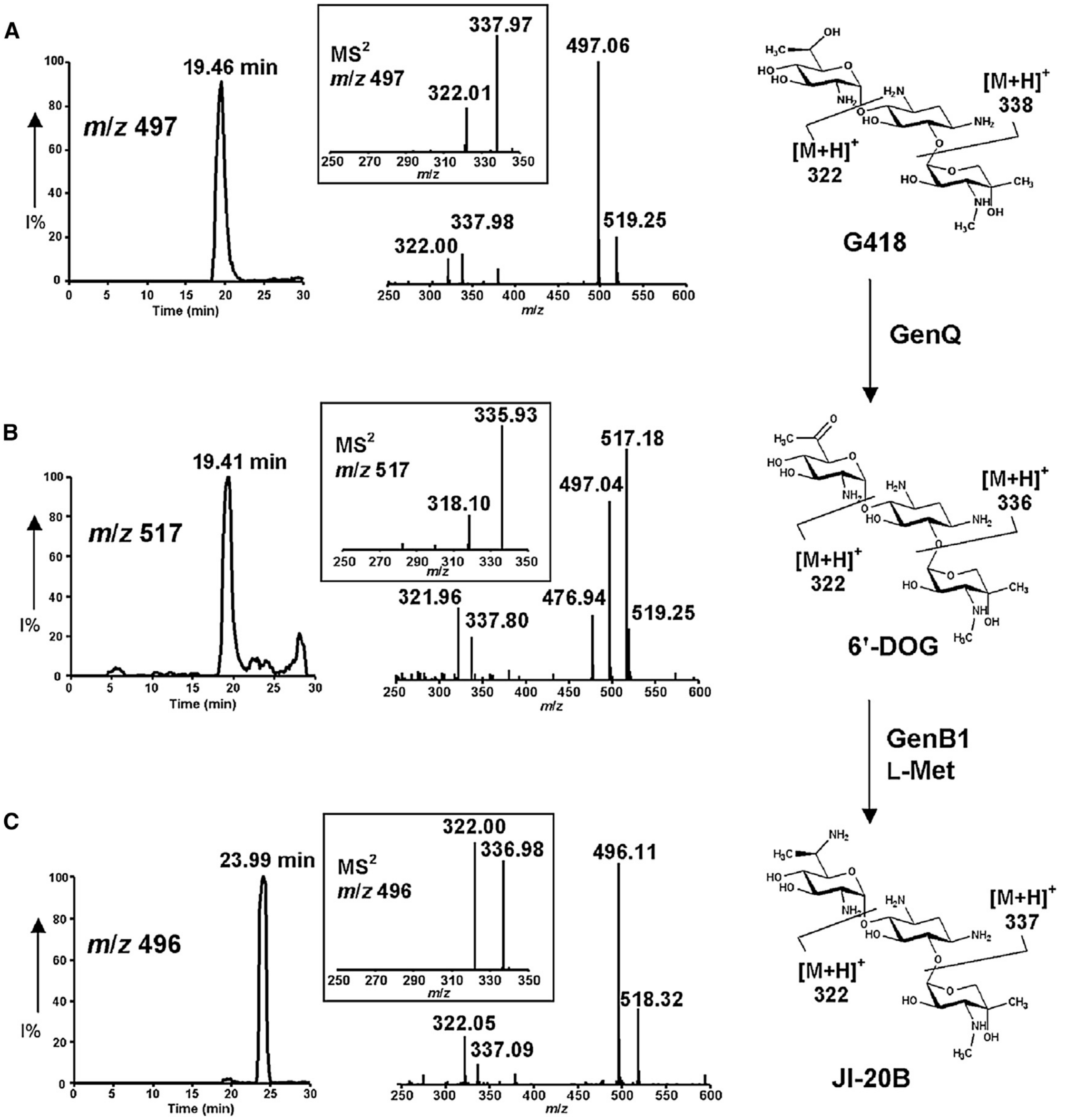

Figure 4. LC-MS Analysis of GenQ/GenB1-Catalyzed Dehydrogenation and Transamination of G418

Selective ion monitoring was carried out on (A) $[M+H]^+$ (*m/z* 497) and $[M+Na]^+$ (*m/z* 519) ions of G418; (B) the $[M+Na]^+$ (*m/z* 517) ion of 6′-DOG, the product of GenQ-catalyzed 6′-dehydrogenation of G418; (C) $[M+H]^+$ (*m/z* 496) and $[M+Na]^+$ (*m/z* 518) ions of JI-20B, the product of GenB1 catalyzed 6′-amination of 6′-DOG using L-methionine as an amino donor. MS/MS analysis of $[M+H]^+$ (*m/z* 497), $[M+Na]^+$ (*m/z* 517), and $[M+H]^+$ (*m/z* 496) ions is also shown. The peak at *m/z* 318.1 in Figure 4B arises by further loss of water from fragment *m/z* 335.93. See also Figures S6 and S7.

residues) at the N terminus than close homologs in other aminoglycoside biosynthetic pathways (Figure S1B). We therefore amplified two longer versions of *genQ*: One, designated LM_*genQ*, starts from the rare start codon CTG (Missiakas et al., 1993) and provides an extra 19 N-terminal residues, whereas the second version, VM_*genQ*, provides an extra 38 N-terminal residues absent in the original GenQ protein (Figure S6A). N-His_6-tagged VM_GenQ was expressed as a soluble protein in *E. coli* (Figure S6B). Purified VM_GenQ had a yellow color and major absorption peaks at 370 nm and 445 nm (Figure S6C), indicating the presence of a flavin prosthetic group. A solution of VM_GenQ was deproteinized and flavin adenine dinucleotide (FAD) (*m/z* 786 $[M+H]^+$ and *m/z* 808 $[M+Na]^+$) was detected in the supernatant with LC-ESI-MS analysis. LM_GenQ did not contain bound flavin, so was not studied further.

VM_GenQ (hereafter referred to as GenQ) was tested for dehydrogenase activity using G418 and gentamicin X2 as substrates, as described in the Experimental Procedures. LC-ESI-MS analysis of the reaction mixture containing GenQ and G418 revealed a new peak eluting at a slightly earlier retention time, with *m/z* = 495 $[M+H]^+$ and *m/z* = 517 $[M+Na]^+$; Figure 4B), two mass units less than G418 (Figure 4A), as expected for the conversion of G418 into 6′-DOG. GenQ also showed activity with X2 as the substrate (Figure 5A), producing a compound with the

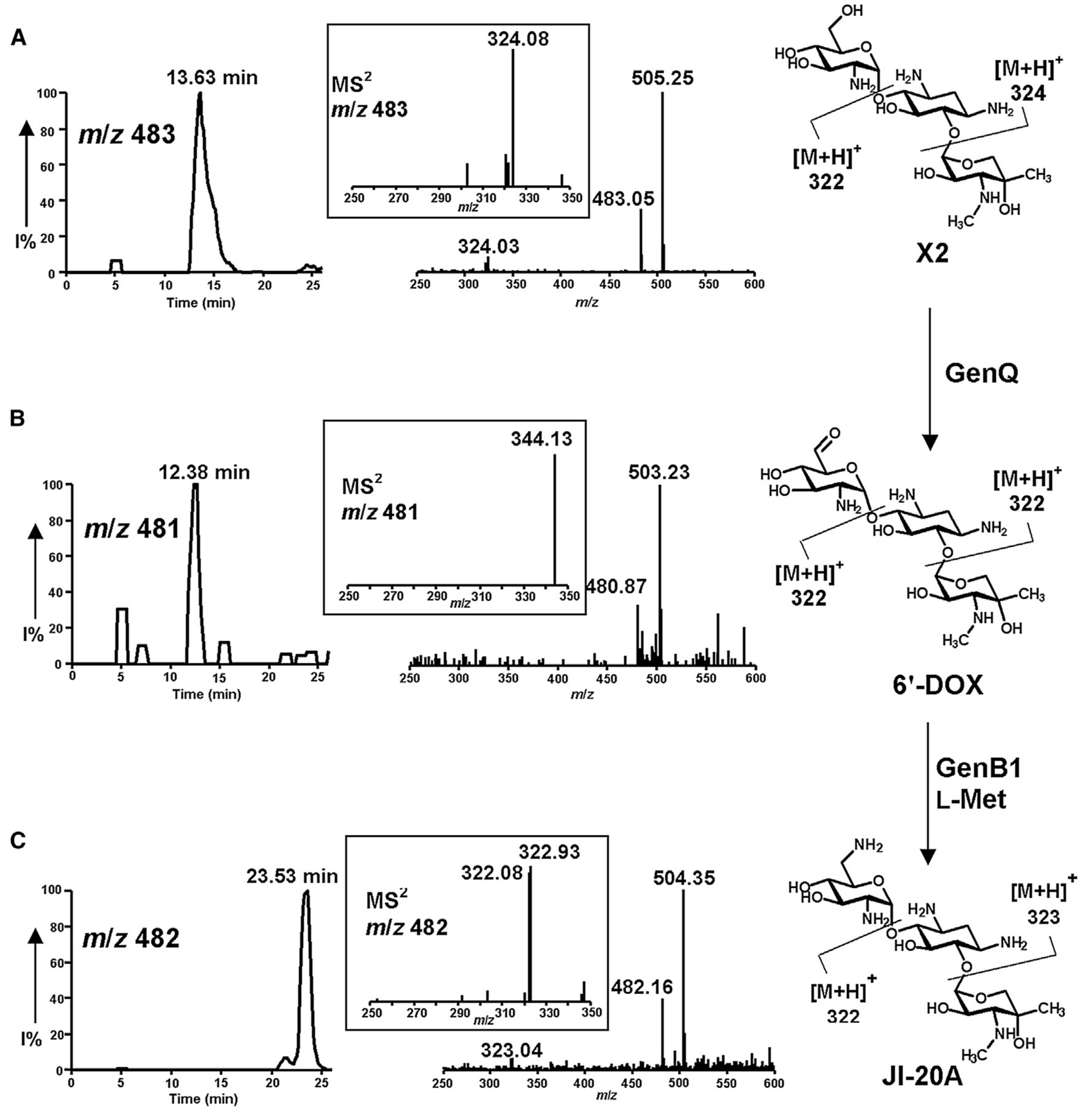

Figure 5. LC-MS Analysis of GenQ/GenB1-Catalyzed Dehydrogenation and Transamination of Gentamicin X2

Selective ion monitoring was carried out on (A) $[M+H]^+$ (*m/z* 483) and $[M+Na]^+$ (*m/z* 505) ions of gentamicin X2; (B) $[M+H]^+$ (*m/z* 481) and $[M+Na]^+$ (*m/z* 503) ions of 6′-DOX, the product of GenQ-catalyzed 6′-dehydrogenation of gentamicin X2; (C) $[M+H]^+$ (*m/z* 482) and $[M+Na]^+$ (*m/z* 504) ions of JI-20A, the product of GenB1-catalyzed 6′-amination of 6′-DOX using L-methionine as an amino donor. MS/MS analysis of $[M+H]^+$ (*m/z* 483), $[M+H]^+$ (*m/z* 481) and $[M+H]^+$ (*m/z* 482) ions are also shown. See also Figures S6 and S7.

mass expected of 6′-DOX (*m/z* = 481 $[M+H]^+$, *m/z* = 503 $[M+Na]^+$; Figure 5B). Tandem mass spectrometry (MS-MS) analysis of the species with *m/z* 517 and *m/z* 481 gave fragmentation patterns fully consistent with dehydrogenation having occurred in the side chain of the 6′-methyl-glucosamine ring of G418 and in the glucosamine ring of X2, respectively.

Multiple Aminotransferases Are Capable of C-6′-Transamination of Gentamicin Biosynthetic Intermediates In Vitro

The gene products of *genB1*, *genB2*, *genB3*, and *genB4* were all considered plausible candidate enzymes to partner GenQ in 6′-dehydrogenation-transamination, based on their significant sequence similarity to Neo18, a previously-characterized glutamate: 6′-dehydroparomamine aminotransferase (Huang et al., 2007; Figure S4A). Recombinant GenB1, GenB2, GenB3, and GenB4 were expressed in *E. coli* (Figure S6B). All four purified proteins were yellow and had UV-visible absorption spectra typical of aminotransferases containing bound pyridoxal-5′-phosphate cofactor (Figure S6D). GenB1, GenB2, GenB3, and GenB4 were each tested for their ability to catalyze the transamination of the aldehyde 6′-DOX to form JI-20A; and to catalyze transamination of the ketone 6′-DOG to form JI-20B. In these coupled assays, carried out as detailed in Experimental Procedures, L-ornithine, *N*-acetyl-L-ornithine, D-arginine, S-adenosyl-L-methionine, and all proteinogenic amino acids except L-proline

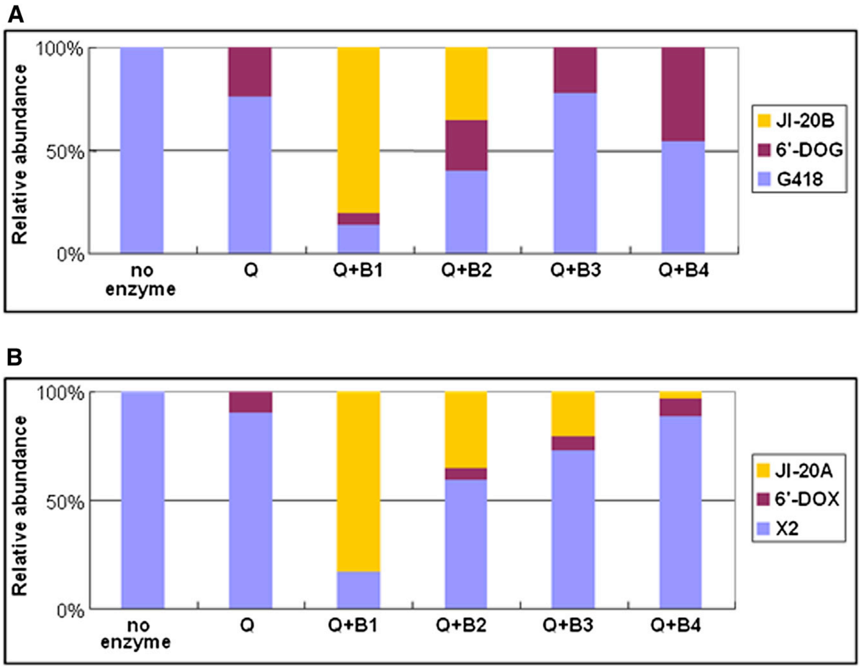

Figure 6. Coupled In Vitro Analysis of Aminotransferase Activities of Individual GenB Enzymes

(A) Amination of 6′-DOG generated in situ from G418 by the reaction of GenQ.

(B) Amination of 6′-DOX generated in situ from gentamicin X2 by the reaction of GenQ; Reaction mixtures were analyzed after incubation for 16 hr. The amino donor was L-methionine.

See also Figures S6 and S7.

were tested as potential amino donors. The reaction mixtures were analyzed with LC-ESI-MS (Figures 4, 5, and S7).

GenQ and GenB1 together catalyzed the conversion of G418 to JI-20B, with L-methionine as the most effective amino donor (80% conversion) (Figure 4 and S7Ai). Other amino donors tested except L-threonine and L were also accepted as substrates by GenB1, but the yield of JI-20B never exceeded 37% conversion. When GenB2 was used instead of GenB1, low level of conversion to JI-20B was observed when either L-Lys (7%), L-Met (9%) or L-Tyr (16%) was used as amino donor (Figure S7Aii). GenB3 also showed some activity (17% conversion), but only with L-Gln as the amino donor (Figure S7Aiii). GenB4, in contrast, did not show activity with any of the amino donors tested (Figure S7Aiv).

The combination of GenB1 and GenQ also catalyzed the conversion of gentamicin X2 into JI-20A, with L-Met and L-Orn as the only effective amino donors (54% and 70% conversion, respectively; Figure 5 and S7Av). GenB2 showed marginal activity (<5% conversion) on 6′-DOX, and only when L-Gln, L-Lys, L-Met, or L-Phe were used as the amino donors (Figure S7Avi). GenB3 accepted all amino donors tested with L-Orn giving ~50% conversion; Figure S7Avii). Gentamicin X2 was a very poor substrate (<6% conversion) for the combination of GenQ and GenB4 (Figure S7Aviii). When the aminotransferase activities of GenB1, GenB2, GenB 3, and GenB4 on 6′-DOG and 6′-DOX in the presence of L-methionine were compared, GenB1 clearly showed the highest activity, on both substrates (Figure 6). Although it cannot be ruled out that another amino acid donor is preferred in vivo, the robust activity seen with L-methionine in vitro suggests that this is likely to be the physiological substrate.

GenB2 Catalyzes In Vitro Epimerization of Gentamicin C2 and Gentamicin C2a

Given the evidence that the chief role of GenB2, GenB3, and GenB4 is in the unexplored dehydroxylation stage of gentamicin C biosynthesis, we investigated their possible involvement in the generation of gentamicin C2a, which like G418 and gentamicins C2 and C1 is methylated at the C-6′ position but which has the opposite configuration at this stereocenter (Figure 1). Purified recombinant GenB enzymes were assayed for their ability to catalyze the epimerization of gentamicin C2 into C2a, as described in the Experimental Procedures. GenB1, GenB3, and GenB4 showed no activity, as judged by LC-ESI-MS analysis of reaction mixtures. In contrast, GenB2 did catalyze the epimerization of C2, to form a mixture of C2 and C2a in a molar ratio of 1:2 (Figure S7B). Furthermore, when C2a was used as the substrate the reverse reaction was also detected and the final product mixture was found to contain the C2 and C2a epimers in the same molar ratio of 1:2 (Figure S7B), implying that equilibrium had been reached.

DISCUSSION

The results presented here significantly advance our understanding of the biosynthetic pathway to the gentamicin C complex, an established drug used to combat life-threatening infections. The task of assigning functions to individual gene products involved in the pathway is not trivial because the cluster contains multiple, mutually homologous genes for methyltransferases, oxidoreductases, and aminotransferases with potential for overlapping substrate specificities. There are numerous precedents from other aminoglycoside pathways for enzyme promiscuity or even dual function (Huang et al., 2005, 2007; Fan et al., 2008; Yokoyama et al., 2008; Park et al., 2011). From our analysis of the ΔgenQΔgenK mutant it is clear that GenK and GenQ uniquely control the fate of gentamicin X2 at the biosynthetic branch point. No other enzymes in *M. echinospora* were able to take over these roles under the fermentation conditions used. This is in contrast to (for example) the neomycin biosynthetic pathway, where the GenQ homolog Neo11 is proposed to function both as a 6′-paromamine dehydrogenase and then later in the pathway as the C-6‴-dehydrogenase acting on ring IV of neomycin (Llewellyn and Spencer, 2006; Huang et al., 2007; Kudo et al., 2009; Clausnitzer et al., 2011).

We have confirmed the important findings from several laboratories (Karki et al., 2012; Hong and Yan, 2012; Li et al., 2013) that the flux in the ΔgenK mutant is diverted exclusively down the "left-hand" branch (Figure 1) to C-6′-unmethylated

metabolites JI-20A, gentamicin C1a, and gentamicin C2b; and we have shown that chemical complementation of this mutant with G418 fully restores the flux down the "right-hand" branch (Figure 1) to wild-type levels. In contrast to the results with the ΔgenK mutant, the ΔgenQ mutant was unexpectedly found to be blocked in both branches of the pathway, accumulating both X2 and G418. This observation rules out any other dehydrogenase from the gentamicin cluster (such as GenD2 or GenD3) being capable of specific C-6′-dehydrogenation of G418. This observation also showed that it would not be possible by deletion of *genQ* to enhance selective production of those gentamicin C complex components synthesized via the "right-hand" branch.

We next turned our attention to the potential roles of the four genes predicted to encode Class III pyridoxal phosphate-dependent aminotransferases: *genB1*, *genB2*, *genB3*, and *genB4*. We anticipated that there might be considerable substrate overlap and promiscuity shown by these enzymes, and we therefore constructed and analyzed the complete combinatorial set of 15 mutants in which one, two, three, or all four of these genes had been independently deleted in-frame. LC-ESI-HRMS analysis of these mutant strains revealed several important insights into the enzymology of gentamicin biosynthesis.

First, the quadruple mutant ΔgenB4ΔgenB1ΔgenB3ΔgenB2 was unable to produce any intermediate more advanced than X2 and G418, which implies that one or more of these genes encodes an aminotransferase that partners GenQ. Analysis of the triple mutants in which only one of these four was active revealed a major role for GenB1 in partnering the GenQ dehydrogenase in the amination of both X2 and G418, but also revealed a low-level promiscuity of the other GenB enzymes that allows them partially to replace GenB1 in the amination steps.

The analysis of mutants lacking either *genB3* or *genB4* showed that biosynthesis of all members of the gentamicin C complex was blocked, with accumulation of the known intermediates JI-20A and JI-20B. GenB3 and GenB4 appear therefore to participate, perhaps as pyridoxal-dependent dehydratases, in the multistep removal of the two unactivated hydroxyl groups from one of the sugar rings. Deletion of *genB2* markedly lowered production of gentamicins C1a and C2b in the "left-hand" branch, perhaps through build-up of inhibitory metabolites; and blocked the production of C2 and C1, two of the three components of the gentamicin C complex that are produced in the "right-hand" branch of the pathway. The level of C2a in contrast rose over 7-fold, clearly implicating this stereoisomer as the first-formed product of didehydroxylation of JI-20B and identifying GenB2 as the catalyst for its epimerization to C2 (Figure 1).

We expressed and affinity-purified GenQ from recombinant *E. coli* as a soluble protein containing FAD. By using G418 as substrate for this enzyme, we have obtained LC-ESI-MS and MS-MS evidence for the production of the putative ketone intermediate 6′-DOG. Analogously, when GenQ was incubated with X2 as substrate the observed MS-MS fragmentation spectrum of the product was consistent with that expected for the predicted aldehyde intermediate 6′-DOX. We also expressed and purified GenB1, GenB2, GenB3, and GenB4 from recombinant *E. coli* as soluble enzymes containing pyridoxal phosphate, and this allowed us to reconstitute amination from X2 to JI-20A and from G418 to JI-20B respectively, by coupling the GenQ-catalyzed reaction to subsequent transamination catalyzed by one of these four enzymes. GenB1 proved far superior to the other three GenB enzymes in catalyzing these in vitro conversions, with L-methionine consistently the best amino donor among the many amino acids screened. These results are in good agreement with the data from our fermentation of GenB-deficient mutants (Table S1). From these results we conclude that GenB1 is the major, though not the exclusive, partner for GenQ in the dehydrogenation-transamination of both X2 and G418.

GenB3 and GenB4 are likely to cooperate with other enzymes in the 3′,4′-didehydroxylation of both JI-20A and JI-20B. Recent in vitro evidence using a substrate analog (Shao et al., 2013) implicates the phosphotransferase GenP in initiating this process, through phosphorylation at the C-3′ hydroxy group. Sisomicin, an antibiotic naturally produced by *M. inyoensis* (Reimann et al., 1974), is a 4′,5′-dehydro-derivative of gentamicin C1a. It has also been described as a minor component of the gentamicin fermentation of *M. echinospora* (Bérdy et al., 1977). When added to resting cells of mutant *M. echinospora*, *M. sagamiensis* (Kase et al., 1982b), or *M. rhodorangea* (Lee et al., 1977) blocked early in the gentamicin pathway, sisomicin is reportedly converted into gentamicin C1a and C2b. Analogously, verdamicin C2a (4′,5′-dehydro-gentamicin C2a) is an antibiotic naturally produced by *M. grisea* (Weinstein et al., 1975), which reportedly can be bioconverted by an early stage blocked mutant of *M. sagamiensis* (Kase et al., 1982b) into gentamicins C2a, C2, and C1. It remains to be determined how closely sisomicin and verdamicin are related to genuine intermediates in the normal 3′,4′-didehydroxylation pathway that leads to gentamicins.

A further open question has been the origin of gentamicin C2a, the minor C-6′ epimer of C2. A blocked mutant of *M. sagamiensis* (KY 11564) has been described (Kase et al., 1982a), which accumulates gentamicin C2a but neither C2 nor C1. Another mutant that appears to be blocked earlier in the pathway bioconverts verdamicin C2a into gentamicin C2a then into C2 and C1 (Kase et al., 1982b). From the results obtained here from fermentation of the ΔgenB2 mutant, it is clear that this gene is required for formation of C2 but not for C2a. We investigated a possible role for GenB1, GenB2, GenB3, or GenB4 in interconverting gentamicins C2 and C2a. Remarkably, purified GenB2 (but not the others) proved competent in vitro to catalyze the 6′-epimerization of gentamicin C2a into gentamicin C2 (and vice versa). This evidence strongly supports the original proposal (Kase et al., 1982b) that C2a is an obligatory precursor to C2, which in turn implies that two inversions of the configuration at the C-6′ position occur during the transformation of G418 into gentamicin C2.

SIGNIFICANCE

Aminoglycosides, mainly produced by actinobacteria, constitute a widespread and versatile group of bioactive natural products. Examples of clinically used aminoglycosides include gentamicin to treat neonatal sepsis; kanamycin and amikacin to treat MRSA; and paromomycin to treat visceral leishmaniasis. Aminoglycosides are also under active investigation for their anti-HIV properties and in attempts to rescue certain genetic conditions by allowing

readthrough of premature stop codons. Gentamicin in particular is of continuing interest despite its known toxicity because of its remarkable potency in combating systemic Gram-negative infections. The perspective of a safer and less expensive gentamicin through administration of a single component, or using a single component for semisynthesis of a novel derivative, is therefore very attractive. The work we report here has used complementary in vivo and in vitro approaches to identify the key enzymes that control the branch point in the gentamicin biosynthetic pathway. It has also revealed the involvement of the pyridoxal phosphate-dependent enzymes GenB2, GenB3, and GenB4 in the subsequent steps. By more closely defining the molecular enzymology of the pathway in *Micromonospora echinospora*, this work has brought closer the goal of assembling a defined set of pathway enzymes that might in future deliver single gentamicin C components in a scaleable and sustainable synthetic biology process.

EXPERIMENTAL PROCEDURES

Bacterial Strains, Chemicals, and Culture Conditions

E. coli DH10B and NovaBlue strains were used as cloning hosts, and *E. coli* BL21(DE3) for protein expression. *E. coli* ET12567/pUZ8002 was used for intergeneric conjugation between *E. coli* and *Streptomyces* (MacNeil et al., 1992). *Streptomyces fradiae* NCIMB 8233 (Waksman and Lechevalier, 1949) was the source of template DNA for cloning the *neo11* gene. *M. echinospora* ATCC15835 (Weinstein et al., 1963) was used for creation of in-frame deletion mutants and as the source of *gen* genes. Restriction endonucleases, Phusion High-Fidelity Master Mix with GC-buffer, and T4 DNA ligase were from NEB England Biolabs. *Pfu* DNA polymerase was from Fermentas. Oligonucleotide primers were synthesized by either GenScript or Invitrogen. DNA sequencing of PCR products was performed by GenScript or by the Department of Biochemistry DNA Sequencing Facility, University of Cambridge. DIG DNA labeling and detection kits were from Roche. G418 used for feeding studies was obtained from GIBCO. For in vitro enzyme assays, G418 and amino acids were from Sigma-Aldrich, gentamicin X2 was from Toku-E, and *S*-adenosyl methionine was from Carbosynth.

M. echinospora ATCC15835 wild-type and mutants were grown in ATCC Medium 172 (glucose 10 g, soluble starch 20 g, yeast extract 5 g, N-Z amine type A 5 g, $CaCO_3$ 1 g, 1 l distilled water) for chromosomal DNA isolation and preparation of mycelium. *E. coli* strains were maintained in 2 × TY or LB media at 37°C with the appropriate antibiotic selection at a final concentration of 25 μg/ml apramycin, 100 μg/ml, carbenicillin, 25 μg/ml chloramphenicol, 25 μg/ml or 50 μg/ml kanamycin, 15 μg/ml tetracycline, or 25 μg/ml thiostrepton.

For production of gentamicin C complex and intermediates, *M. echinospora* ATCC15835 and its mutants were cultured in two stages. A seed culture was maintained in liquid ATCC 172 medium at 28°C with shaking at 220 rpm for 2 days before being inoculated into fresh liquid ATCC 172 Medium. The production fermentation was incubated at 28°C with shaking for 8 days. For feeding experiments, filter-sterilized compounds (50 μg/ml) were added to the medium just before addition of the seed culture.

Construction of Gene Disruption Plasmids

For in-frame deletion, DNA fragments flanking each target gene were amplified from the genomic DNA of *M. echinospora* ATCC15835 (see List of Primers Used in the Supplemental Information). The PCR products were each cloned into pUC18, then cut out and cloned together into the *Streptomyces-E. coli* shuttle vector pYH7 (Sun et al., 2009) to obtain the following gene disruption plasmids: pYH286 (for ΔgenQ), pWHU1 (for ΔgenK), pWHU4 (for ΔgenB1), pWHU2 (for ΔgenB2), pWHU5 (for ΔgenB3), and pWHU3 (for ΔgenB4; Table S2). For generation of the ΔgenB4ΔgenB3 double mutant, DNA fragments flanking the *genB3* gene in the *genB4* mutant were amplified using primer pairs gen4-genB3-L1/L2 and genB4-genB3-R1/R2 (see List of Primers Used in the Supplemental Information) and inserted into vector pYH7 to yield plasmid pWHU43 (Tables S2 and S3). All plasmids were verified by sequencing.

Targeted In-Frame Gene Deletion

To create individual in-frame deletion mutants of *genQ*, *genK*, *genB1*, *genB2*, *genB3* and *genB4*, the corresponding plasmid pYH286, pWHU1, pWHU4, pWHU2, pWHU5, and pWHU3 was introduced into the wild-type strain by conjugation from *E. coli* ET12567/pUZ8002 (MacNeil et al., 1992). Apramycin-sensitive (Apr^S) colonies were counterselected from the initial Apr^R exconjugants after nonselective growth. The Apr^S exconjugants were confirmed by PCR amplification with appropriate checking primers and by Southern hybridization (Figures S2 and S4B). ΔgenQΔgenK was obtained by disruption of the *genK* gene in the ΔgenQ mutant using plasmid pWHU1 (Figure S2 and Tables S2 and S3). Similarly, the double-, triple-, and quadruple-in-frame deletion mutants of *genB* genes in all combinations were created by disruption of target gene(s) in appropriate mutants (Figure S4B and Table S3).

Complementation of the ΔgenQ and ΔgenK Mutants

Complementation plasmids were prepared by cloning *genQ*, *genK* and *neo11*, respectively, into pWHU77 (a plasmid derived from pIB139 (Wilkinson et al., 2002; Del Vecchio et al., 2003) with the apramycin resistance gene replaced by a thiostrepton resistance gene) under the control of the P*ermE** promoter (Figure S2(4)D). The *genQ* and *genK* were amplified from the genomic DNA of *M. echinospora* ATCC15835 using primer pairs genQ-EP1/genQ-EP2 and genK-EP1/genK-EP2 (see List of Primers Used in the Supplemental Information), respectively. Primers neo11-EP1 and neo11-EP2 were used to amplify *neo11* from *S. fradiae* NCIMB8233. The PCR products were inserted into pWHU77 between the *Nde*I and EcoRI sites to give pWHU163 (*genQ*), pWHU165 (*neo11*), and pWHU67 (*genK*; Table S2). After sequence confirmation, these plasmids were introduced individually into ΔgenQ (pWHU163 and pWHU165) and ΔgenK (pWHU67) by conjugation. Complemented exconjugants were verified based on thiostrepton resistance and confirmed by PCR (Figures S2(4)A to ∼S2(4)C).

Extraction and LC-ESI-HRMS Analysis of Gentamicin C Complex and Intermediates

Cultures of wild-type and mutant strains of *M. echinospora* ATCC15835 were adjusted to pH 2.0 with HCl and the acidified broth was agitated overnight on a laboratory rocker. The clarified supernatant after centrifugation was filtered through Whatman filter paper, applied to a column of DOWEX 50 WX8-200 ion-exchange resin (2 g), preconditioned with 50 ml acetonitrile followed by 50 ml MilliQ water, and equilibrated with 50 ml of 10 mM HCl. The column was washed with 30 ml of 10 mM HCl and eluted with 10 ml of 1 M NH_4OH. The eluate was freeze-dried, redissolved in 1 ml MilliQ water, and filtered through 0.22 μm microporous membrane before subjection to LC-ESI-HRMS analysis.

Cloning of genQ, genB1, genB2, genB3, and genB4 Genes for Expression in *E. coli*

Genes *genQ*, *genB1*, *genB2*, *genB3*, and *genB4* were amplified from the genomic DNA of *M. echinospora* ATCC15835 by PCR (see List of Primers Used in Supplemental Information). PCR was carried out using *Pfu* DNA polymerase with 25 cycles of denaturation at 94°C for 1 min, annealing at 60°C for 1 min, and extension at 72°C for 2 min plus final extension at 72°C for 10 min. The PCR products were digested with appropriate restriction enzymes, purified by gel extraction (Fermentas), and inserted into plasmid pET28a(+). The resulting constructs (Table S2) were verified by DNA sequencing.

Overexpression and Purification of Recombinant Proteins

E. coli BL21(DE3) cells containing the recombinant plasmids were cultured in LB broth containing kanamycin (50 μg/ml) at 37°C to absorption at 600 nm of 0.5 to ∼0.8 and expression induced by isopropylthiogalactoside (0.1 mM) at 16°C with shaking overnight. Cells were harvested and resuspended in binding buffer (0.5 M NaCl, 20 mM Tris-HCl [pH 7.9]). The recombinant protein was released by sonication for 4 min using a 2 s on/6 s off cycle. Clarified cell lysate was passed through a column of Co^{2+}-charged His-Bind resin (GE Healthcare). After washing the column with washing buffer (0.5 M NaCl, 5-60 mM imidazole, 20 mM Tris-HCl [pH 7.9]). N-His_6-tagged recombinant proteins

were eluted with elution buffer (0.5 M NaCl, 250 mM imidazole, 20 mM Tris-HCl [pH 7.9]). Imidazole in the eluted protein solutions was removed by buffer exchange using Amicon Ultracentrifugal filters (Millipore). The purified proteins were stored at $-20^\circ C$ in 250 mM NaCl, 10 mM Tris-HCl, pH 7.9, and 50% glycerol. The identities of the purified proteins were confirmed by SDS-PAGE, UV-Vis absorbance analysis and LC-ESI-MS (Thermo Finnigan). Protein concentrations were determined using Bradford protein dye reagent (Sigma).

Enzyme Assays

Assay mixtures for GenQ (50 μl) contained substrate (200 μM) and purified GenQ (16 μM) in Tris-HCl buffer (50 mM, pH 8.0). Coupled assays with GenQ and GenB (B = B1, B2, B3, or B4) contained substrate (200 μM), amino donor (2 mM), GenQ (16 μM), and GenB (20 μM) in 50 μl Tris-HCl buffer (50 mM, pH 8.0). The isomerase activity assays contained substrate (C2 or C2a, 200 μM) and each one of the four GenB enzymes (20 μM), respectively, in 50 μl Tris-HCl buffer (50 mM, pH 8.0). These high concentrations of each enzyme were chosen based on previous work with the GenQ homolog Neo11 (Huang et al., 2007) where the enzyme was found to be unstable during incubations. Incubations were at 30°C overnight and quenched by addition of chloroform (100 μl) followed by vortexing and centrifugation to remove protein. The supernatants were analyzed by LC-ESI-MS.

LC-ESI-MS Analyses

LC-ESI-HRMS analysis of extracts of wild-type and mutant strains of *M. echinospora* ATCC15835 was performed on a Thermo Electron LTQ-Orbitrap XL fitted with a Phenomenex Luna C18 column at a flow rate of 0.4 ml/min using a mobile phase of (A) 0.2% trifluoroacetic acid (TFA) in H_2O (adjusted to pH 2.0 with NH_4OH) and (B) 100% CH_3CN; the gradient for separation of both gentamicin C complex and related intermediates: 0–18 min 2% B to 14% B, 18–19 min 14% B to 40% B, 19–24 min 40% B, 24–25 min 40% B to 2% B, 25–30 min 2% B. The gradient for separation of gentamicin C complex: 0–14 min 2% B to 6% B, 14–16 min 6% B to 8% B, 16–25 min 8% B-15% B, 25–26 min 15% B to 40% B, 26–34 min 40% B, 34–35 min 40% B to 2% B, 35–45 min 2% B. LC-ESI-MS analysis of assay mixtures and proteins was carried out on a Thermo Finnigan LCQ connected to an Agilent HP 1100 HPLC system. Deproteinated assay mixtures were separated on a Prodigy C18 column (Phenomenex) at a 0.4 ml/min flow rate using a mobile phase of (A) 0.3% TFA in H_2O and (B) 0.1% TFA in CH_3CN; 98% A for 10 min, gradient to 90% B over 15 min, then 90% B for 5 min followed by a gradient from 90% B to 2% B. MS/MS analyses were carried out in the positive ionization mode with 35% relative collision energy. Aminoglycosides were quantified by integration of areas under peaks. For proteins, a Nucleosil C4 column (250 × 2 mm, 5 μm, Macherery-Nagel) was used at a flow rate of 0.2 ml/min with the following conditions: mobile phase (A) H_2O 0.1% TFA and (B) CH_3CN 0.1% TFA; 35% B to 45% B over 5 min, increase the gradient of B to 75% over 20 min, 95% B for 2 min, 95% B for 7 min, followed by a gradient from 95% B to 35%B over 3 min.

SUPPLEMENTAL INFORMATION

Supplemental Information includes Supplemental Experimental Procedures, seven figures, and three tables and can be found with this article online at http://dx.doi.org/10.1016/j.chembiol.2014.03.005.

AUTHOR CONTRIBUTIONS

F.H., P.F.L., and Y.S. conceived the experiments; J.G., C.H., X.D., and X.J. constructed and analyzed mutants; F.H. carried out in vitro analyses; J.G., F.H., C.H., F.J.L., Z.D., P.F.L., and Y.S. analyzed the results; and P.F.L. and Y.S. wrote the paper.

ACKNOWLEDGMENTS

This work was supported by a grant from the Medical Research Council, UK (G1001687) to P.F.L. and by the 973 and 863 programs from the Ministry of Science and Technology of China, and Translational Medical Research Fund of Wuhan University School of Medicine to Y.S. We thank Dr. Ian Garrard (Brunel University) for his expert help with purification of gentamicin C components by dynamic countercurrent chromatography.

Received: January 16, 2014
Revised: March 12, 2014
Accepted: March 14, 2014
Published: April 17, 2014

REFERENCES

Aboshanab, K.M.A. (2005). Genetic studies on the biosynthesis of the major aminoglycoside antibiotics: isolation, analysis and comparison of the biosynthetic gene clusters for 12 aminoglycoside antibiotics. PhD thesis. (Wuppertal, Germany: Bergische Universität).

Bérdy, J., Pauncz, J.K., Vajna, Z.M., Horváth, G., Gyimesi, J., and Koczka, I. (1977). Metabolites of gentamicin-producing Micromonospora species I. Isolation and identification of metabolites. J. Antibiot. *30*, 945–954.

Bockenhauer, D., Hug, M.J., and Kleta, R. (2009). Cystic fibrosis, aminoglycoside treatment and acute renal failure: the not so gentle micin. Pediatr. Nephrol. *24*, 925–928.

Clausnitzer, D., Piepersberg, W., and Wehmeier, U.F. (2011). The oxidoreductases LivQ and NeoQ are responsible for the different 6′-modifications in the aminoglycosides lividomycin and neomycin. J. Appl. Microbiol. *111*, 642–651.

Cuccarese, M.F., Singh, A., Amiji, M., and O'Doherty, G.A. (2013). A novel use of gentamicin in the ROS-mediated sensitization of NCI-H460 lung cancer cells to various anticancer agents. ACS Chem. Biol. *8*, 2771–2777.

Del Vecchio, F., Petkovic, H., Kendrew, S.G., Low, L., Wilkinson, B., Lill, R., Cortés, J., Rudd, B.A.M., Staunton, J., and Leadlay, P.F. (2003). Active-site residue, domain and module swaps in modular polyketide synthases. J. Ind. Microbiol. Biotechnol. *30*, 489–494.

Fan, Q.Z., Huang, F.L., Leadlay, P.F., and Spencer, J.B. (2008). The neomycin biosynthetic gene cluster of *Streptomyces fradiae* NCIMB 8233: genetic and biochemical evidence for the roles of two glycosyltransferases and a deacetylase. Org. Biomol. Chem. *6*, 3306–3314.

Hainrichson, M., Nudelman, I., and Baasov, T. (2008). Designer aminoglycosides: the race to develop improved antibiotics and compounds for the treatment of human genetic diseases. Org. Biomol. Chem. *6*, 227–239.

Hong, W.R., and Yan, L.B. (2012). Identification of *gntK*, a gene required for the methylation of purpurosamine C-6′ in gentamicin biosynthesis. J. Gen. Appl. Microbiol. *58*, 349–356.

Hong, W.R., Ge, M., Zeng, Z.H., Zhu, L., Luo, M.Y., Shao, L., and Chen, D.J. (2009). Molecular cloning and sequence analysis of the sisomicin biosynthetic gene cluster from *Micromonospora inyoensis*. Biotechnol. Lett. *31*, 449–455.

Houghton, J.L., Green, K.D., Chen, W., and Garneau-Tsodikova, S. (2010). The future of aminoglycosides: the end or renaissance? ChemBioChem *11*, 880–902.

Huang, F.L., Haydock, S.F., Mironenko, T., Spiteller, D., Li, Y., and Spencer, J.B. (2005). The neomycin biosynthetic gene cluster of *Streptomyces fradiae* NCIMB 8233: characterisation of an aminotransferase involved in the formation of 2-deoxystreptamine. Org. Biomol. Chem. *3*, 1410–1418.

Huang, F.L., Spiteller, D., Koorbanally, N.A., Li, Y., Llewellyn, N.M., and Spencer, J.B. (2007). Elaboration of neosamine rings in the biosynthesis of neomycin and butirosin. ChemBioChem *8*, 283–288.

Karki, S., Kim, J.Y., Park, S.H., and Kwon, H.J. (2012). Gene inactivation study on *gntK*, a putative C-methyltransferase gene in gentamicin biosynthesis from *Micromonospora echinospora*. J. Kor. Soc. Appl. Biol. Chem. *55*, 439–442.

Kase, H., Odakura, Y., and Nakayama, K. (1982a). Sagamicin and the related aminoglycosides: fermentation and biosynthesis. I. Biosynthetic studies with the blocked mutants of *Micromonospora sagamiensis*. J. Antibiot. *35*, 1–9.

Kase, H., Shimura, G., Iida, T., and Nakayama, K. (1982b). Biotransformation of sisomicin and verdamicin by *Micromonospora sagamiensis*. Agric. Biol. Chem. *46*, 515–522.

Kharel, M.K., Basnet, D.B., Lee, H.C., Liou, K., Woo, J.S., Kim, B.G., and Sohng, J.K. (2004a). Isolation and characterization of the tobramycin biosynthetic gene cluster from *Streptomyces tenebrarius*. FEMS Microbiol. Lett. *230*, 185–190.

Kharel, M.K., Basnet, D.B., Lee, H.C., Liou, K., Moon, Y.H., Kim, J.J., Woo, J.S., and Sohng, J.K. (2004b). Molecular cloning and characterization of a 2-deoxystreptamine biosynthetic gene cluster in gentamicin-producing *Micromonospora echinospora* ATCC15835. Mol. Cells *18*, 71–78.

Kharel, M.K., Subba, B., Basnet, D.B., Woo, J.S., Lee, H.C., Liou, K., and Sohng, J.K. (2004c). A gene cluster for biosynthesis of kanamycin from *Streptomyces kanamyceticus*: comparison with gentamicin biosynthetic gene cluster. Arch. Biochem. Biophys. *429*, 204–214.

Kim, J.Y., Suh, J.W., Kang, S.H., Phan, T.H., Park, S.H., and Kwon, H.J. (2008). Gene inactivation study of *gntE* reveals its role in the first step of pseudotrisaccharide modifications in gentamicin biosynthesis. Biochem. Biophys. Res. Commun. *372*, 730–734.

Kim, H.J., McCarty, R.M., Ogasawara, Y., Liu, Y.N., Mansoorabadi, S.O., LeVieux, J., and Liu, H.W. (2013). GenK-catalyzed C-6′ methylation in the biosynthesis of gentamicin: isolation and characterization of a cobalamin-dependent radical SAM enzyme. J. Am. Chem. Soc. *135*, 8093–8096.

Kobayashi, M., Sone, M., Umemura, M., Nabeshima, T., Nakashima, T., and Hellström, S. (2008). Comparisons of cochleotoxicity among three gentamicin compounds following intratympanic application. Acta Otolaryngol. *128*, 245–249.

Kudo, F., and Eguchi, T. (2009). Biosynthetic genes for aminoglycoside antibiotics. J. Antibiot. *62*, 471–481.

Kudo, F., Sucipto, H., and Eguchi, T. (2009). Enzymatic activity of a glycosyltransferase KanM2 encoded in the kanamycin biosynthetic gene cluster. J. Antibiot. *62*, 707–710.

Lee, B.K., Bailey, J.V., Condon, R.G., Marquez, J.A., Wagman, G.H., and Weinstein, M.J. (1977). Biotransformation of sisomicin to gentamicin C2b. Antimicrob. Agents Chemother. *12*, 335–338.

Li, D., Li, H., Ni, X., Zhang, H., and Xia, H.Z. (2013). Construction of a gentamicin C1a-overproducing strain of *Micromonospora purpurea* by inactivation of the *gacD* gene. Microbiol. Res. *168*, 263–267.

Linde, L., and Kerem, B. (2008). Introducing sense into nonsense in treatments of human genetic diseases. Trends Genet. *24*, 552–563.

Llewellyn, N.M., and Spencer, J.B. (2006). Biosynthesis of 2-deoxystreptamine-containing aminoglycoside antibiotics. Nat. Prod. Rep. *23*, 864–874.

MacNeil, D.J., Occi, J.L., Gewain, K.M., MacNeil, T., Gibbons, P.H., Ruby, C.L., and Danis, S.J. (1992). Complex organization of the *Streptomyces avermitilis* genes encoding the avermectin polyketide synthase. Gene *115*, 119–125.

Missiakas, D., Georgopoulos, C., and Raina, S. (1993). The *Escherichia coli* heat shock gene *htpY*: mutational analysis, cloning, sequencing, and transcriptional regulation. J. Bacteriol. *175*, 2613–2624.

Ota, Y., Tamegai, H., Kudo, F., Kuriki, H., Koike-Takeshita, A., Eguchi, T., and Kakinuma, K. (2000). Butirosin-biosynthetic gene cluster from *Bacillus circulans*. J. Antibiot. *53*, 1158–1167.

Park, J.W., Hong, J.S., Parajuli, N., Jung, W.S., Park, S.R., Lim, S.K., Sohng, J.K., and Yoon, Y.J. (2008). Genetic dissection of the biosynthetic route to gentamicin A2 by heterologous expression of its minimal gene set. Proc. Natl. Acad. Sci. USA *105*, 8399–8404.

Park, J.W., Park, S.R., Nepal, K.K., Han, A.R., Ban, Y.H., Yoo, Y.J., Kim, E.J., Kim, E.M., Kim, D., Sohng, J.K., and Yoon, Y.J. (2011). Discovery of parallel pathways of kanamycin biosynthesis allows antibiotic manipulation. Nat. Chem. Biol. *7*, 843–852.

Park, S.R., Park, J.W., Ban, Y.H., Sohng, J.K., and Yoon, Y.J. (2013). 2-Deoxystreptamine-containing aminoglycoside antibiotics: recent advances in the characterization and manipulation of their biosynthetic pathways. Nat. Prod. Rep. *30*, 11–20.

Reimann, H., Cooper, D.J., Mallams, A.K., Jaret, R.S., Yehaskel, A., Kugelman, M., Vernay, H.F., and Schumacher, D. (1974). The structure of sisomicin, a novel unsaturated aminocyclitol antibiotic from *Micromonospora inyoensis*. J. Org. Chem. *39*, 1451–1457.

Sandoval, R.M., Reilly, J.P., Running, W., Campos, S.B., Santos, J.R., Phillips, C.L., and Molitoris, B.A. (2006). A non-nephrotoxic gentamicin congener that retains antimicrobial efficacy. J. Am. Soc. Nephrol. *17*, 2697–2705.

Shao, L., Chen, J.S., Wang, C.X., Li, J.A., Tang, Y.M., Chen, D.J., and Liu, W. (2013). Characterization of a key aminoglycoside phosphotransferase in gentamicin biosynthesis. Bioorg. Med. Chem. Lett. *23*, 1438–1441.

Subba, B., Kharel, M.K., Lee, H.C., Liou, K., Kim, B.G., and Sohng, J.K. (2005). The ribostamycin biosynthetic gene cluster in *Streptomyces ribosidificus*: comparison with butirosin biosynthesis. Mol. Cells *20*, 90–96.

Sun, Y.H., He, X.Y., Liang, J.D., Zhou, X.F., and Deng, Z.X. (2009). Analysis of functions in plasmid pHZ1358 influencing its genetic and structural stability in *Streptomyces lividans* 1326. Appl. Microbiol. Biotechnol. *82*, 303–310.

Testa, R.T., and Tilley, B.C. (1975). Biotransformation, a new approach to aminoglycoside biosynthesis. I. Sisomicin. J. Antibiot. *28*, 573–579.

Testa, R.T., and Tilley, B.C. (1976). Biotransformation, a new approach to aminoglycoside biosynthesis: II. Gentamicin. J. Antibiot. *29*, 140–146.

Thibodeaux, C.J., Melançon, C.E., 3rd, and Liu, H.W. (2008). Natural-product sugar biosynthesis and enzymatic glycodiversification. Angew. Chem. Int. Ed. Engl. *47*, 9814–9859.

Unwin, J., Standage, S., Alexander, D., Hosted, T., Jr., Horan, A.C., and Wellington, E.M. (2004). Gene cluster in *Micromonospora echinospora* ATCC15835 for the biosynthesis of the gentamicin C complex. J. Antibiot. *57*, 436–445.

Waksman, S.A., and Lechevalier, H.A. (1949). Neomycin, a new antibiotic active against Streptomycin-resistant bacteria, including tuberculosis organisms. Science *109*, 305–307.

Weinstein, M.J., Luedemann, G.M., Oden, E.M., and Wagman, G.H. (1963). Gentamicin, a new broad-spectrum antibiotic complex. Antimicrob Agents Chemother (Bethesda) *161*, 1–7.

Weinstein, M.J., Wagman, G.H., Marquez, J.A., Testa, R.T., and Waitz, J.A. (1975). Verdamicin, a new broad spectrum aminoglycoside antibiotic. Antimicrob. Agents Chemother. *7*, 246–249.

Wilkinson, C.J., Hughes-Thomas, Z.A., Martin, C.J., Böhm, I., Mironenko, T., Deacon, M., Wheatcroft, M., Wirtz, G., Staunton, J., and Leadlay, P.F. (2002). Increasing the efficiency of heterologous promoters in actinomycetes. J. Mol. Microbiol. Biotechnol. *4*, 417–426.

Yokoyama, K., Yamamoto, Y., Kudo, F., and Eguchi, T. (2008). Involvement of two distinct N-acetylglucosaminyltransferases and a dual-function deacetylase in neomycin biosynthesis. ChemBioChem *9*, 865–869.

Zhang, Q., van der Donk, W.A., and Liu, W. (2012). Radical-mediated enzymatic methylation: a tale of two SAMS. Acc. Chem. Res. *45*, 555–564.

Chemistry & Biology, Volume *21*

Supplemental Information

Specificity and Promiscuity at the Branch Point in Gentamicin Biosynthesis

Junhong Guo, Fanglu Huang, Chuan Huang, Xiaobo Duan, Xinyun Jian, Finian Leeper, Zixin Deng, Peter F. Leadlay, and Yuhui Sun

SUPPLEMENTAL INFORMATION

<u>Inventory of Supplemental Information</u>

Figure S1 Sequence alignment of GenK and GenQ with their homologs in other aminoglycoside biosynthetic pathways, related to Figure 2

Figure S2 Schematic representation and confirmation of in-frame deletion of *genK* and *genQ*, and complementation of ΔgenK and ΔgenQ related to Figure 2

Figure S3 LC-HRMS analysis of production of gentamicin C complex and intermediates by the wild type, ΔgenK, ΔgenQ, and ΔgenQΔgenK mutants of *M. echinospora* ATCC15835, related to Figure 2

Figure S4 (A) Sequence alignment of GenB1, GenB2, GenB3 and GenB4 with their homolog Neo18 from neomycin biosynthetic pathway; (B) Schematic representation and confirmation of in-frame deletion of *genB1, genB2, genB3 and genB4,* related to Figure 3

Figure S5 Confirmation of C2a production in ΔgenB2 and ΔgenB2ΔgenB1 mutants, related to Figure 3

Figure S6 Cloning and characterization of GenQ, GenB1, GenB2, GenB3 and GenB4. (A) Cloning of *genQ* with alternative start codon; (B) SDS-PAGE gel of VM_GenQ and GenB; (C) UV-Vis. Spectra of VM_GenQ and FAD; (D) UV-Vis spectra of GenB, related to Figures 4, 5 and 6

Figure S7 LC-MS analysis of activity assays of GenB1, GenB2, GenB3 and GenB4, related to Figures 4 and 5

List of oligonucleotide primers used in this study, related to Experimental Procedures

Table S1 LC-HRMS analysis of gentamicin C complex and intermediates production by the wild type and *genB* mutants of *M. echinospora* ATCC15835, related to Figure 3

Table S2 Plasmids used in this study, related to Experimental Procedures

Table S3 In-frame deletion mutants and complemented strains used in this study, related to Experimental Procedures

Figure S1, related to Figure 2. Sequence alignment of GenK (A) with homologous radical SAM-dependent methyltransferases and of GenQ (B) with homologous oxidoreductases from other aminoglycoside biosynthetic pathways

Black letters in gray boxes represent amino acid residues identical in all species, while white letters in gray boxes represent similar amino acid residues. The red box in **A** indicates the consensus CX_3CX_2C binding motif for the FeS cluster.

A

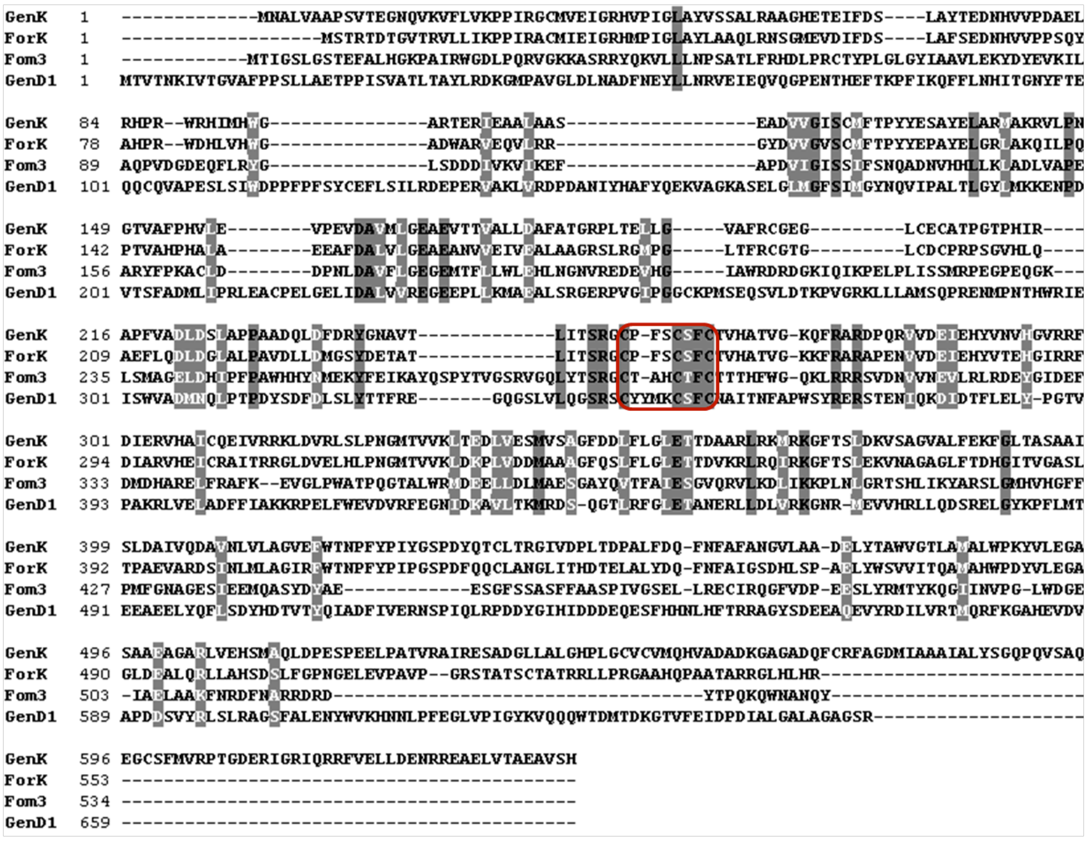

ForK (accession no. CAF31547.1): from *Micromonospora olivasterospora*;

Fom3 (accession no. ACG70826.1): from *Streptomyces fradiae*;

GenD1 (accession no. CAF31434.1): from *Micromonospora echinospora*.

B

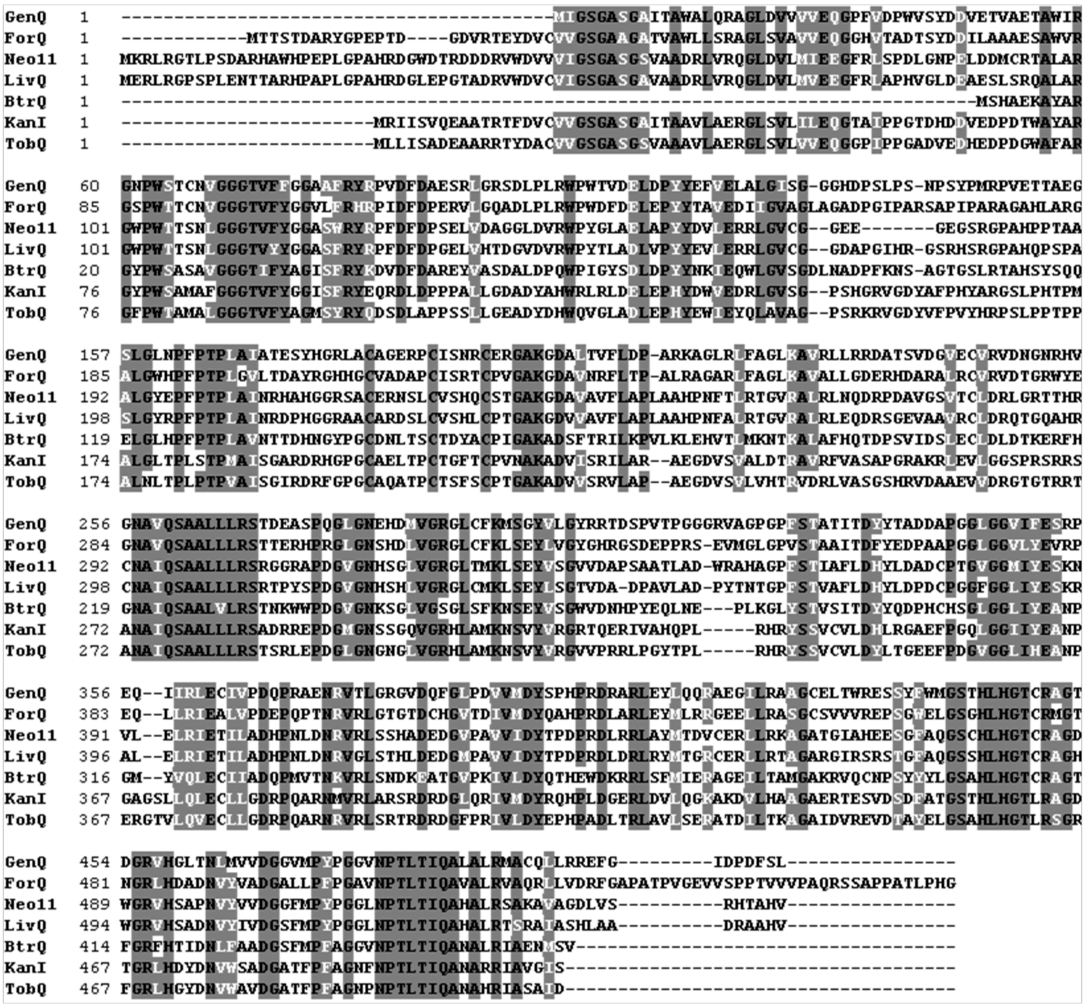

Neo11 (accession no. CAH58694.1): from *S. fradiae*;

BtrQ (accession no. BAE07079.1): from *Bacillus circulans*;

LivQ (accession no. CAG38701.1): from *S. lividus*;

KanI (accession no. CAF60534.1): from *S. kanamyceticus*;

TobQ (accession no. CAH18553.1): from *Streptoalloteichus tenebrarius*;

ForQ (accession no. CAF31536.1): from *M. olivasterospora*.

Figure S2, related to Figure 2. In-frame deletion of *genK* and *genQ* in *M. echinospora* ATCC15835*,* and complementation of ΔgenK and ΔgenQ
(A) Schematic representation of the in-frame deletions; (B) Confirmation by Southern blot; (C) Confirmation by PCR. The arrows indicate the expected size of the PCR fragments in the wild type and mutants. The dash arrows show the location of PCR primers for check mutants.

(1) ΔgenK

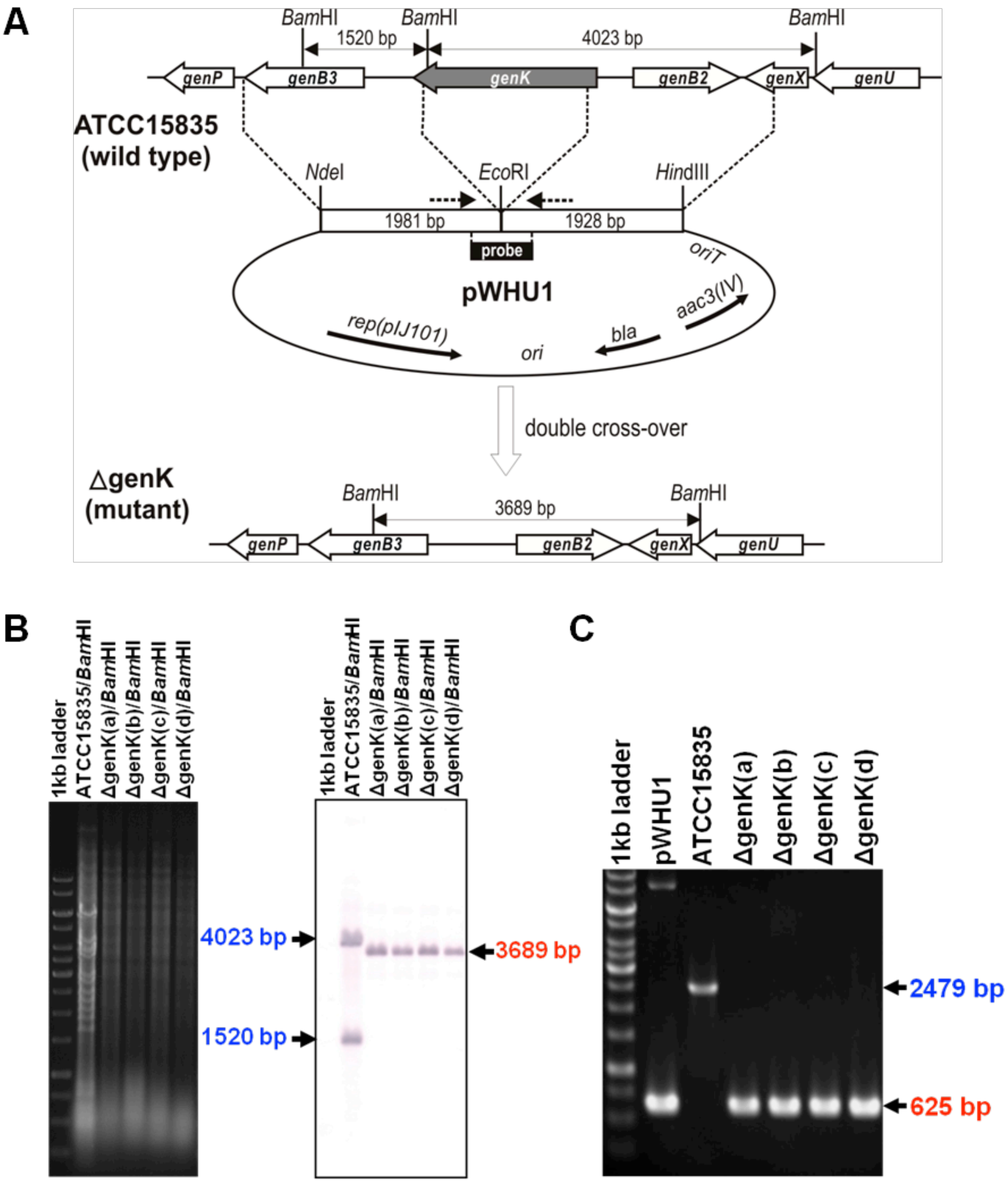

(2) ΔgenQ

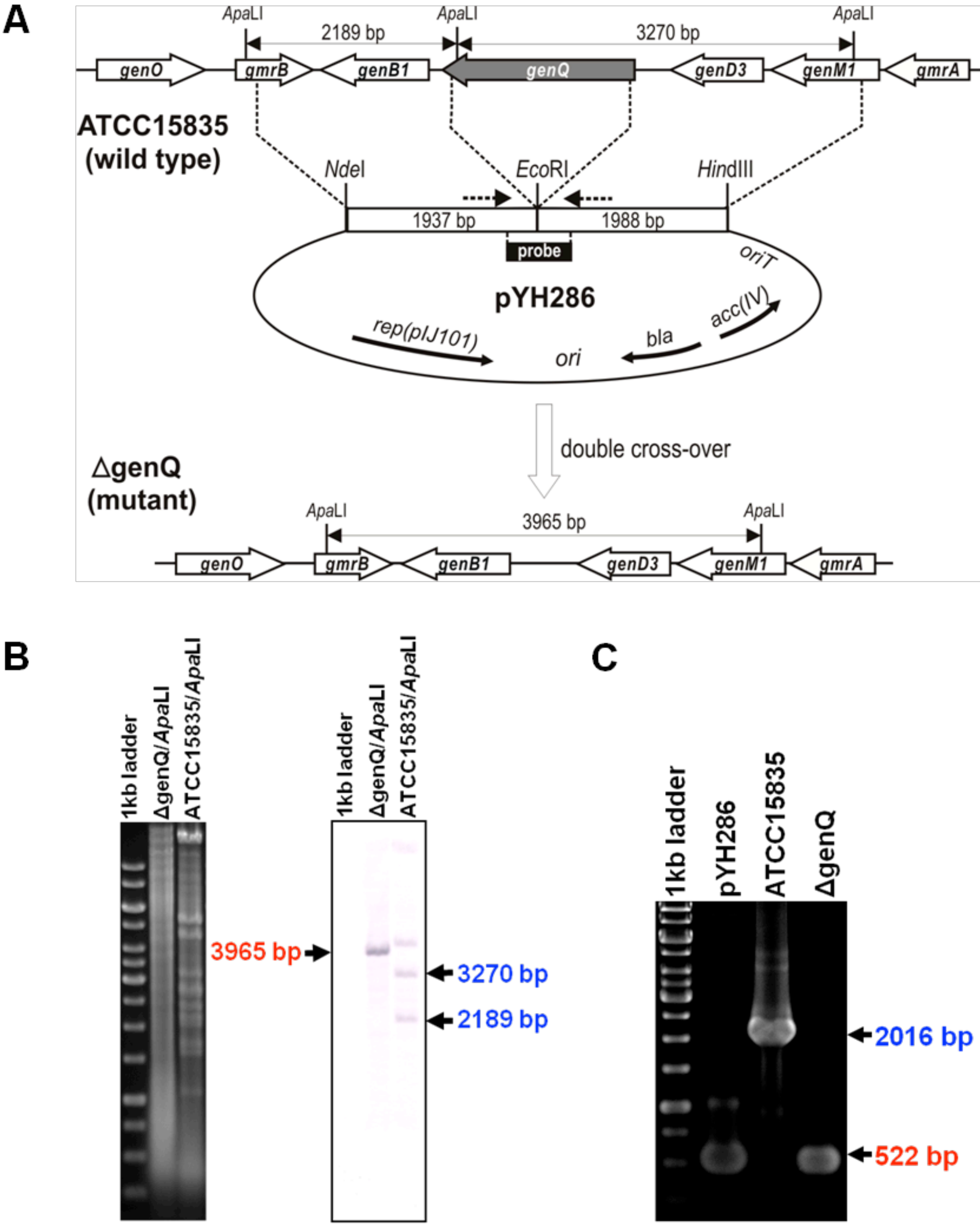
A
ApaLI
2189 bp
ApaLI
3270 bp
ApaLI
genO
gmrB
genB1
genQ
genD3
genM1
gmrA
ATCC15835
(wild type)
NdeI
EcoRI
HindIII
1937 bp
1988 bp
probe
oriT
pYH286
acc(IV)
rep(pIJ101)
bla
ori
double cross-over
ΔgenQ
(mutant)
ApaLI
3965 bp
ApaLI
genO
gmrB
genB1
genD3
genM1
gmrA
B
1kb ladder
ΔgenQ/ApaLI
ATCC15835/ApaLI
3965 bp
3270 bp
2189 bp
C
1kb ladder
pYH286
ATCC15835
ΔgenQ
2016 bp
522 bp

(3) ΔgenQΔgenK

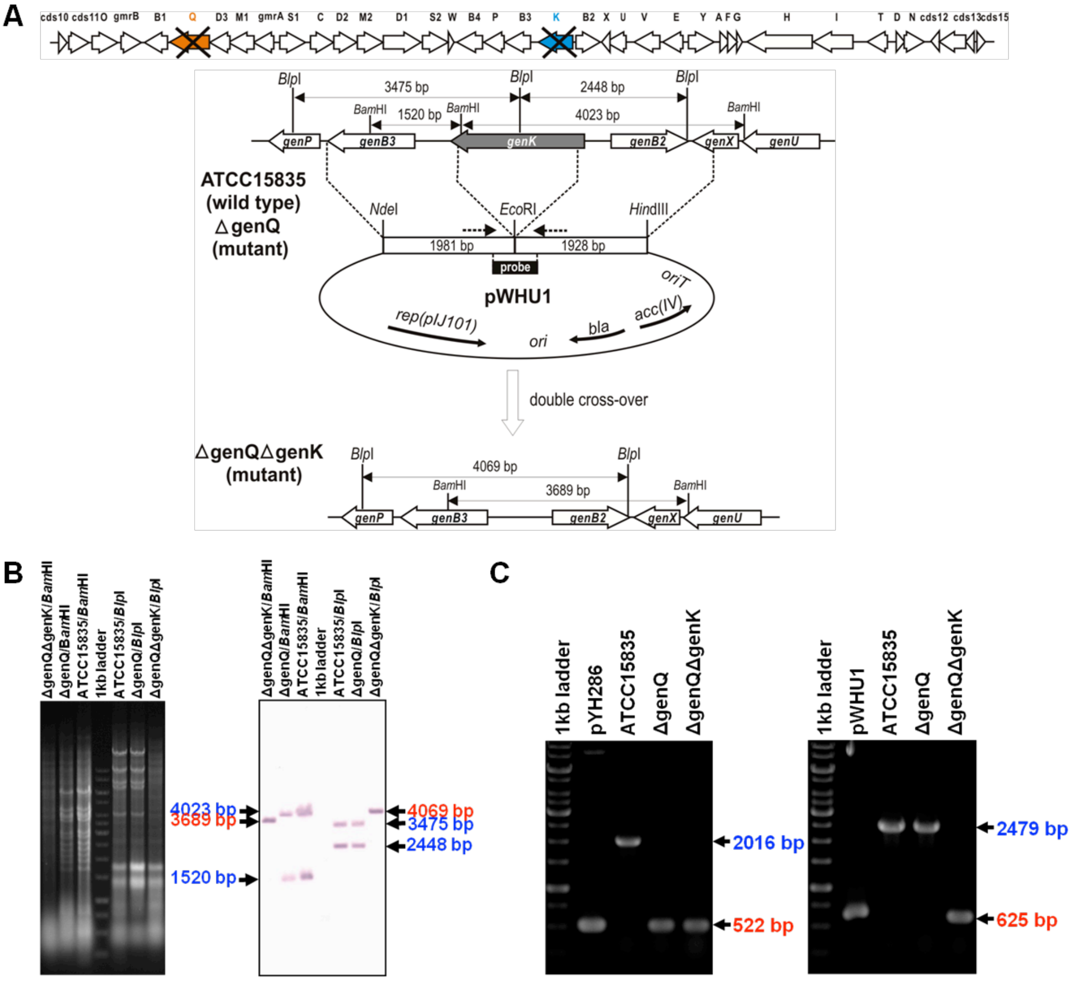

(4) Complementation of ΔgenK and ΔgenQ

Confirmation of complementation strains (A) ΔgenK::*genK*, (B) ΔgenQ::*genQ* and (C) ΔgenQ::*neo11* by PCR. The arrows indicate the expected size of the PCR fragments in the wild type and mutants. (D) Map of genetic complementation vector. The dash arrows indicate the location of PCR primers for check complementation strains.

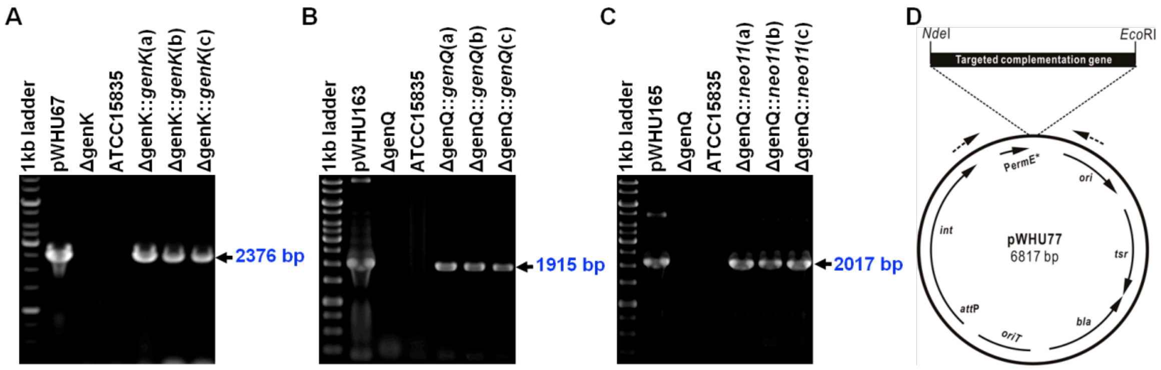

Figure S3, related to Figure 2. LC-HRMS analysis of production of gentamicin C complex and intermediates by the wild type, ΔgenK, ΔgenQ, and ΔgenQΔgenK mutants of *M. echinospora* ATCC15835

(A) Schematic representation of the in-frame deletion of *genQ* and *genK*. Deleted genes are shown in dotted arrows. (B) Production of gentamicin C complex and intermediates analyzed by LC-ESI-HRMS and bioassay using *Bacillus pumilus* as indicator strain. -: no production, +: production, ++: increased by about 10-fold compared with wild type, +++: increased by about 100-fold compared with wild type.

A

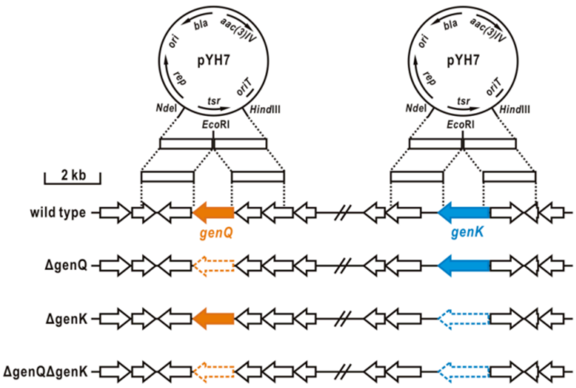

B

	Production									Bioassy
	C1a	C2	C2a	C2b	C1	X2	JI-20A	G418	JI-20B	
wild type	+	+	+	+	+	+	+	+	+	
ΔgenQ	−	−	−	−	−	+	−	+++	−	
ΔgenK	++	−	−	++	−	++	++	−	−	
ΔgenQΔgenK	−	−	−	−	−	+++	−	−	−	

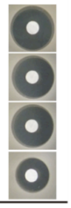

Figure S4, related to Figure 3. (A) Sequence alignment of GenB1, GenB2, GenB3 and GenB4 with their homolog Neo18 from the neomycin biosynthetic pathway. The red box in (A) indicates the putative catalytic lysine; (B) Schematic representation and confirmation by Southern blot of in-frame deletion of *genB1, genB2, genB3 and genB4.* (1) ~ (4): Single mutants; (5) ~ (10): Double mutants; (11) ~ (14): Triple mutants; (15): Quadruple mutant.

A

```
Neo18           ----------------------------------------------MTKNS---SLLAEF 11
GenB1           ----------------------------------------------MTIDIGAGKLLAQE 14
GenB2           ------------------------------------------------------MIIANA 6
GenB3           MAVADHRSSEPSWRAGRTARRRSRWHSWARVKSAREGSQDMDSANLTNRGLVERARRVTA 60
GenB4           ---------------------------------------------MNYRELIERARRTTA 15
                                                                         .

Neo18           PTCPRDEKDRPRVFTAASGAWLTDESGFRWIDFDNARGSILLGHGDPVVAEAVARAAT-- 69
GenB1           PTCPRDADGRPRVFVEGSGAYLTDPDGRRWIDFDNARGSVVLGHGDEEVAEAIARAAR-- 72
GenB2           DGCTPYEVARGVTIVRGEGAYVYDAEGRGLIDLSNSFGSVMLGHQDPVVTEAVLKTVR-- 64
GenB3           AENYDIGTRFSAMIQSGEGAWLTDVEGNRYVDLTASSGTIILGHRNQAVTEAITRQIRDF 120
GenB4           AEEYDISGRYPSVIAHAEGAWMTDLSGNRYVDLTGADAAVILGYRHPAVNEAITRQIRDY 75
                             :  ..**:: * .*   :*:  : .:::**: .  * **: :

Neo18           GADGTATGWSRRVDAVLERLHALCGGEVVGLFRSGTAAVRAAVLAVREATGRPLLLSAGY 129
GenB1           GRSGVGTAWSPVLDSLLGQLQEVCGGDVVGLYRTGTAALRSVTCAVRDARDRSIVLSSGY 132
GenB2           SGVPAAASLDLQNHLAEQIAGDLPGDQRVAFFKTGTAATRAAASAARQVTGKRLIASCGY 124
GenB3           GTAFASTLSVPRVELAERLCERYECAEKVVFHKTGSEGTAMAARLARAATGRELILSCGY 180
GenB4           GTTFASTLSVPRVELAERMCERYECAEKVVFHKTGTEGTAMAVRLARAATGRELVLSSGY 135
                .   ..:      .            : * :.::*: .   ..  .* . .: :: *.**

Neo18           HGYDPMWYPSEAPLEPNADGVVDFFFDLGLLRELLRA-PERVAAVVVSPDHMHLSPGWYR 188
GenB1           HGYDPMWH-CDEPFTPNQHGIVEFLFDLDVLAEWLSR-PEQVAAVVISPDHMHLGERWYT 190
GenB2           HGYDLMWE-FTPPGQPNSEDVLHCYHLPELIDQVLDKHAHELAAVIIAPDYIHVSPEYIA 183
GenB3           HGWHEWQLAGETFGYQQTTGVVGFGYNEKALAKMLEAFGNEVAGVLISPELLYFDVEFYQ 240
GenB4           HGWHEWQMAGEEFGYQQSTGVVGFGYNEKALAKMLEAFGEQVAGVIVSPEVLYFDLDHYR 195
                **:.            :  .::   .    : : *    ..:*.*:::*: ::..

Neo18           ELRRLCSAAGVVLVADEVKVGLRYAPGLSTAE-LLAPDVWVVAKGMANGHAVSAVGGSRR 247
GenB1           EFTRLTKEADVPVIADEVKVGLRYRAGLSTP--LLDPAVWIVAKCLANGSPVAAVGGDAH 248
GenB2           DLFERCERVGVVTIADEVKHGYRLRQGASVTEASVVADMYTYAKGISNGWPLSCVAGDER 243
GenB3           RMYALCARYDVPFMMDEVYTGFRAGP-KGVHGLGVPADVVVVSKGLANGHSLAAVMGRRD 299
GenB4           RMSALCARYDVPFMLDEVYTGFRAGP-KGVHGLGVPADVVVLGKGLANGHSLAAVMGRRD 254
                 :       .*  : ***  * *     ..    : . :   .* ::** .::.* *

Neo18           LLKP--LKEVSFTSFFEPTILAAADAALARVATGEPQRAVREAGDRFLRHARKALDDASL 305
GenB1           LLAA--LEDVSFTSYFEPTAMAAATTTLRRMATGEPQQAIRAAGDRFIAHTRAAFANAGV 306
GenB2           FLKP--LAEFVSTLTFEAPSFAAASATLDRLAELDVQAQLAIDGARFVSEAAKMISTRDL 301
GenB3           IIDAYDVSGIQGTYTREVPPMAAAMAVLDVLDTPGVYEHAEAMGRRLADGMREILTGEGI 359
GenB4           IIDAYDVSGIQGTYTREVPPMAAALAVFEVLDTPGVYEHAEAMGRRLADGMREILTGEGI 314
                :: .  :  .  *   * . :*** :.:  :            * *:       :   .:

Neo18           PVEIAGDGTFFQFVPATEELEEALYGAANAEGLLFYAGDNQGVSAAFDEAVLGEAERRFA 365
GenB1           PIDLAGNGNLFQFVCADDEVADAFHAAAAAEGLLFFEGDNQTPSAAFTDEVVEDACGRID 366
GenB2           PIEMAGTGAAFQFVCA-EEVEEVLLPHALAEGLILEPSDQQYPSACFRGEVVDDALERLD 360
GenB3           PNWVGGPALMFDTVLPNDDLGWEIYKTAHDFGVYFEDSGTQLVTTAFDEAAVDHALTAFR 419
GenB4           PNWVGGPALMFDVVLPNDDLGWEIYKTAHDFGVYFEDSGTQLVTAAFDEAAVDHALTAFR 374
                *  :.* .  *: * . :::   :   *   *: :  .. *  ::.*   .: .*   :

Neo18           RVCERLAPY---AGGEPVGDAARYRVAWNVMDGLRQAPRDREETTGLLARLLDD------ 416
GenB1           RVSAALTGR---FTDRELTEESWYASAWGAMDGLADRPRTREETTAIVERLWED------ 417
GenB2           RALTTMAAARPDLVGREVTQLDRVNAAFCQMDGLPGRPDGWSLDQCVEYVTAQL------ 414
GenB3           KATRQVIADRPDIAPTSGGELTEERKLDFAEEAFGGLLRDDERTNALIDETIEKVVNRDR 479
GenB4           KATRQVVADRPDIAPTSGGELTEERKLDFAEEAFGGLLRDDERTNALIDETIEKVVNRDR 434
                :.   :             :           :.:       .    :     :

Neo18           -----------
GenB1           -----------
GenB2           -----------
GenB3           SIKPVLIPAQN 490
GenB4           SIKPVLFPAQN 445
```

B

(1) ΔgenB1

The arrows indicate the expected size of the fragments from the wild type and mutant chromosomal DNA, respectively, hybridized after digestion with *Age*I with a 581 bp probe (PCR-amplified from pWHU4 using oligonucleotide primers genB1-CK1 and CK2).

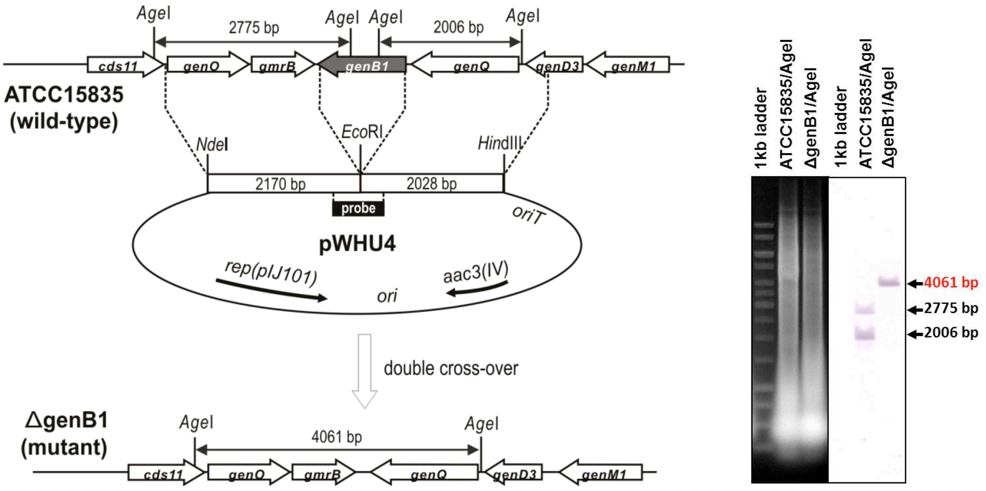

(2) ΔgenB2

The arrows indicate the expected size of the fragments from the wild type and mutant chromosomal DNA, respectively, hybridized after digestion with *Nsp*I with a 587 bp probe (PCR-amplified from pWHU2 using oligonucleotide primers genB2-CK1 and CK2).

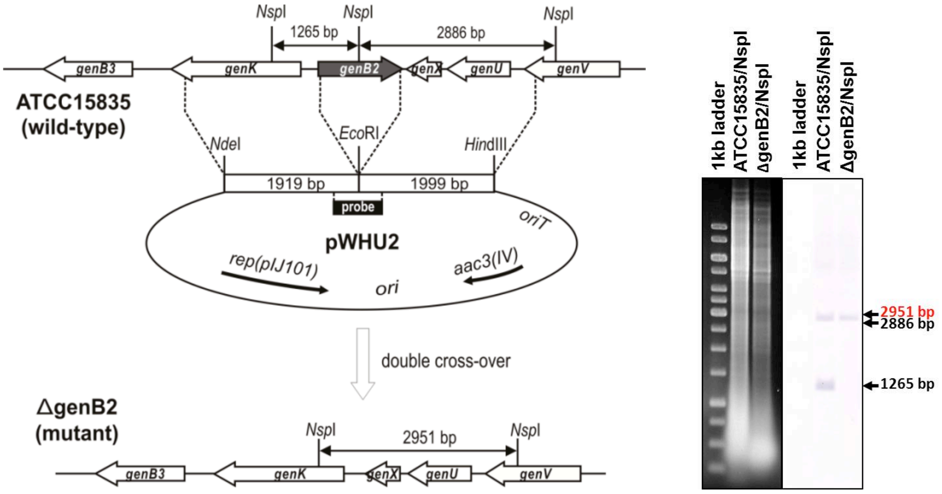

(3) ΔgenB3

The arrows indicate the expected size of the fragments from the wild type and mutant chromosomal DNA, respectively, hybridized after digestion with *Nsp*I with a 658 bp probe (PCR-amplified from pWHU5 using oligonucleotide primers genB3-CK1 and CK2).

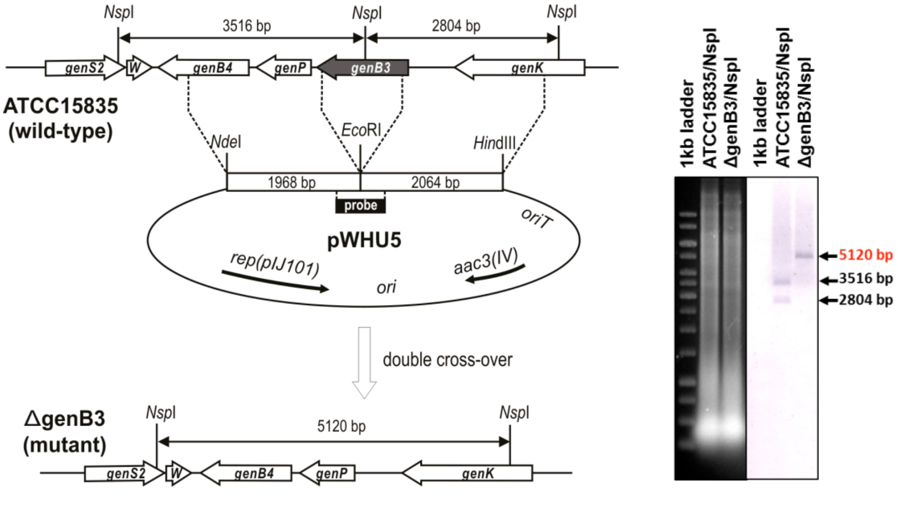

(4) ΔgenB4

The arrows indicate the expected size of the fragments from the wild type and mutant chromosomal DNA, respectively, hybridized after digestion with *Sac*II with a 595 bp probe (PCR-amplified from pWHU3 using oligonucleotide primers genB4-CK1 and CK2).

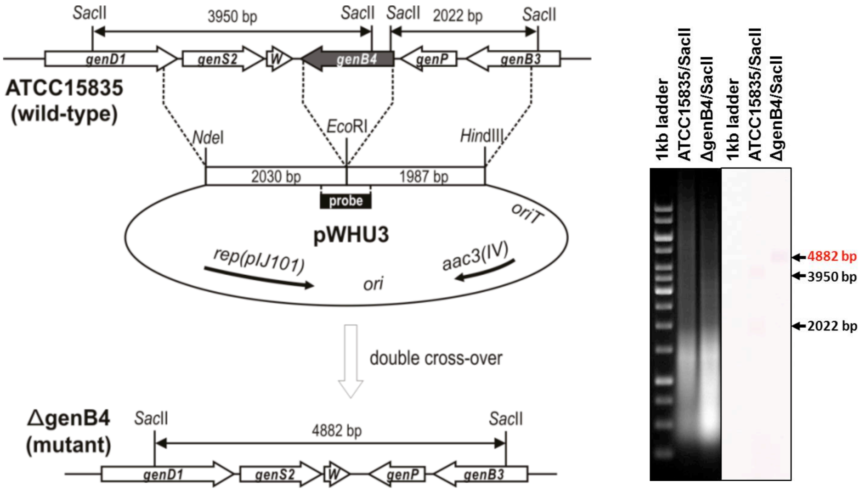

(5) ΔgenB2ΔgenB1
The arrows indicate the expected size of the fragments from the wild type and mutants chromosomal DNA, respectively, hybridized after digestion with *Age*I with a 581 bp probe (PCR-amplified from pWHU4 using oligonucleotide primers genB1-CK1 and CK2).

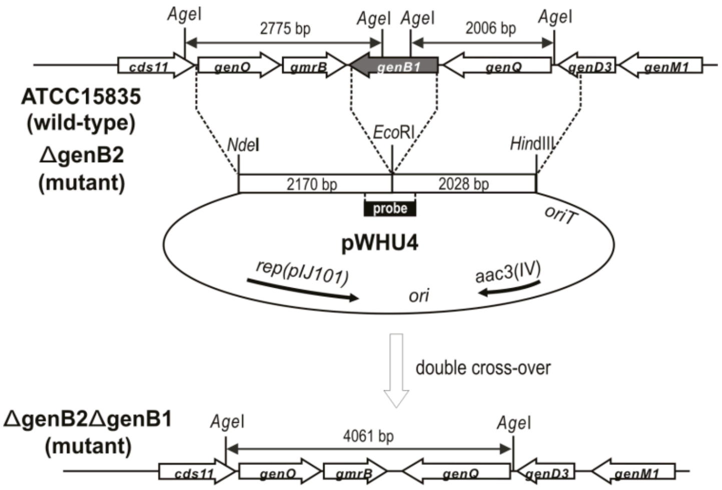

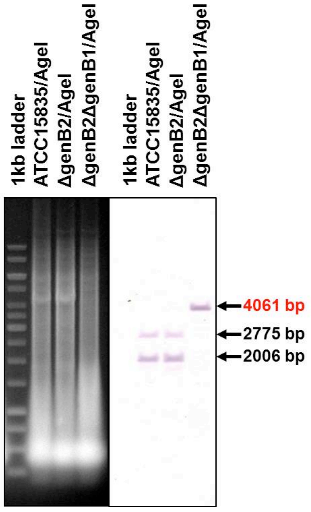

(6) ΔgenB1ΔgenB3
The arrows indicate the expected size of the fragments from the wild type and mutants chromosomal DNA, respectively, hybridized after digestion with *Psh*AI to a 658 bp probe (PCR-amplified from pWHU5 using oligonucleotide primers genB3-CK1 and CK2).

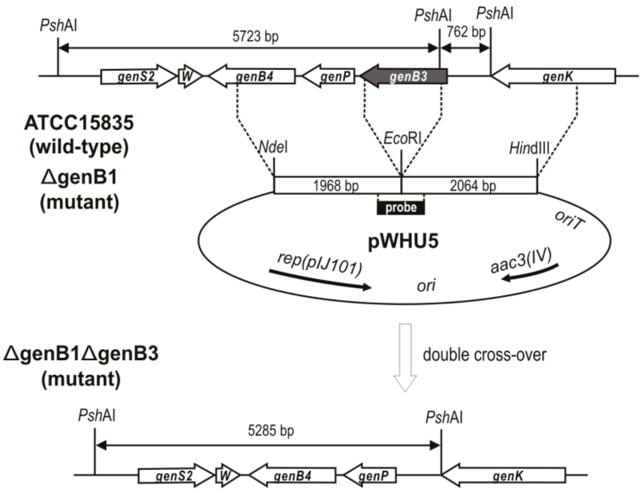

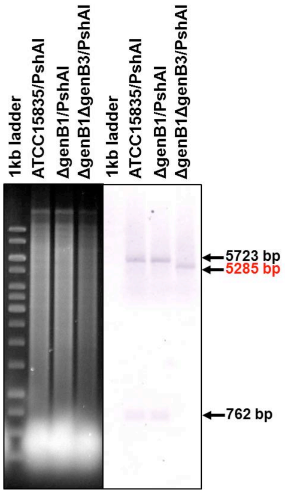

(7) ΔgenB4ΔgenB3

The arrows indicate the expected size of the fragments from the wild type and mutants chromosomal DNA, respectively, hybridized after digestion with *Sac*II with a 741 bp probe (PCR-amplified from pWHU43 using oligonucleotide primers genB4-genB3-CK1 and CK2).

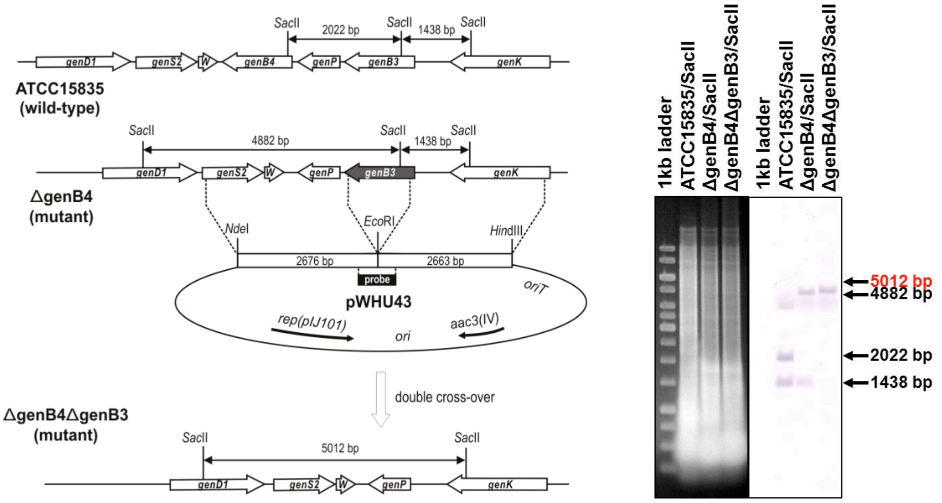

(8) ΔgenB2ΔgenB3

The arrows indicate the expected size of the fragments from the wild type and mutants chromosomal DNA, respectively, hybridized after digestion with *Psh*AI with a 658 bp probe (PCR-amplified from pWHU5 using oligonucleotide primers genB3-CK1 and CK2).

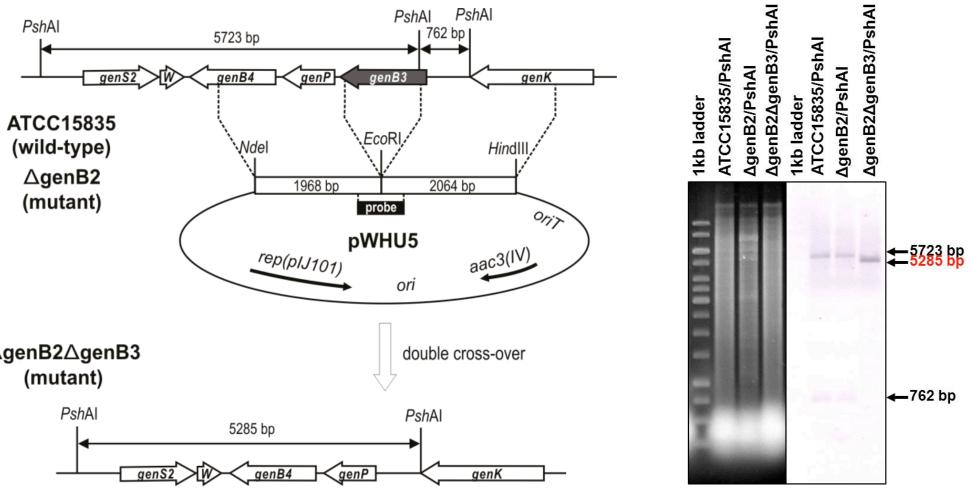

(9) ΔgenB2ΔgenB4

The arrows indicate the expected size of the fragments from the wild type and mutants chromosomal DNA, respectively, hybridized after digestion with *Sac*II with a 595 bp probe (PCR-amplified from pWHU3 using oligonucleotide primers genB4-CK1 and CK2).

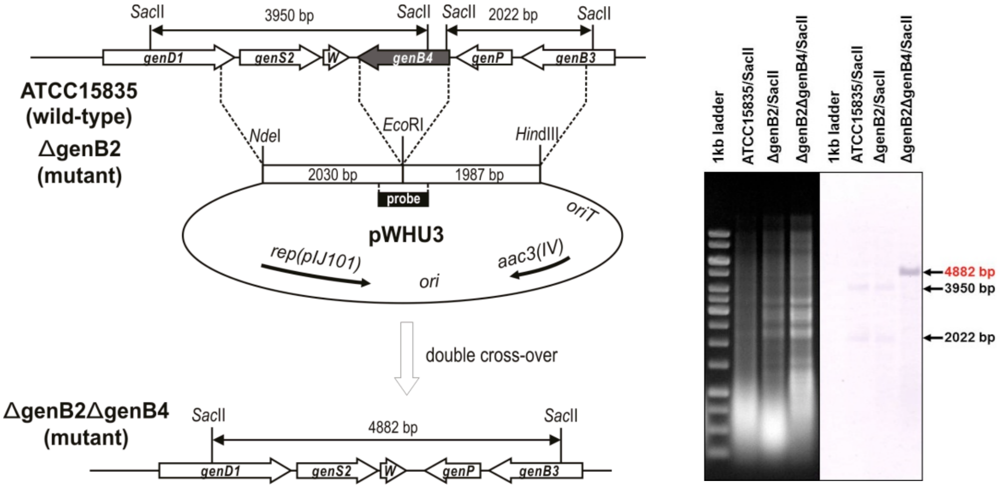

(10) ΔgenB4ΔgenB1

The arrows indicate the expected size of the fragments from the wild type and mutants chromosomal DNA, respectively, hybridized after digestion with *Age*I with a 581 bp probe (PCR-amplified from pWHU4 using oligonucleotide primers genB1-CK1 and CK2) respectively,.

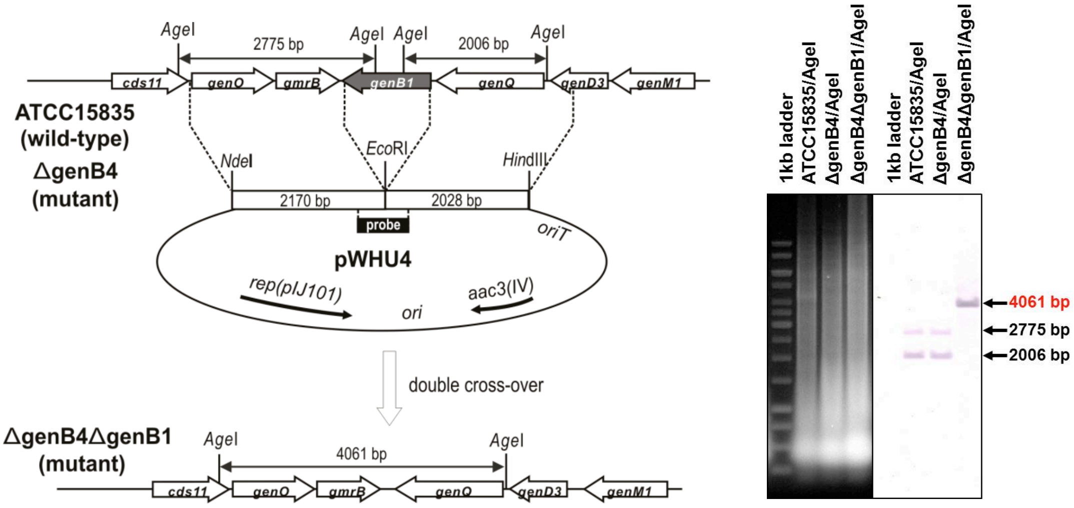

(11) ΔgenB4ΔgenB1ΔgenB2

The arrows indicate the expected size of the fragments from the wild type and mutant schromosomal DNA, respectively, hybridized after digestion with *Pvu*I with a 587 bp probe (PCR-amplified from pWHU2 using oligonucleotide primers genB2-CK1 and CK2).

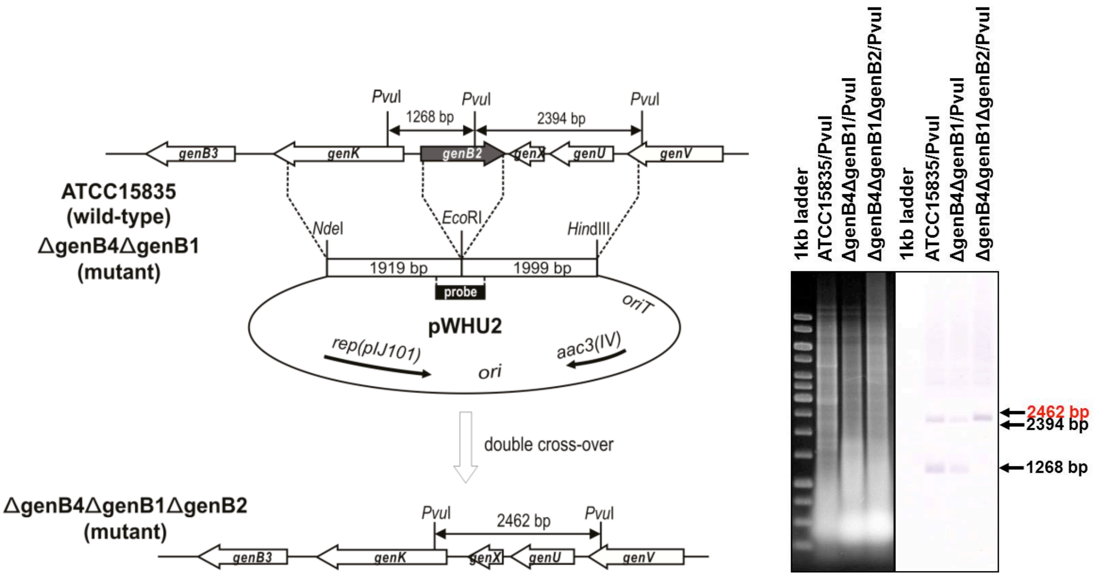

(12) ΔgenB4ΔgenB1ΔgenB3

The arrows indicate the expected size of the fragments from the wild type and mutants chromosomal DNA, respectively, hybridized after digestion with *Sac*II with a 741 bp probe (PCR-amplified from pWHU43 using oligonucleotide primers genB4-genB3-CK1 and CK2).

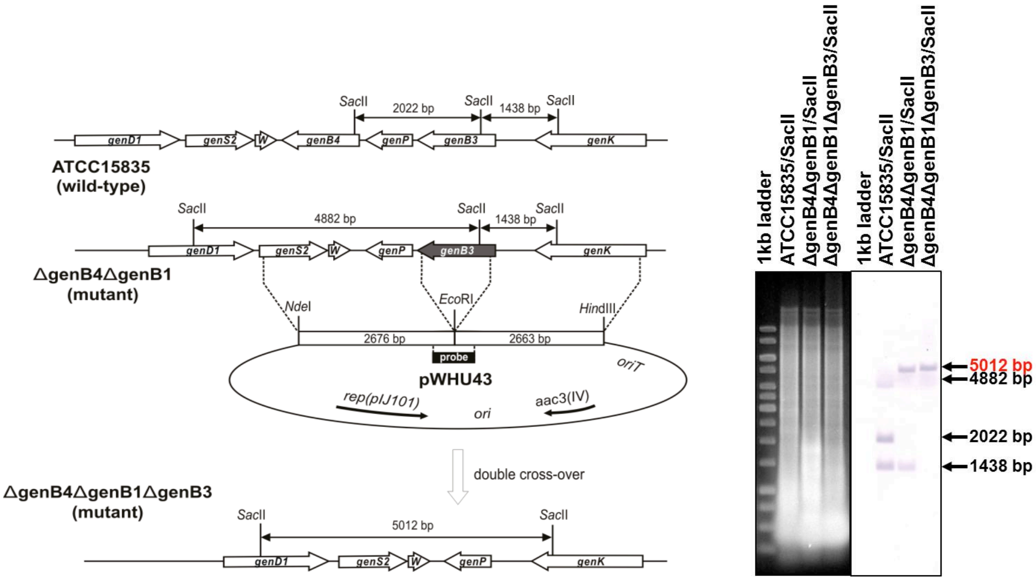

(13) ΔgenB2ΔgenB3ΔgenB1

The arrows indicate the expected size of the fragments from the wild type and mutants chromosomal DNA, respectively, hybridized after digestion with *Age*I with a 581 bp probe (PCR-amplified from pWHU4 using oligonucleotide primers genB1-CK1 and CK2).

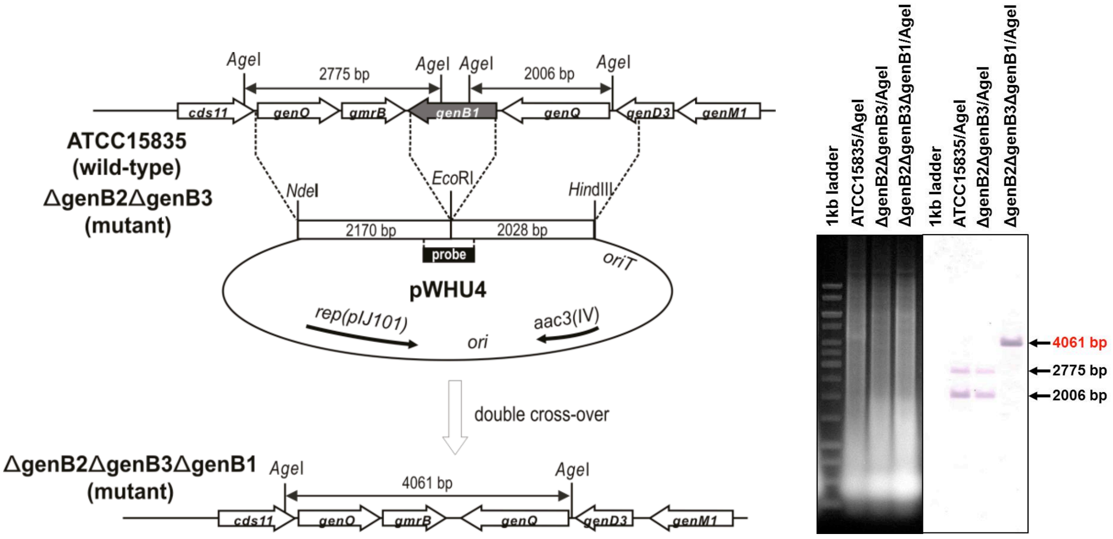

(14) ΔgenB2ΔgenB4ΔgenB3

The arrows indicate the expected size of the fragments from the wild type and mutants chromosomal DNA, respectively, hybridized after digestion with *Sac*II with a 741 bp probe (PCR-amplified from pWHU43 using oligonucleotide primers genB4-genB3-CK1 and CK2).

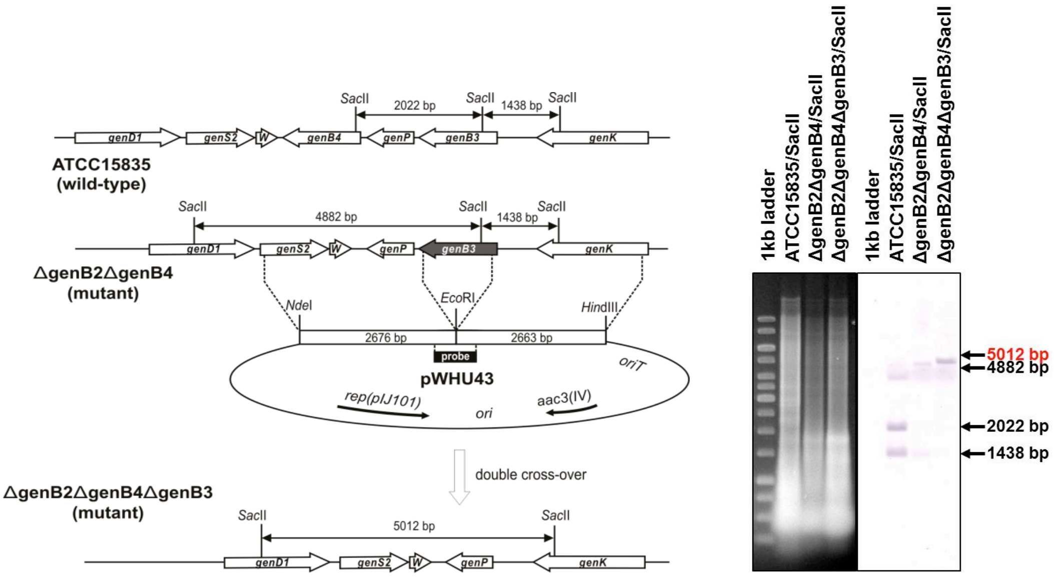

(15) ΔgenB4ΔgenB1ΔgenB3ΔgenB2

The arrows indicate the expected size of the fragments from the wild type and mutants chromosomal DNA, respectively, hybridized after digestion with *Pvu*I with a 587 bp probe (PCR-amplified from pWHU2 using oligonucleotide primers genB2-CK1 and CK2).

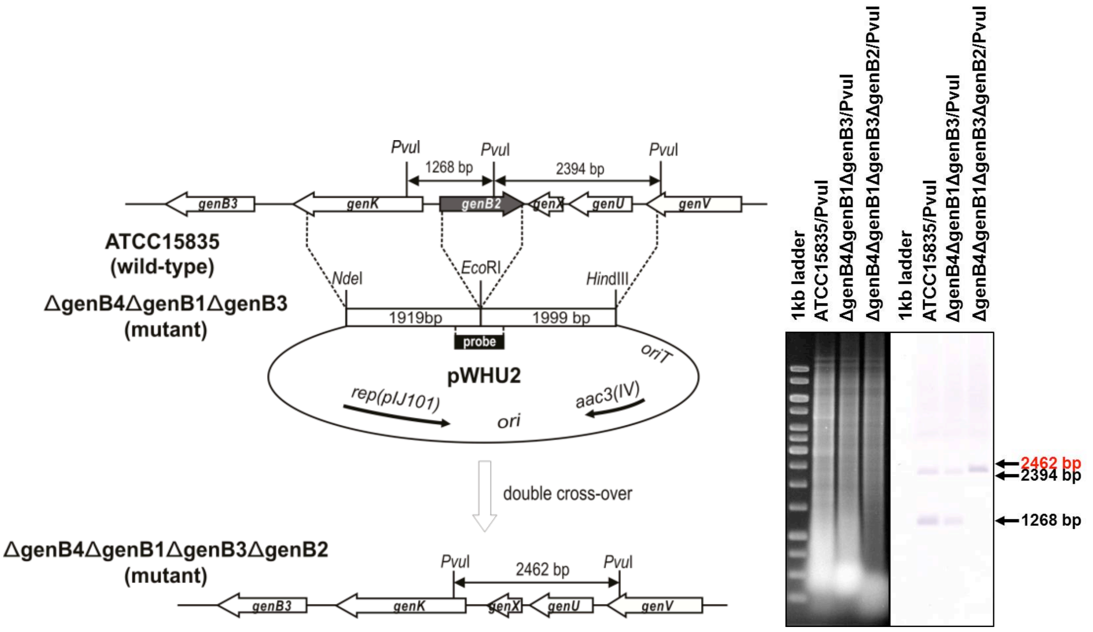

Figure S5, related to Figure 3. Confirmation of C2a production in ΔgenB2 and ΔgenB2ΔgenB1 mutants

(A) Comparison of product mixture from strain ΔgenB2 with gentamicin C2 standard; (B) Comparison of product mixture from strain ΔgenB2ΔgenB1 with gentamicin C2 standard; (C) Comparison of product mixture from strain ΔgenB2 with gentamicin C2a standard; (B) Comparison of product mixture from strain ΔgenB2ΔgenB1 with gentamicin C2a standard; (E) Comparison of product mixture from strain ΔgenB2 with gentamicin C1 standard; (F) Comparison of product mixture from strain ΔgenB2ΔgenB1 with gentamicin C1 standard.

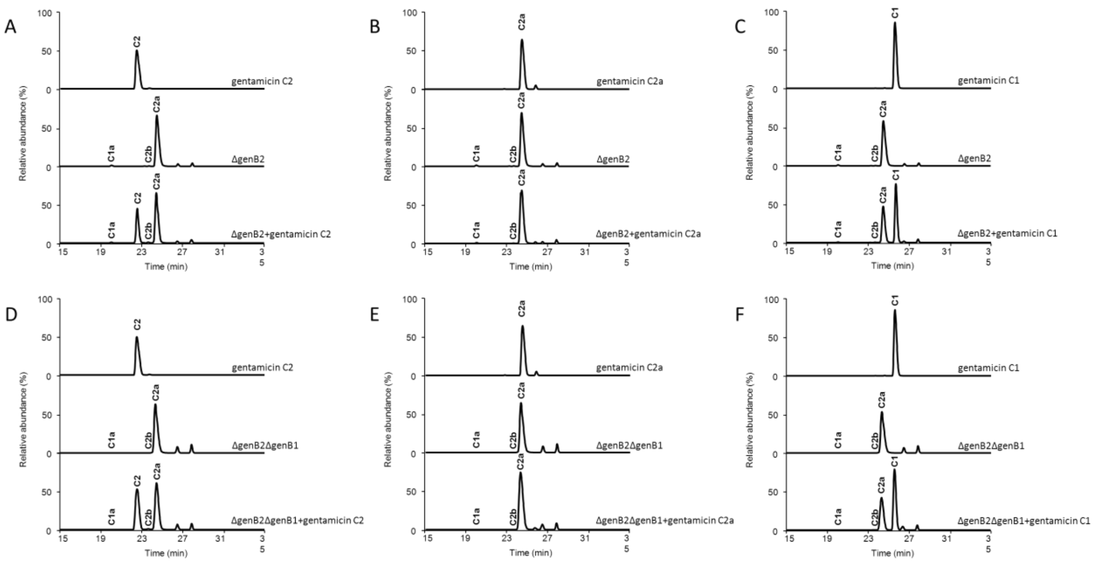

Figure S6, related to Figures 4, 5 and 6. Cloning and characterization of GenQ, GenB1, GenB2, GenB3 and GenB4

(A) Cloning *genQ* with alternative start codons. Positions of the original start codon of *genQ* and the new start codons in LM_*genQ* and VM_*genQ* are indicated in red boxes; (B) SDS-PAGE gel of VM_GenQ, GenB1, GenB2, genB3 and GenB4; (C) UV-Vis spectra of VM_GenQ and FAD standard; (D) UV-Vis spectra of GenB proteins.

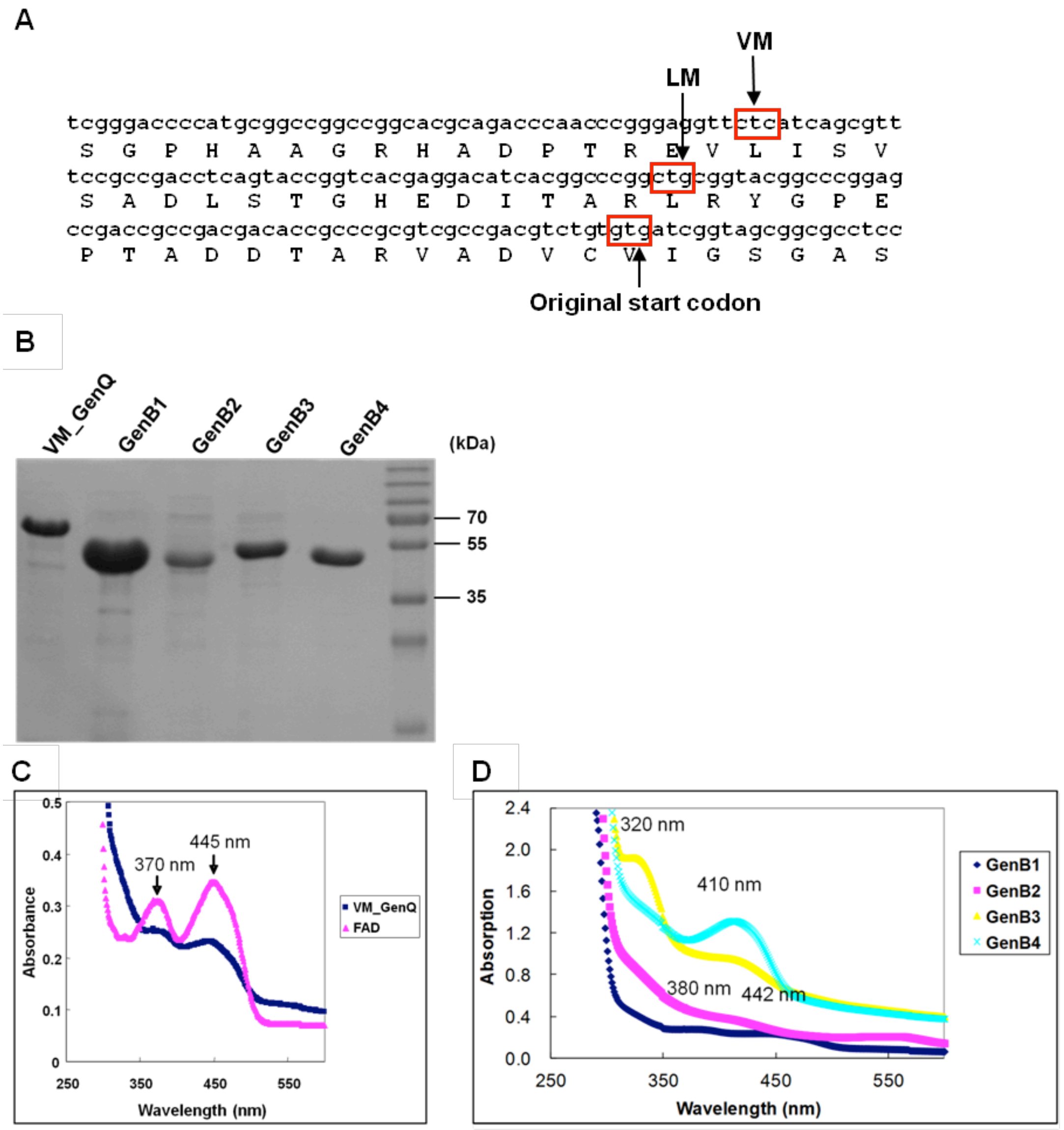

Figure S7, related to Figures 4 and 5. LC-MS analysis of activity assays of GenB1, GenB2, GenB3 and GenB4 (GenB)

(A) Test of different amino donors on the conversion of G418 to JI-20B (i - iv) and of gentamicin X2 to JI-20A(v - viii) catalysed by VM_GenQ coupled with GenB1, GenB2, GenB3, and GenB4 by LC-MS analysis. The percentages of G418, gentamicin X2, 6'-DOG, 6'-DOX, JI-20B and JI-20A present after 16 h of incubation, based on the abundance of corresponding ions detected, are shown as stacked plots. (B) LC-MS analysis of isomerase activity of GenB enzymes on gentamicin C2 and C2a. Selective ion monitoring $[M+H]^+$ (*m*/*z* 464) and $[M+Na]^+$ (*m*/*z* 486) ions of C2 and C2a.

A

i

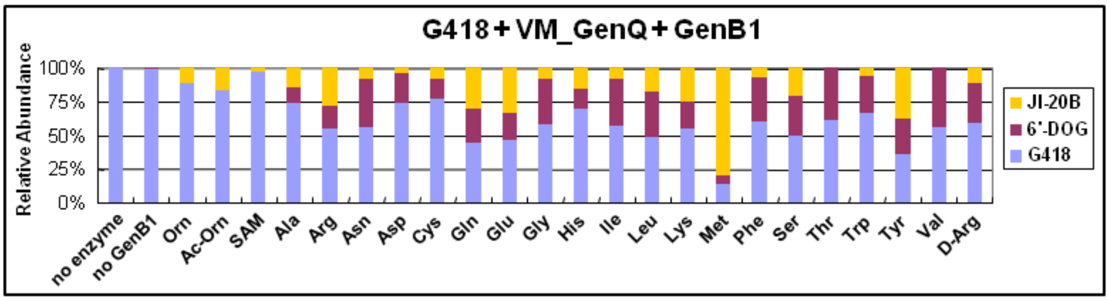

ii

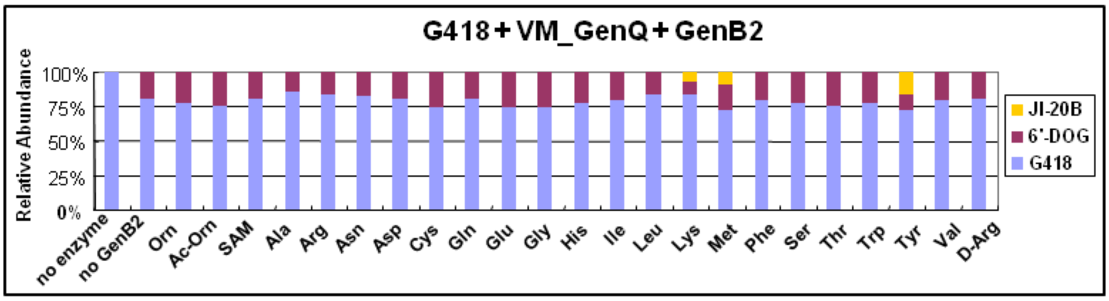

iii

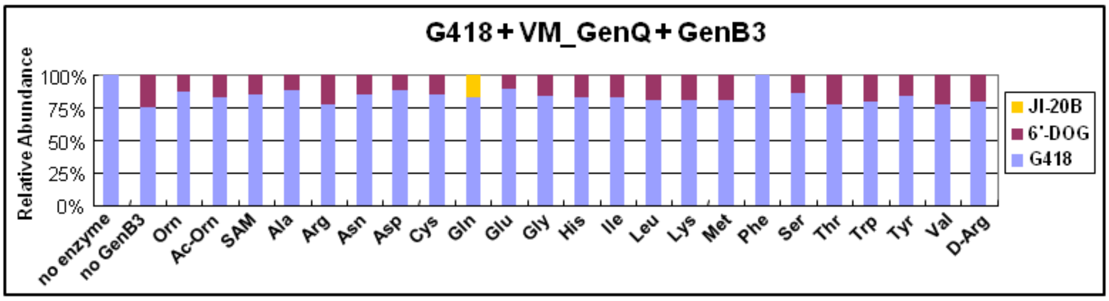

iv

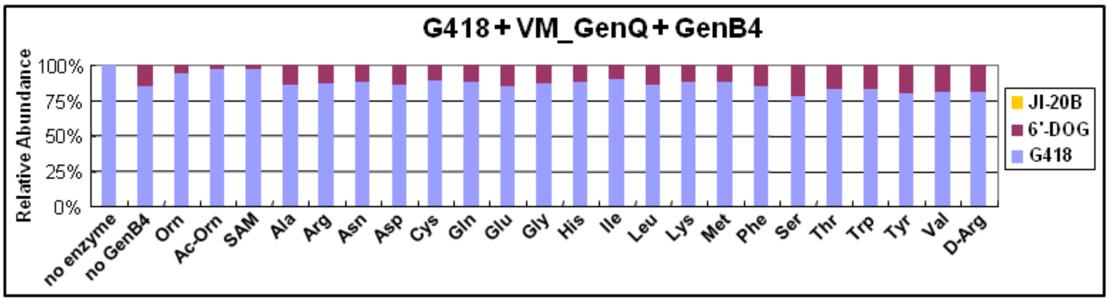

v

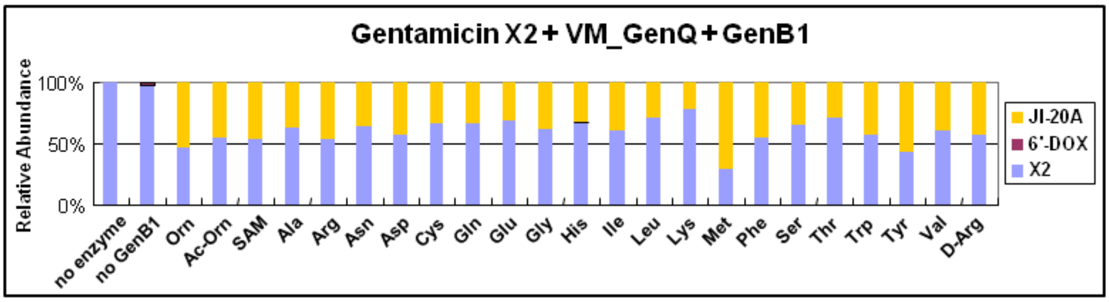

vi

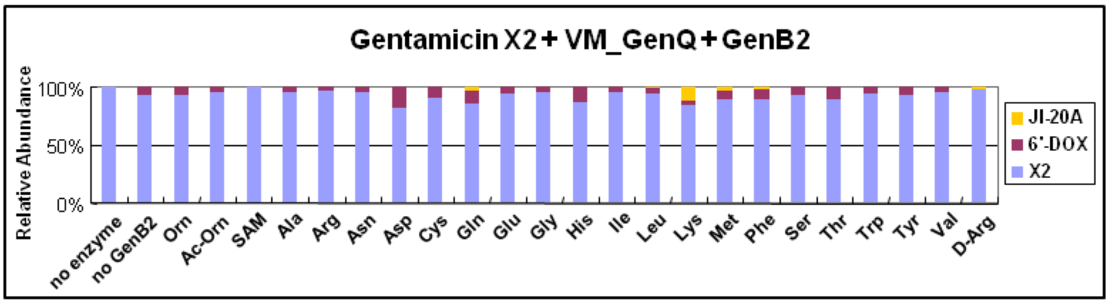

vii

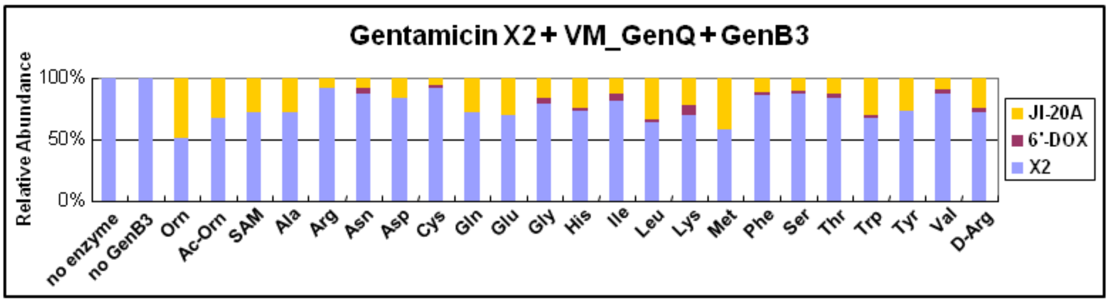

viii

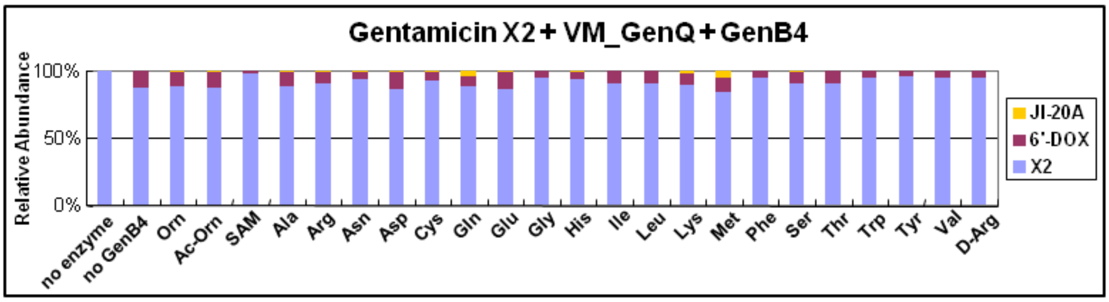

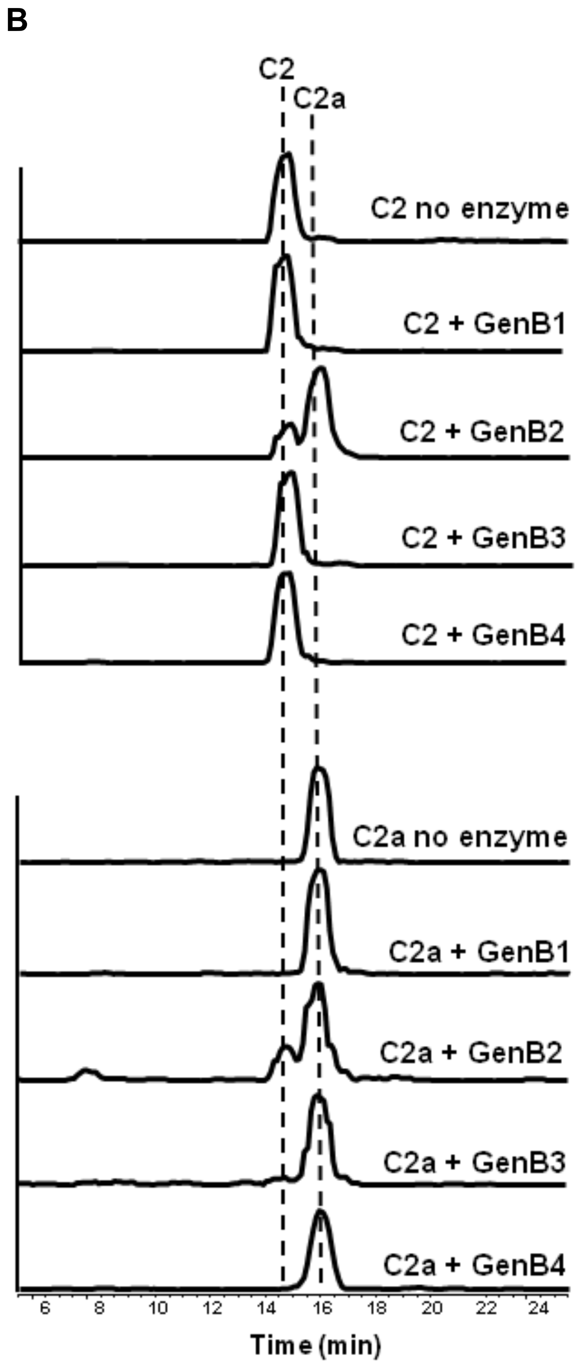
B
C2
C2a
C2 no enzyme
C2 + GenB1
C2 + GenB2
C2 + GenB3
C2 + GenB4
C2a no enzyme
C2a + GenB1
C2a + GenB2
C2a + GenB3
C2a + GenB4
6
8
10
12
14
16
18
20
22
24
Time (min)

List of oligonucleotide primers used in this study, related to Experimental Procedures

**Primer**	**Oligonucleotide sequences (5' to 3')**	**Restriction site**
genQ-L1	CTG**CATATG**CTCACCGCCCGGC	*Nde*I
genQ-L2	CCC**GAATTC**TCCCTGTGACCTGTCG	*Eco*RI
genQ-R1	ACC**GAATTC**GCCGCTACCGATCACAC	*Eco*RI
genQ-R2	GGC**AAGCTT**GATCGGAACCATCCGG	*Hin*dIII
genQ-CP1	CCTCCTCGTCACCGTGG	-
genQ-CP2	CAGGTGCTCAGCGTCCG	-
genQ-EP1	GTC**CATATG**ATCGGTAGCGGCGCC	*Nde*I
genQ-EP2	CGA**GAATTC**ACAGGGAGAAATCGG	*Eco*RI
neo11-EP1	CGC**CATATG**AAGCGCCTTCGAGGC	*Nde*I
neo11-EP2	CCG**GAATTC**TCAGACGTGCGCGGTGTG	*Eco*RI
genK-L1	GCG**CATATG**AGAACCGGCTTGATGC	*Nde*I
genK-L2	GCG**GAATTC**CACTGACCCTCGTCCC	*Eco*RI
genK-R1	GAG**GAATTC**CTTCACTTGATTACC	*Eco*RI
genK-R2	CAC**AAGCTT**CACCGGCGAGACG	*Hin*dIII
genK-CP1	CGGGCGAACCTTCGGGATA	-
genK-CP2	CCGTCAGCGTTGGCAATAA	-
genK-EP1	CGC**CATATG**AACGCGCTGGTGGCA	*Nde*I
genK-EP2	CCG**GAATTC**TCAGTGGGAAACCGCCTC	*Eco*RI
genB1-L1	GCA**CATATG**CTGGAGAATCCGTCTG	*Nde*I
genB1-L2	GTC**GAATTC**CTCTGGGAGGACTGAG	*EcoR*I
genB1-R1	ACC**GAATTC**GATGGTCATCGTGTCG	*EcoR*I
genB1-R2	GAA**AAGCTT**GACCGTCAACCTGGCG	*Hin*dIII
genB1-CK1	GAGTCGTCGTACTTCTGGATGGG	-
genB1-CK2	GCTGACCTGGGGTTGCTGC	-
genB2-L1	GCG**CATATG**TCACCGGCGAAGCGGC	*Nde*I
genB2-L2	CGT**GAATTC**GTCAGCGTTGGCAATA	*EcoR*I
genB2-R1	GTG**GAATTC**GTGACCGCTCAGCTCT	*EcoR*I
genB2-R2	GTC**AAGCTT**GTTGCAGGCGGTCCAG	*Hin*dIII
genB2-CK1	GCCGACTTCGACCTCTTCC	-
genB2-CK2	ATCCGCTGACCCCGTGCC	-

genB3-L1	CGT**CATATG**GCAACACCACGTCG	*Nde*I
genB3-L2	GAC**GAATTC**ATCGAGAAGGTGGTC	*EcoR*I
genB3-R1	CGA**GAATTC**GGTCCCGATGTCGTAG	*EcoR*I
genB3-R2	CTT**AAGCTT**TGGCGAGGGGCTCTGC	*Hin*dIII
genB3-CK1	CGCGTTACGGAAAGTAAAATCAC	-
genB3-CK2	CATCGAGGGCCACCACC	-
genB4-L1	CGC**CATATG**CAGCGGTTCAAGGGCG	*Nde*I
genB4-L2	GTC**GAATTC**CCGGCACAGAACTGAC	*EcoR*I
genB4-R1	GAT**GAATTC**ACGGTAGTTCATGTGC	*EcoR*I
genB4-R2	CGG**AAGCTT**TGTGTGAGCGGTACGG	*Hin*dIII
genB4-CK1	TGACTTCTGCCTCGACAACG	-
genB4-CK2	AAGCTCTACCTGGAGACCTTCC	-
genB4-genB3-L1	GGC**CATATG**CGGTGGCGACCC	*Nde*I
genB4-genB3-L2	CGC**GGTACC**AAGCCGGTTCTCA	*Kpn*I
genB4-genB3-R1	CAA**GGTACC**AGAATCCATGTCC	*Kpn*I
genB4-genB3-R2	AAT**AAGCTT**GGGAATTAGTCCAG	*Hin*dIII
genB4-genB3-CK1	CAGCGTTCCTCCGGTTTC	-
genB4-genB3-CK2	GGTAAGTGATCCGCAGTTGG	-
pGenQ-LM_up	ATCACGGCC**CATATG**CGGTACGGCCCGGAG	*Nde*I
pGenQ-VM_up	CCCAACCCGG**CATATG**CTCATCAGCGTTTC	*Nde*I
pGenQ-dn	TTGCCGGCACC**GAATTC**GATGGTCATCGTG	*Eco*RI
pGenB1-up	AATCGAGCGAC**CATATG**ACCATCGACATCG	*Nde*I
pGenB1-dn	AGGACCTGGTGG**GGATCC**ACAAGGAGTGAA	*Bam*HI
pGenB2-up	CGGAGGTTTCG**CATATG**ATTATTGCCAACG	*Nde*I
pGenB2-dn	CGGCCCCTGCC**GGATCC**GTCAGAGCTGAGC	*Bam*HI
pGenB3-up	TTG GGGATAG**CATATG**GCAGTCGCCGACCA	*Nde*I
pGenB3-dn	ATCTTCACCGGCCCTG**GAATTC**CTACGCC A	*Eco*RI
pGenB4-up	CGTGGTGGG**CATATG**AACTACCGTGAGTTG	*Nde*I
pGenB4-dn	CGGCCCTCGAACCG**GAATTC**CTCAAGGTCA	*Eco*RI

Primer pairs for amplification of left- or right-flanking fragments of a target gene, for PCR/sequencing confirmation, and for cloning target genes for over-expression are marked with suffixes –L1/–L2, -R1/-R2, CK1/CK2, or –up/-dn, respectively.

Table S1, related to FIgure 3. LC-HRMS analysis of *genB* knock-out mutants

Strain	Gentamicin C complex production (×10^5)					Intermediates production (×10^5)			
	**C1a**	**C2b**	**C2**	**C2a**	**C1**	**X2**	**JI-20A**	**G418**	**JI-20B**
wild type	8.8	2.8	11.9	1.1	44.6	8.5	1.5	8.2	4.3
ΔgenB1	0.4	2.9	1.2	1.7	14.6	6.8	0.5	15.9	2.5
ΔgenB2	0.8	0.1	ND	7.6	ND	7.1	0.7	0.7	1.0
ΔgenB3	ND	ND	ND	ND	ND	7.2	19.1	10.8	75.4
ΔgenB4	ND	ND	ND	ND	ND	11.0	2.7	14.1	8.7
ΔgenB2ΔgenB3	ND	ND	ND	ND	ND	8.6	2.7	8.8	20.5
ΔgenB2ΔgenB4	ND	ND	ND	ND	ND	7.6	2.0	1.2	3.7
ΔgenB4ΔgenB1	ND	ND	ND	ND	ND	5.2	1.4	18.6	6.7
ΔgenB2ΔgenB1	0.3	0.1	ND	15.3	ND	4.0	0.5	10.7	2.9
ΔgenB1ΔgenB3	ND	ND	ND	ND	ND	1.5	0.1	43.0	1.4
ΔgenB4ΔgenB3	ND	ND	ND	ND	ND	9.7	1.1	17.0	6.3
ΔgenB2ΔgenB3ΔgenB1	ND	ND	ND	ND	ND	3.4	0.1	12.8	0.1
ΔgenB4ΔgenB1ΔgenB2	ND	ND	ND	ND	ND	1.9	0.1	40.3	3.4
ΔgenB4ΔgenB1ΔgenB3	ND	ND	ND	ND	ND	3.3	0.1	20.8	0.8
ΔgenB2ΔgenB4ΔgenB3	ND	ND	ND	ND	ND	7.4	2.5	20.8	35.2
ΔgenB4ΔgenB1ΔgenB3ΔgenB2	ND	ND	ND	ND	ND	2.3	ND	142.0	ND

Table S2, related to Experimental Procedures. Plasmids used in this study

Plasmid	Description	Reference
pUC18	Sub-cloning vector	Sambrook and Maniatis
pET28a(+)	Vector for protein expression	Invitrogen
pIB139	Site-specific integrative vector with P*ermE** promoter	Wilkinson et al., 2002; Del Vecchio et al., 2003
pYH7	*E. coil* - *Streptomyces* shuttle vector	Sun et al., 2008
pWHU77	pIB139 derivative with Tsr^R	This study
pYH286	*genQ* in-frame deletion construct	This study
pWHU1	*genK* in-frame deletion construct	This study
pWHU67	ΔgenK complementation construct containing *genK* under control of P*ermE** promoter	This study
pWHU163	ΔgenQ complementation construct containing *genQ* under control of P*ermE** promoter	This study
pWHU165	ΔgenQ complementation construct containing *neo11* under the control of the P*ermE** promoter	This study
pWHU4	*genB1* in-frame deletion construct	This study
pWHU2	*genB2* in-frame deletion construct	This study
pWHU5	*genB3* in-frame deletion construct	This study
pWHU3	*genB4* in-frame deletion construct	This study
pWHU43	*genB3* and *genB4* double in-frame deletion construct	This study
pET28/LM_genQ	pET28a(+) vector with VM_*genQ* gene insert	This study
pET28/VM_genQ	pET28a(+) vector with LM_*genQ* gene insert	This study
pET28/genB1	pET28a(+) vector with *genB1* gene insert	This study
pET28/genB2	pET28a(+) vector with *genB2* gene insert	This study
pET28/genB3	pET28a(+) vector with *genB3* gene insert	This study
pET28/genB4	pET28a(+) vector with *genB4* gene insert	This study

Table S3, related to Experimental Procedures. In-frame deletion mutants and complemented strains used in this study

Gene knockout mutant	Parent strain	Plasmid used to achieve deletion
ΔgenK	wild type	pWHU1
ΔgenQ	wild type	pYH286
ΔgenQΔgenK	ΔgenQ	pWHU1
ΔgenB1	wild type	pWHU4
ΔgenB2	wild type	pWHU2
ΔgenB3	wild type	pWHU5
ΔgenB4	wild type	pWHU3
ΔgenB2ΔgenB1	ΔgenB2	pWHU4
ΔgenB1ΔgenB3	ΔgenB1	pWHU5
ΔgenB4ΔgenB3	ΔgenB4	pWHU43
ΔgenB2ΔgenB3	ΔgenB2	pWHU5
ΔgenB2ΔgenB4	ΔgenB2	pWHU3
ΔgenB4ΔgenB1	ΔgenB4	pWHU4
ΔgenB4ΔgenB1ΔgenB2	ΔgenB4ΔgenB1	pWHU2
ΔgenB4ΔgenB1ΔgenB3	ΔgenB4ΔgenB1	pWHU43
ΔgenB2ΔgenB3ΔgenB1	ΔgenB2ΔgenB3	pWHU4
ΔgenB2ΔgenB4ΔgenB3	ΔgenB2ΔgenB4	pWHU43
ΔgenB4ΔgenB1ΔgenB3ΔgenB2	ΔgenB4ΔgenB1ΔgenB3	pWHU2
**Complemented mutant**	**Parent strain**	**Plasmid for complementation**
ΔgenK::*genK*	ΔgenK	*genK* gene in pWHU77
ΔgenQ::*genQ*	ΔgenQ	*genQ* gene in pWHU77
ΔgenQ::*neo11*	ΔgenQ	*neo11* gene in pWHU77
